# An artificial intelligence-driven synthesis planning platform (PhotoCat) for photocatalysis

**DOI:** 10.1038/s42004-026-01894-y

**Published:** 2026-01-21

**Authors:** Jiangcheng Xu, Silong Zhai, Panyi Huang, Wenbo Yu, Qingyi Mao, Kui Du, Weike Su, Bin Sun, Can Jin, An Su

**Affiliations:** 1 National Engineering Research Center for Process Development of Active Pharmaceutical Ingredients, Collaborative Innovation Center of Yangtze River Delta Region Green Pharmaceuticals, Zhejiang University of Technology, Hangzhou, P. R. China; 2 Hangzhou Polytechnic University, Hangzhou, P. R. China; 3 Faculty of Applied Science, Macao Polytechnic University, Macau SAR, P. R. China; 4 Institute of Advanced Studies and School of Pharmaceutical Sciences, Taizhou University, Jiaojiang, P. R. China; 5 School of Chemistry and Chemical Engineering, Shaoxing University, Shaoxing, P. R. China; 6 Zhejiang Key Laboratory of Green Manufacturing Technology for Chemical Drugs, College of Pharmaceutical Sciences, Zhejiang University of Technology, Hangzhou, P. R. China

**Keywords:** Cheminformatics, Photocatalysis

## Abstract

While photocatalysis has emerged as a transformative tool in modern synthesis, AI-assisted reaction prediction faces significant challenges due to data limitations. We present PhotoCatDB - a curated, open-source database containing 26.7 K photocatalytic reactions with detailed mechanistic annotations, including 9.2 K multicomponent transformations. Leveraging this resource alongside 100 million molecular data points, we developed PhotoCat, a Transformer-based platform that achieves unprecedented accuracy in photocatalytic reaction prediction (82.6%), retrosynthesis (77.1%), and condition recommendation (88.5%). The platform’s capabilities were experimentally validated through the discovery of four novel photocatalytic reactions with yields up to 75.3%. This integrated approach establishes a new paradigm for data-driven innovation in photocatalysis, bridging computational prediction with experimental validation to accelerate discovery in sustainable chemistry.

## Abstract (web summary)

Photocatalysis holds promise for sustainable synthesis, but AI-driven reaction prediction is hindered by limited data. Here, the authors introduce PhotoCatDB, a comprehensive database of 26.7 K reactions, and develop PhotoCat, a transformer-based tool achieving high accuracy in photocatalytic reaction prediction, retrosynthesis, and condition recommendation, as well as experimentally validate the newly discovered photocatalytic reactions.

## Introduction

Photocatalytic reactions, which harness visible light or solar energy as a driving force, offer a green and sustainable approach to chemical synthesis^1,2^. In recent years, with advancements in photocatalyst optimization^3,4^ and deeper insights into reaction mechanisms^5^, photocatalysis has made significant progress in organic synthesis^2,6^, particularly in radical reactions^7,8^, redox transformations^9–11^, and cross-coupling^12,13^ processes. In this context, photocatalysis represents a breakthrough that inspires chemists to explore uncharted territories and discover elusive reaction patterns^14,15^. Despite these advances, the practical execution of photocatalysis in the lab is fraught with challenges, often necessitating years of dedicated research to discover and optimize each novel photocatalytic reaction^14^.

Deep learning models have made great strides in recent years, largely due to their remarkable ability to extract knowledge from massive amounts of data^16–20^. In the field of organic synthesis, deep learning has brought about a revolution that has impacted several areas, including forward reaction prediction^21–28^, retrosynthesis planning^29–33^, mechanistic inference^34,35^, inferring experimental procedures^36,37^, reaction yield prediction^38^, and new reaction development^39–41^. Specifically, deep learning models have proven to be effective in predicting specific types of reactions, including enzyme-catalyzed reactions^28,42^, carbohydrate reactions^43^, and electrochemical reactions^44^. In addition, the application of deep learning in the field of photocatalysis has garnered significant attention, particularly in the design and optimization of photocatalysts^45,46^.

However, to the best of our knowledge, no deep learning models specifically targeting photocatalytic reactions have been reported, not only because of the challenging nature of exploring photocatalytic reactions^14,15^ that leads to the scarcity of photocatalytic reaction data, but also due to the limitation of currently available reaction databases. Although acknowledged chemical reaction databases like Reaxys^47^, SciFinder^48^, can offer access to photocatalytic reactions via keyword searches, they suffer from incomplete reaction condition information. For instance, the water as a reactant of photocatalysis is hidden in the word “wet”, which was missed by the Reaxys dataset (Reaction ID: 49168884). Another initiative to address the database format issue is the Open Reaction Database (ORD) framework proposed by Kearnes et al. ^49^. ORD displays chemical reaction data in a structured format, which provides strong support for machine learning prediction of chemical reactions. However, with fewer than 300 photocatalytic reactions recorded, ORD lacks the volume of data required to effectively train deep learning models in this field.

**Fig. 1:**
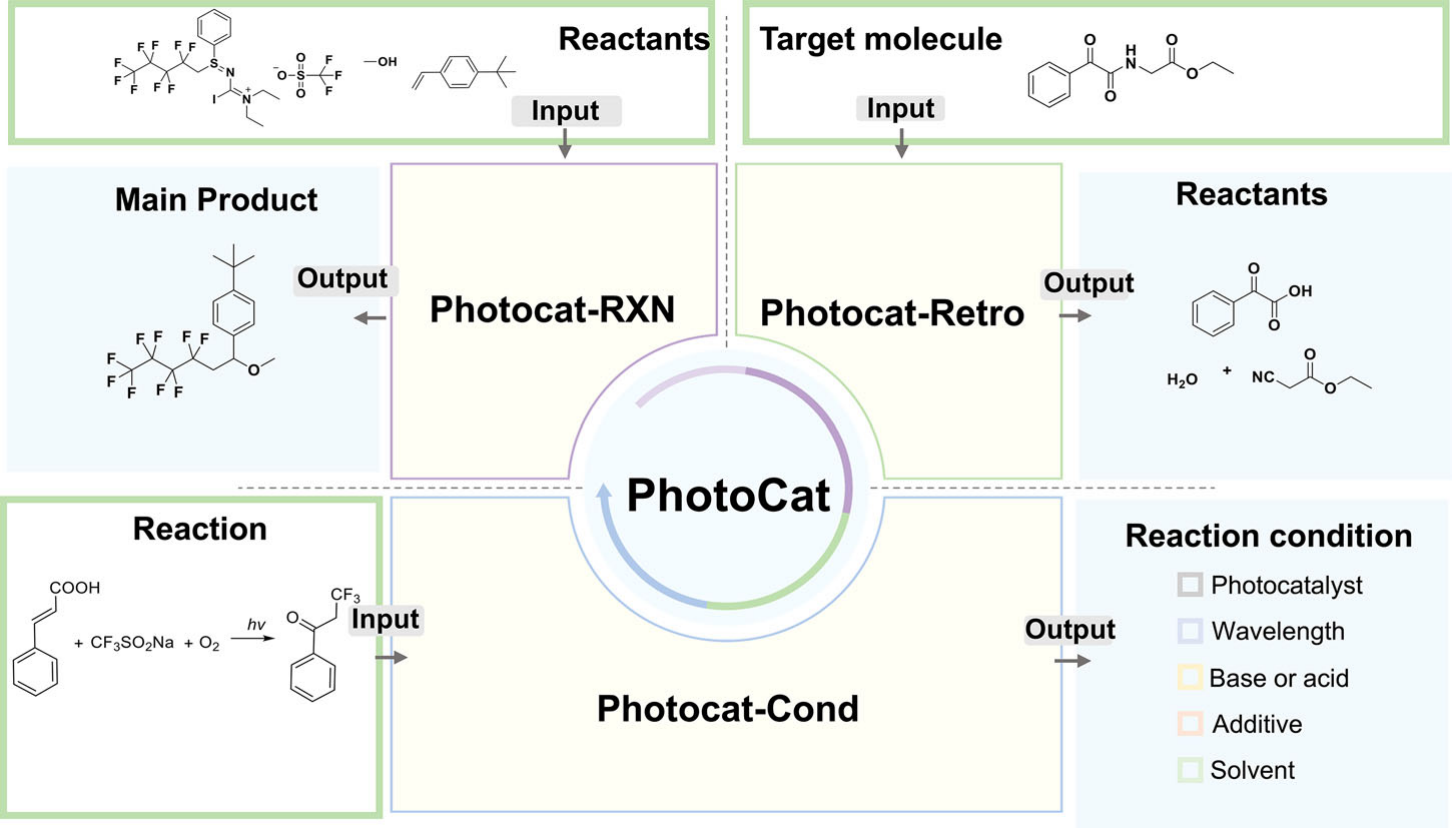
PhotoCat comprises three modules: PhotoCat-RXN for photocatalytic reaction prediction, PhotoCat-Retro for retrosynthesis, and PhotoCat-Cond for condition recommendation. Using data-driven (in-silico) approaches, it accelerates the discovery and optimization of photocatalytic reactions in wet-lab settings.

In this study, a database of photocatalytic reactions, named PhotoCatDB, was established through a comprehensive literature search, and these reactions were scrutinized by human experts. To further enrich the database, we also incorporated experimentally recorded reaction data. Most importantly, we added the essential reaction conditions, such as photocatalysts, bases or acids, additives, wavelength, and solvents, to PhotoCatDB to accurately reflect the nuances of real-world laboratory settings. Building upon this robust database, we developed PhotoCat, an advanced transformer-based deep-learning platform for predicting photocatalytic reactions, conducting retrosynthesis, and recommending reaction conditions (Fig. 1). We used PhotoCat to successfully identify and experimentally validate four previously unreported photocatalytic reactions of practical significance. This study marks significant progress in predicting photocatalytic reactions and provides a powerful tool to accelerate the discovery and validation of novel photocatalytic reactions with diverse applications.

## Results and discussion

### PhotoCatDB, a photocatalytic reaction database

**Fig. 2:**
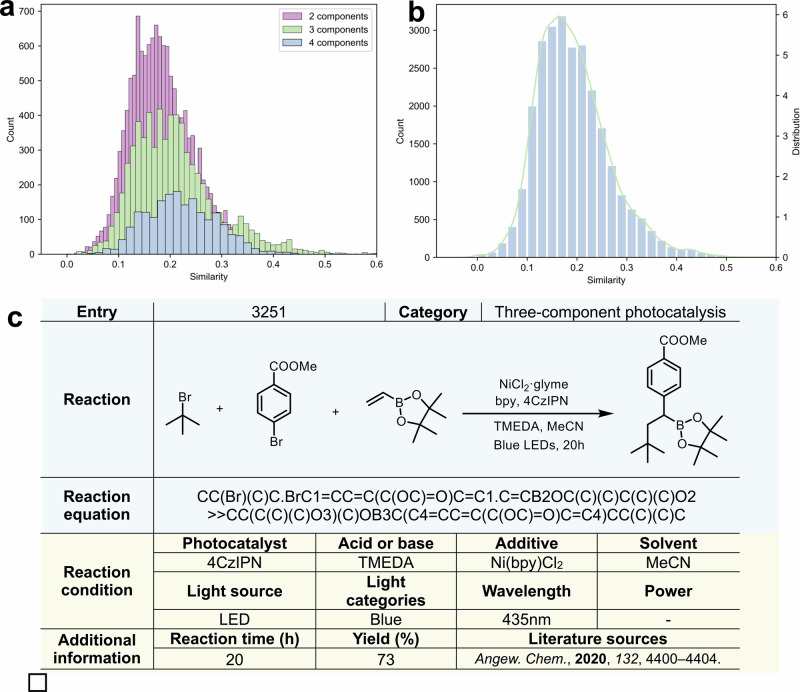
Analysis of the data distribution and composition of PhotoCatDB. **a** Multicomponent reactions comprise 34.46% of the dataset, with three-component and four-component reactions marked in green and blue, respectively. **b** The dataset displays a skewed distribution, with most data points falling within the lower similarity range (below 0.21). **c** An example of PhotoCatDB data entry, illustrating the format and structure of the dataset.

In our efforts to build a unique and valuable resource, we have gathered an extensive collection of photocatalytic reactions. Multicomponent reactions present greater chemical challenges and, from a machine learning perspective, generate larger and sparser combinatorial spaces that limit model generalization. The workflow for constructing PhotoCatDB is shown in Supplementary Fig. 1. PhotoCatDB currently contains 26.7K validated photocatalytic reactions, including 9.2K multicomponent reactions, of which 6708 are three-component, and 2455 are four-component cases (Fig. 2a). Among these multicomponent reactions, 6523 were further annotated by categorizing their conditions through manual mechanistic analysis of the primary literature. To ensure a diverse representation of photocatalytic reactions and to prevent redundancy, we have carefully managed the inclusion of similar reactions. This is demonstrated in the distribution of Tanimoto similarity^50^ scores of the product molecules within the database (Fig. 2b). The data show a predominance of unique reactions, with most Tanimoto similarity scores falling below 0.2, across a range from 0 to 0.5. This skewed distribution underscores our commitment to maintaining a database that promotes the discovery of novel photocatalytic processes by minimizing overlap and maximizing the breadth of reaction scenarios covered.

Each reaction record in PhotoCatDB contains the following key components: (1) Reaction equations represented using simplified molecular input line entry system^51^ (SMILES), a specialized notation for expressing chemical structures in a computer-readable format. In this notation, the symbol “»” separates reactants and products, while the symbol “.” distinguishes different reactants. (2) Additional information, including reaction time, yield, and literature sources. To further capture the unique features of photocatalytic reactions, we have created a focused subset within the database, PhotoCatDB-Cond (PhotoCatDB-Condition), which includes 6523 photocatalytic reactions. Each entry also contains (3) Reaction conditions, which are categorized into five essential elements based on a manual analysis of the reaction mechanisms: *photocatalyst*, *base or acid*, *additives*, *solvent*, and *wavelength*. The detailed definitions of these categories and the corresponding classification criteria are provided in Supplementary Table 1. Some reactions, such as those involving quinoxalinone, azobenzene, or EDA complexes, do not require an external photocatalyst because the reactants themselves act as photosensitizers. The *photocatalyst* for these reactions is classified as “*autocatalysis*”. The reaction conditions cover a broad range, including 59 photocatalysts, 34 bases or acids, 53 additives (of which 37 are ligands), and 42 solvents. An example of a PhotoCatDB entry is shown in Fig. 2c, with additional examples available in Supplementary Tables 2–7.

**Fig. 3:**
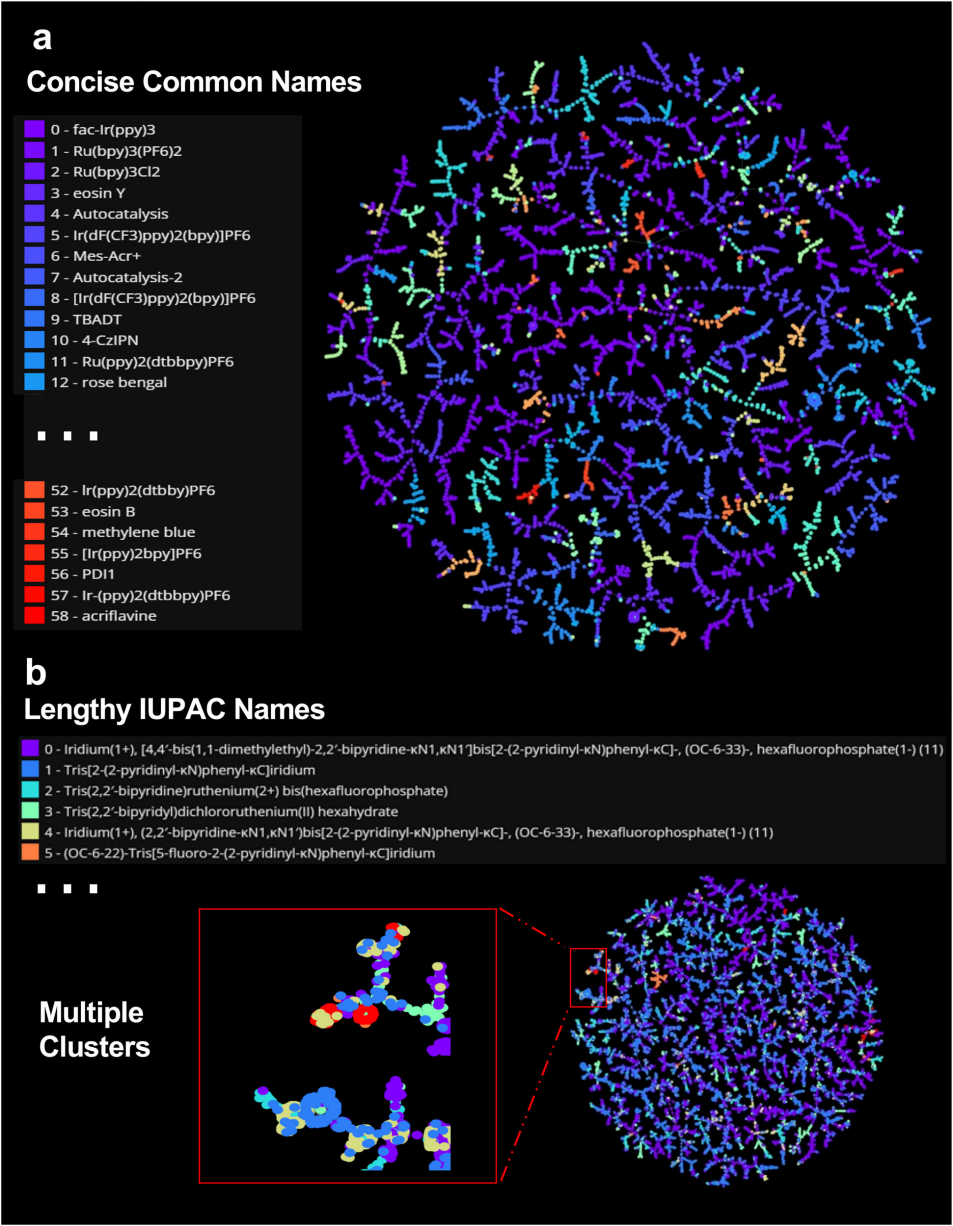
Comparison of PhotoCatDB and SciFinder-Photocatalysis (the photocatalysis dataset from SciFinder). **a** PhotoCatDB uses common names for photocatalysts, while **b** SciFinder-Photocatalysis employs lengthy IUPAC names. In this tree-map embedding, each point denotes a reaction, with spatial proximity indicating feature similarity. Clusters correspond to chemically related reactions, while isolated points highlight unique transformations, providing an overview of the dataset’s feature space. **a** Does not show aggregation of similar reactions as “multiple clusters” as in (**b**).

For a more insightful analysis of the dataset, TMAP^52^, a tree-based unsupervised learning algorithm, was utilized to visualize the chemical reaction mapping of the PhotoCatDB (Fig. 3a). In this tree-map embedding, each point represents a reaction, and spatial proximity indicates similarity in reaction features. Clusters show chemically similar reactions, while isolated points denote unique transformations, providing an overview of the dataset’s distribution in feature space. Despite the wide variety of photocatalysts, clustering of reactions catalyzed by the same photosensitizer was observed. Notably, different clustering was observed when reactions were colored according to other types of reaction conditions, such as bases or acids, additives and solvents (Supplementary Figs. 2–4), which demonstrates the multivariate effect of reaction conditions and suggests the necessity to include multiple components of reaction conditions in the database. Further discussion of dataset bias, diversity, and limitations is provided in Section 1.5 of the Supplementary Information.

To further demonstrate the advantages of the PhotoCatDB, the photocatalytic reactions collected from SciFinder were also visualized using TMAP (Fig. 3b). PhotoCatDB utilizes common names to represent complex photocatalyst structures, simplifying data reading and storage. In contrast, SciFinder-Photocatalysis uses IUPAC names, which are more challenging for chemical researchers to classify and comprehend. Meanwhile, PhotoCatDB-Cond showcases a greater diversity in reaction types, preventing the aggregation of similar reactions observed in SciFinder-Photocatalysis. PhotoCatDB was curated independently from academic literature, restricted to photocatalytic transformations, and shows no overlap with the USPTO reaction set (Section 1.6 of Supplementary Information). One limitation of the current study is that PhotoCatDB does not yet include fine-grained reaction class annotations; future extensions of the database will address this to enable more systematic performance analysis.

### PhotoCat-RXN, a reaction-prediction model trained on USPTO and PhotoCatDB

**Fig. 4:**
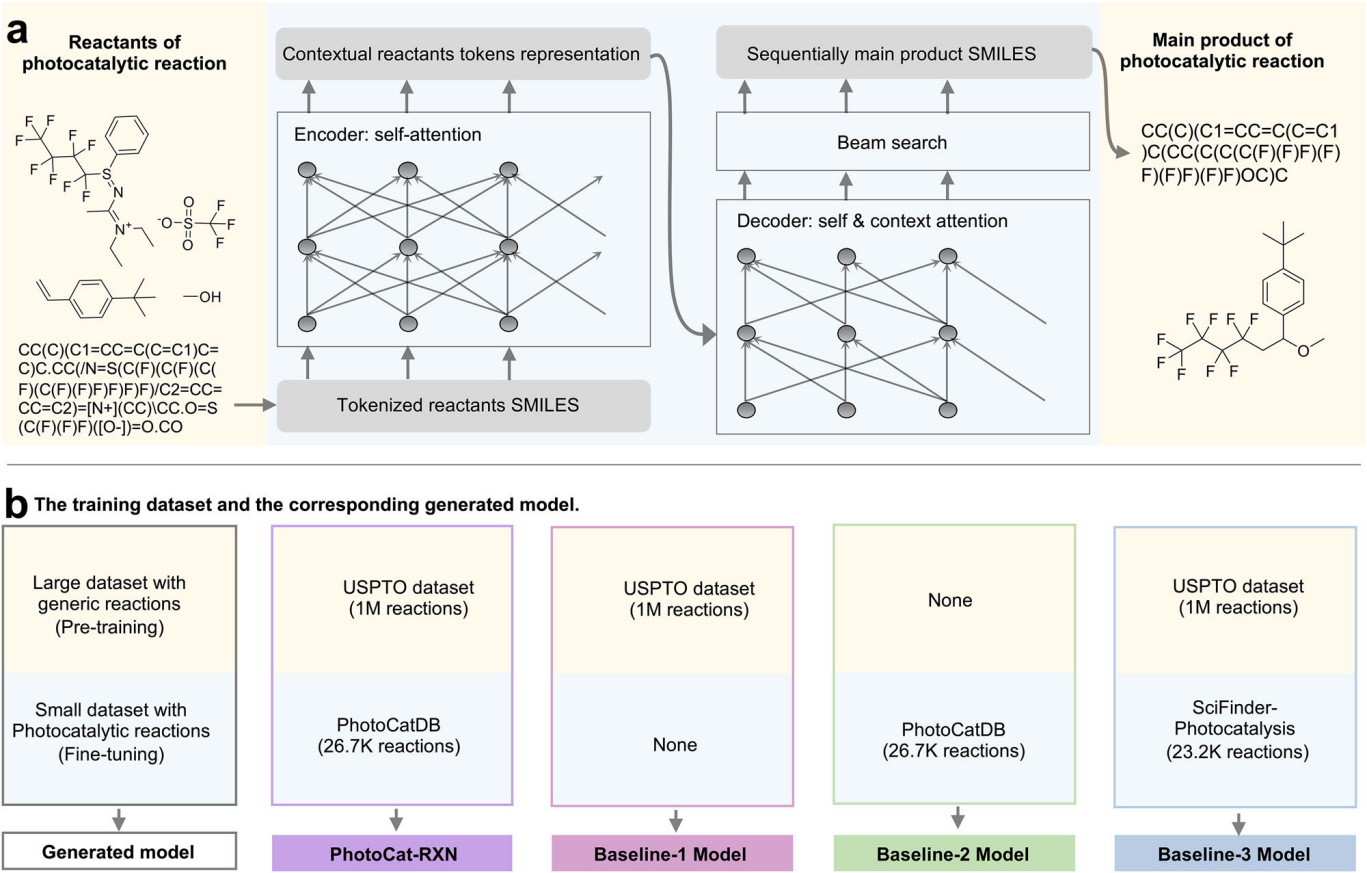
The transformer model and training schematic. **a** Sequence-to-sequence prediction of photocatalytic reactions and **b** the training schematic of PhotoCat and three baseline models.

PhotoCat-RXN uses a Transformer-based model from the OpenNMT framework^41,53^ as the basis of its model architecture (Fig. 4). First, the model was pre-trained using the USPTO database containing 1 million instances of chemical reactions to gain basic knowledge of chemical reactions. After that, the model was fine-tuned using PhotoCatDB. To demonstrate the need for transfer learning, we trained the Baseline-1 model exclusively on USPTO, while the Baseline-2 model was trained solely on PhotoCatDB. It is important to note that to fairly compare the role of the reactions in USPTO and PhotoCatDB in model training, the PhotoCatDB data for training in this section does not contain any reaction conditions.

**Fig. 5:**
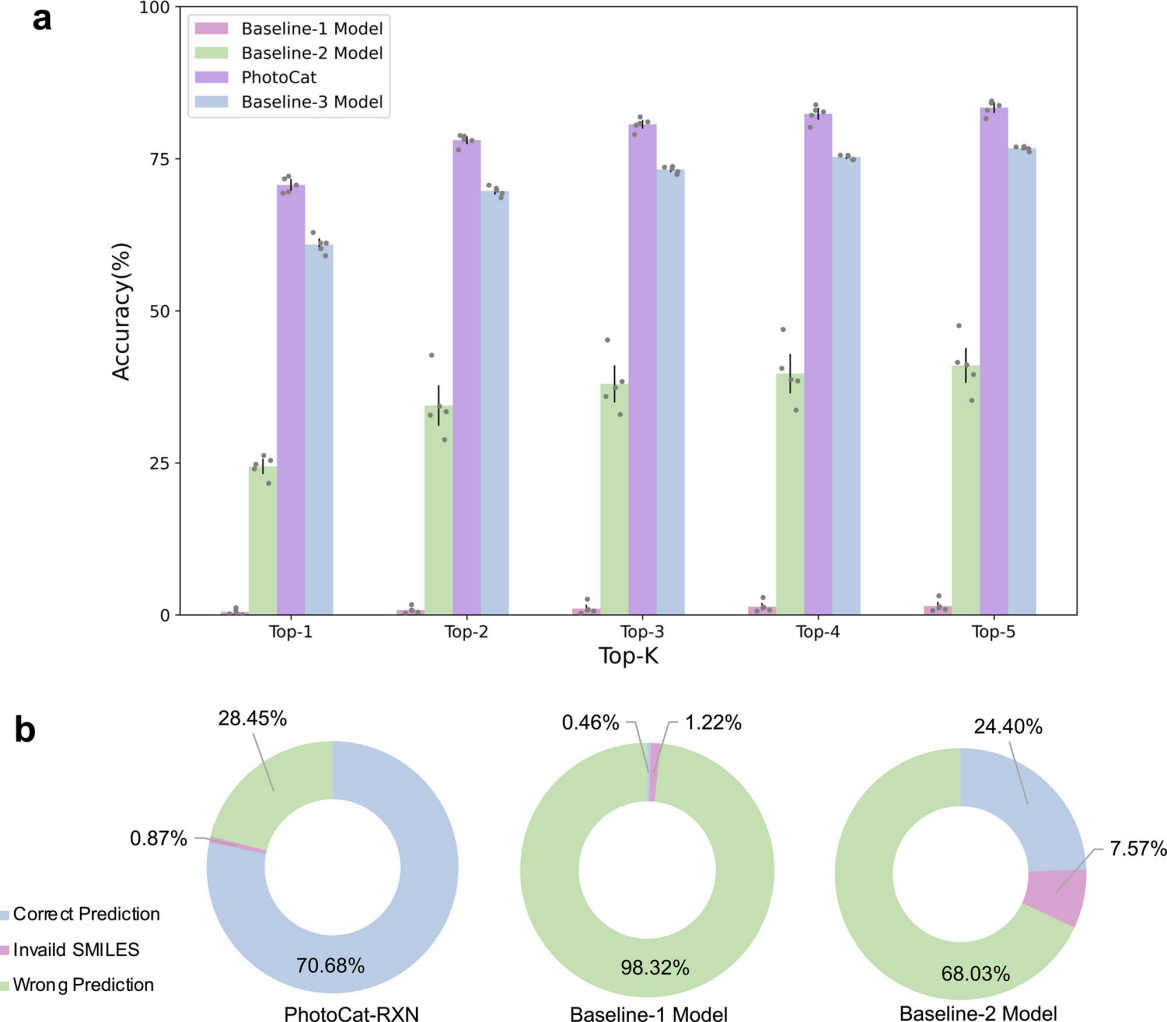
Comparison of prediction accuracies of PhotoCat and baseline models. **a** The comparison of Top-1 to Top-5 accuracies. **b** Percentage of invalid SMILES and incorrect predictions in Top-1 accuracies for Baseline-1 and -2 models and PhotoCat.

As shown in Fig. 5a and Supplementary Tables 7–-10, the average Top-1 accuracy of the pre-trained and fine-tuned PhotoCat was 70.68%, and the Top-2 to Top-5 accuracies were all above 78%. In contrast, the prediction accuracy of the Baseline-1 model trained only on USPTO was significantly lower, with an average Top-1 accuracy of only 0.46% and similar low Top-2 to Top-5 accuracies. We also used a different USPTO-pretrained reaction prediction model by Zhong et al.^54^ to directly predict the reactions of PhotoCatDB, and the Top-1 accuracy was only 0.69% (Supplementary Table 11). In addition, the Baseline-2 model, trained solely on PhotoCatDB, also exhibited lower prediction accuracies than PhotoCat. Figure 5b offers an alternative perspective on this comparison, demonstrating that the proportion of invalid SMILES predicted by PhotoCat-RXN (0.87%) is lower than that of the Baseline-1 and Baseline-2 models. The results above suggest that the USPTO provides the necessary information for the model to understand chemical reactions, while PhotoCatDB provides the model with the ability to predict photocatalytic reactions.

### Reaction conditions improve accuracy and efficiency of reaction prediction

**Fig. 6:**
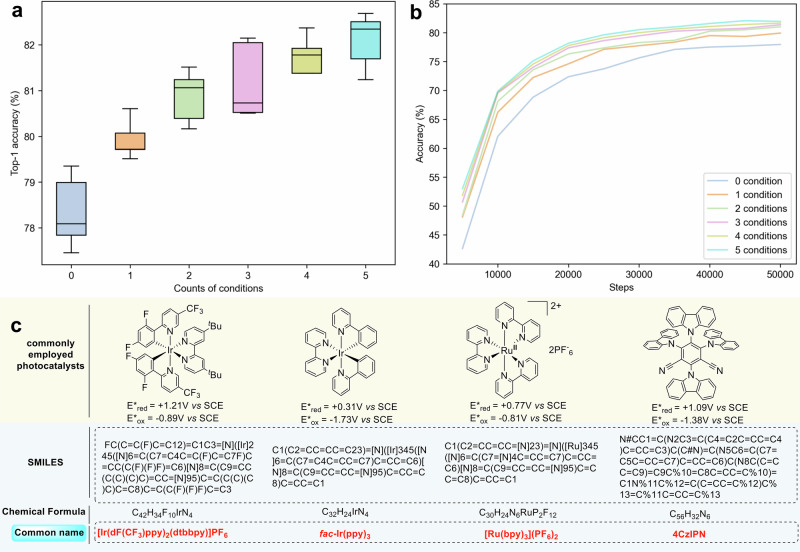
Effects of reaction condition inputs on model predictions and training efficiency. **a** As the quantity of input reaction conditions grows, there’s a consistent ascent in prediction accuracy. **b** The adaptive inclusion of various reaction condition inputs significantly amplifies the model’s training agility. **c** Simplification strategy for describing reaction conditions in PhotoCatDB-Cond.

After further fine-tuning PhotoCat-RXN using the PhotoCatDB-Cond subset, we found that incorporating reaction conditions improved the top-1 average accuracy of PhotoCat-RXN from 78.2% to 82.3%, an increase of 4.1% (Fig. 6, Supplementary Tables 12-16). We further investigated the effect of varying the number of reaction conditions on prediction accuracy. The analysis began with only the *photocatalyst* and then sequentially incorporated the *base or acid*, *additives*, *wavelength*, and *solvent*. As the number of input reaction conditions increased, the accuracy of reaction predictions continued to rise (Fig. 6a). PhotoCat achieved predicted Top-1 accuracies of 78.1%, 79.8%, 81.9%, and 82.1% when training included one, two, three, and four reaction conditions, respectively. All these accuracies surpassed those of the fine-tuned model that did not utilize information about the reaction conditions. We also extended our evaluation to a graph-based architecture (Graph2SMILES^55^), which similarly benefited from incorporating reaction conditions, confirming that the improvement is not specific to the Transformer model (Supplementary Fig. 10).

Integrating reaction conditions also speeds up the training process. Figure 6b shows that the training curve grows faster for the models with more reaction conditions provided. With no reaction conditions (blue), the accuracy reaches about 76% after 40,000 training steps. In contrast, when all five reaction conditions are considered (cyan), the training accuracy grows faster, reaching over 80% after only 30,000 steps. These observations demonstrated the importance of incorporating detailed and comprehensive reaction conditions during the training and prediction phases, which promotes superior model performance and faster convergence to peak prediction accuracy.

Unlike previous studies that considered only a few commonly used reagents^21–24,56,57^ or ignored reaction conditions altogether^26,58^, this study shows that including reaction conditions can improve the predictive accuracy and training efficiency of chemical reaction prediction models. There are two main reasons for this improvement. First, in previous studies that considered reaction conditions, the reactants were input simultaneously as mixtures, which may increase the challenge of the model in interpreting the reaction conditions. In this study, the photocatalytic reaction conditions were methodically grouped into five different types, significantly reducing the complexity of the model in grasping the intricate chemical reaction conditions. In addition, the terminology of the reaction conditions was simplified by using concise common names or identifiers, which establishes a direct correspondence between the structure of the reagents and their names (Fig. 6c) and simplifies the model training.

**Fig. 7:**
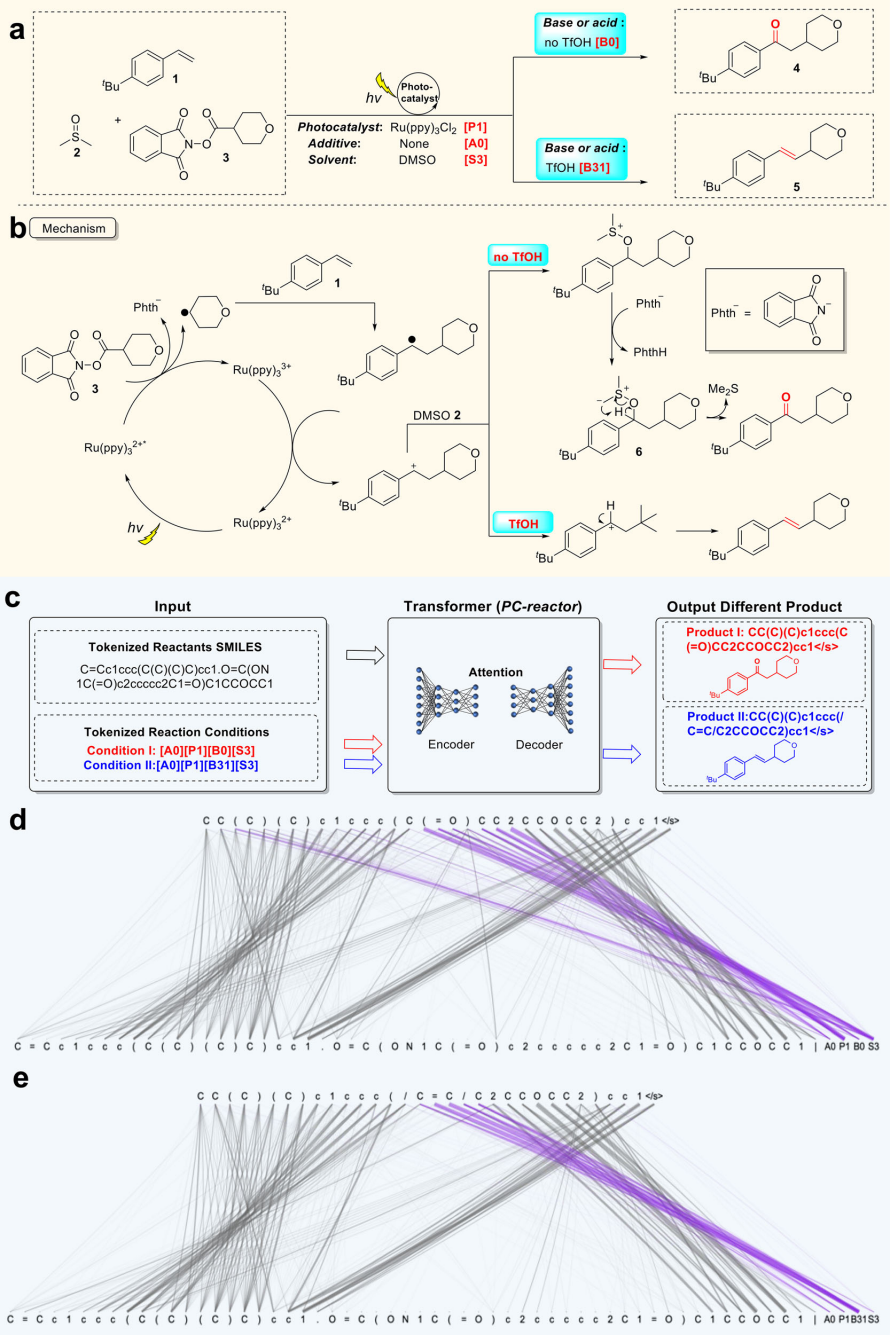
Interpretability and attentional analysis of PhotoCat-RXN. **a** The presence or absence of trifluoromethanesulfonic acid in the reaction system dictates the main product outcome^61^, producing aldehyde **4** or alkene **5**. A plausible reaction mechanism is presented in (**b**). **c** PhotoCat accurately predicts main products when provided with corresponding reaction conditions (B0 and B31, respectively refer to “Base or acid” being “None” and “TfOH”). In the attention heatmaps **d** and **e**, the upper strings represent the SMILES of the main product from the photocatalytic reaction (output), while the lower strings denote the SMILES of the reactants and the input of the four reaction conditions. The output of the product’s SMILES emphasizes the input of the four reaction conditions (highlighted in purple). Notably, when the key functional group of ketone carbonyl “C( = O)” (**d**) or alkene “C = C” (**e**) is outputted, PhotoCat pays special attention to the corresponding inputs of B0 and B31, respectively.

The inclusion of reaction conditions improves prediction accuracy by providing critical contextual information that can fundamentally alter photocatalytic outcomes^59,60^ (see Section 5.3 of the ESI). Case analysis (Fig. 7) demonstrates that incorporating classified and simplified reaction conditions into model training enhances reaction prediction accuracy while ensuring interpretability. When the reactants (styrene **1**, DMSO **2**, NHP ester **3**) are subjected to a trivalent ruthenium photocatalyst and light, ketone **4** is the main product in the absence of acid^61^. However, when a strong acid (e.g., trifluoromethanesulfonic acid) is present, the reaction shifts to alkene **5** as the main product (Fig. 7a). A high concentration of protons prevents the deprotonation of the alkoxysulfonium intermediate **6**, thus hindering the formation of ketone **4**^61^ (Fig. 7b). Based on complete reaction conditions, PhotoCat precisely predicts the main product (Fig. 7c). In this case, “B0” indicates the absence of “base or acid”, while “B31” represents the inclusion of trifluoromethanesulfonic acid in the reaction conditions. In the attention heatmap, the thickness of the lines represents the attention weight between the input and output. Figure 7d and e shows that PhotoCat clearly focuses on the input reaction conditions (highlighted in purple) when predicting the main product. Notably, PhotoCat gives greater attention to “B0” when outputting the key ketone carbonyl group “C = O” (Fig. 7d), and “B31” when projecting the critical alkene group “C = C” (Fig. 7e). This is consistent with human experts’ approach in determining the main products of photocatalytic reactions based on reaction conditions.

### PhotoCat-Retro, a photocatalytic retrosynthesis model

**Fig. 8:**
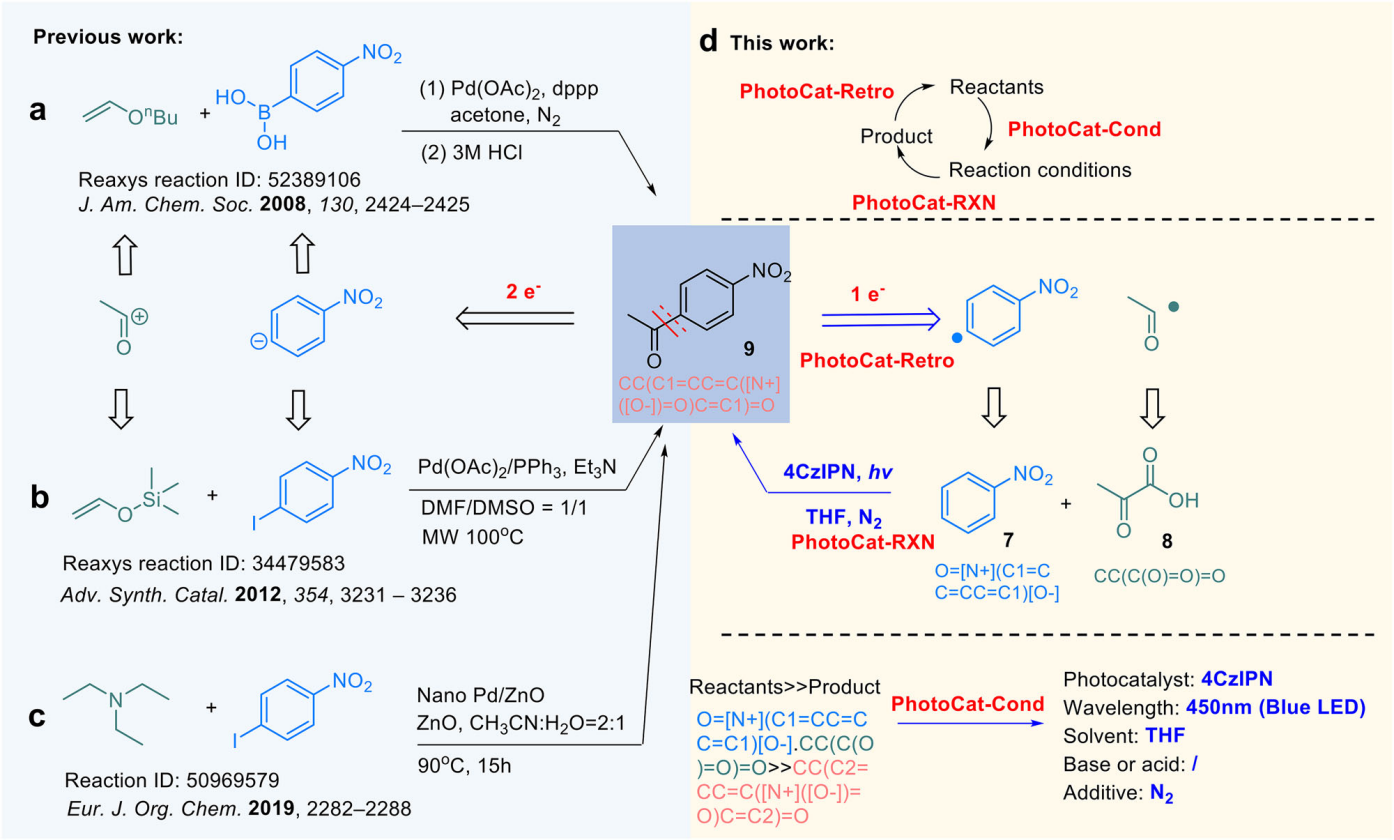
Comparison of conventional synthetic strategies and the photocatalytic retrosynthetic pathway proposed by PhotoCat-Retro. **a**–**c** Representative literature-reported approaches for aromatic ketone **9** synthesis based on Pd-catalyzed two-electron acylation strategies. **d** The retrosynthetic disconnection proposed by PhotoCat-Retro, featuring a photocatalytic radical acylation between nitrobenzene **7** and pyruvic acid **8**, which was subsequently validated experimentally.

The photocatalytic retrosynthesis module (PhotoCat-Retro) complements the reaction prediction module (PhotoCat-RXN) by generating feasible photocatalytic pathways directly from target molecules, thereby expanding the space of reactions that can be explored. This capability is especially valuable for compounds without reported forward reaction data. As shown in Fig. 8a–c, traditional syntheses of aromatic ketones usually rely on Pd-catalyzed two-electron acylation using prefunctionalized aryl halides or boron reagents^62–64^. For the same target molecule, PhotoCat-Retro automatically proposed a non-obvious radical acylation disconnection in which nitrobenzene and pyruvic acid directly couple to introduce the acyl group (Fig. 8d). This reflects the model’s ability to capture key mechanistic patterns in photocatalytic radical processes. After refinement by PhotoCat-Cond and confirmation by PhotoCat-RXN, the proposed pathway was successfully validated experimentally (Section 3.4 of the ESI). This example illustrates the practical value of PhotoCat-Retro in generating experimentally viable and innovative photocatalytic disconnection strategies.

**Fig. 9:**
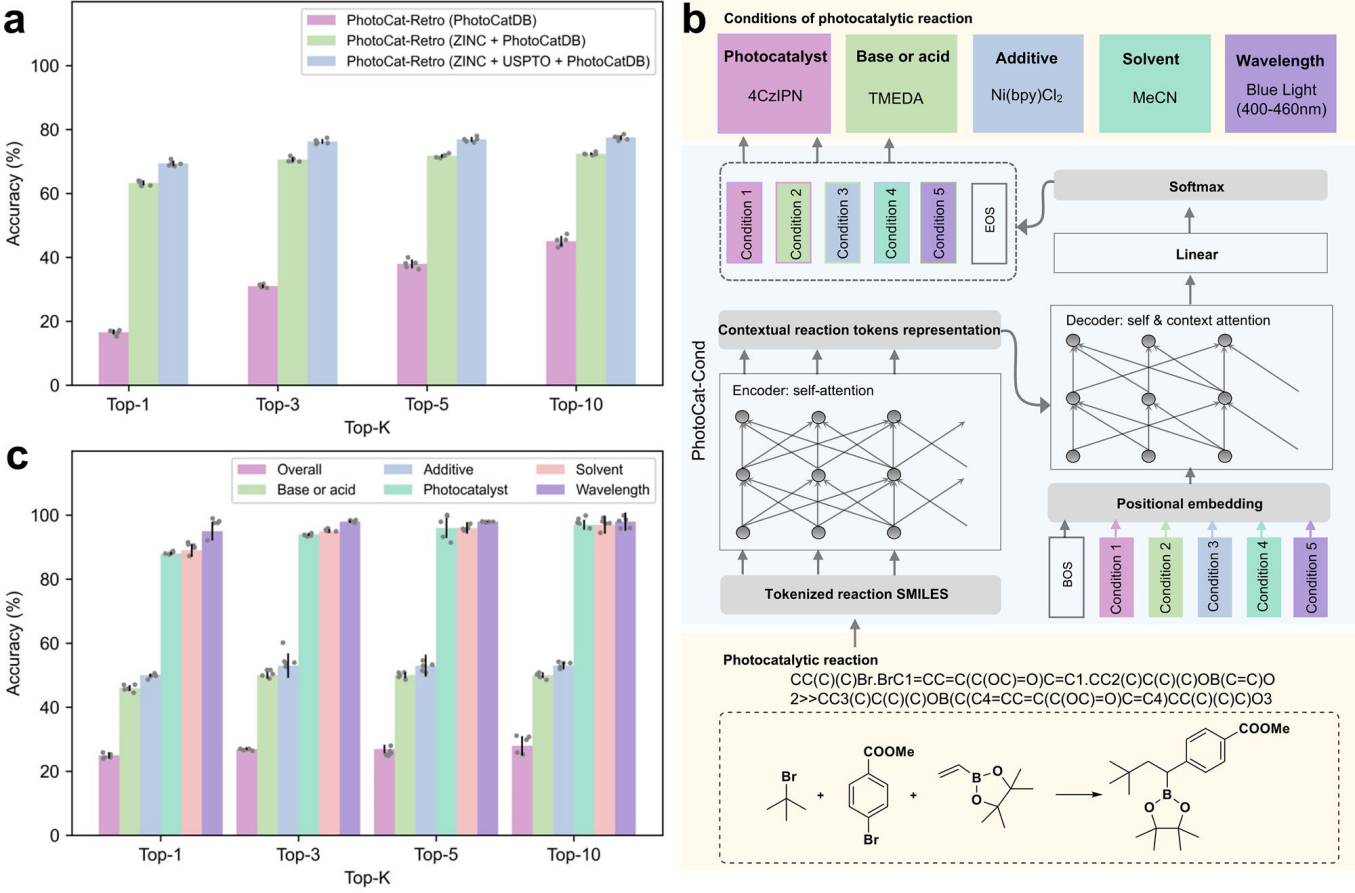
PhotoCat includes features for photocatalytic reaction retrosynthesis (PhotoCat-Retro) and reaction condition recommendation (PhotoCat-Cond). **a** With the help of transfer learning from the ZINC molecular database and the USPTO reaction database, PhotoCat-Retro shows a significant improvement in the accuracy of reaction retrosynthesis. **b** A Transformer-based model specifically developed for the recommendation of optimal conditions in photocatalytic reactions. **c** Test results of PhotoCat-Cond.

PhotoCat-Retro utilizes a state-of-the-art approach, adopting a training strategy inspired by the work of Irwin et al.^65^. To evaluate optimal training strategies, we experimented with three conditions: no pretraining, pretraining at the atom level, and pretraining at the atom and reaction level. The results from 5-fold cross-validation (Fig. 9a, Supplementary Tables 17–19) show that when trained solely on PhotoCaDB for retrosynthesis analysis, the Top-1 average accuracy is 16.60%. However, after incorporating transfer learning from the ZINC database^66^ and the combined ZINC + USPTO datasets, the top-1 accuracy improved to 63.28%, 69.43%, a significant increase of 52.83%. This demonstrates that the transfer learning strategy with the ZINC molecular database and the USPTO reaction database during PhotoCat-Retro training helps address the data limitations of PhotoCatDB.

In comparison, current state-of-the-art retrosynthesis models, such as NAG2G^67^, EditRetro^68^, and LocalRetro^69^, achieve notable performance on general retrosynthesis tasks using benchmark databases like USPTO-50k, with average Top-1 accuracies ranging from 57.4% to 67.2%. However, these results remain inferior to the Top-1 accuracy attained by PhotoCat-Retro specifically for photocatalytic retrosynthesis analysis, highlighting its exceptional capability in handling complex photocatalytic reactions. On the other hand, Chemformer demonstrates Top-1 to Top-10 accuracies ranging from 54.3% to 63.0% on the benchmark USPTO-50k dataset (with known chemical reaction classification). After being fine-tuned on the PhotoCatDB, Chemformer exhibits significantly improved performance in photocatalytic organic synthesis retrosynthesis tasks, underscoring the importance and effectiveness of domain-specific training.

### PhotoCat-Cond, a deep learning model for recommending photocatalytic reaction conditions

Recommending reaction conditions is essential for improving reaction efficiency and reproducibility. Building on the work of Yao’s research group^70^ and others^71,72^, this study utilizes curated reaction condition data from the PhotoCatDB to develop PhotoCat-Condition (PhotoCat-Cond), a Transformer-based model (Fig. 9b) specifically designed to recommend optimal conditions for photocatalytic reactions. A key feature of PhotoCat-Cond lies in its ability to categorize diverse photocatalytic systems and make condition-specific predictions within each category.

Results from 5-fold cross-validation (Fig. 9c, Supplementary Fig. 8, Supplementary Tables 21–25) demonstrate the robust performance of PhotoCat-Cond. The model accurately recommends the crucial light categories (wavelength), achieving an average Top-1 prediction accuracy of 94.82%. For the particularly challenging task of predicting photosensitizers with complex conjugated structures, the model, based on simplified name representations, achieved an average top-1 accuracy of 88.50%. In terms of recommending solvents, additives, and acids or bases, the model reached top-1 accuracies of 89.52%, 50.06%, and 46.12%, respectively. Notably, in 28.26% of the test cases, PhotoCat-Cond correctly predicted all five conditions of the input reaction simultaneously through an iterative process.

PhotoCat-Cond achieves accuracy comparable to state-of-the-art condition-recommendation models reported in other domains, though direct comparison is not possible due to differences in training datasets and reaction types (Supplementary Table 26). Representative models include Parrot-LM-E^70^ (Top-1 accuracies of 92.5% for catalysts and 50.2% for solvents on the USPTO-cond dataset), AR-GCN^71^ (64.9% for catalysts, 90.8% for ligands, and 72.2% for solvents on the Suzuki coupling dataset), and CIMG-Condition^72^ (59.0% for catalysts and 93.0% for solvents on a general reaction dataset). These results indicate that PhotoCat-Cond performs competitively within its specialized photocatalytic domain, highlighting the advantages of domain-specific datasets, such as PhotoCatDB, and supporting the effectiveness of our targeted strategy for predicting complex photocatalytic conditions.

In this study, reagents and conditions were represented using standardized common names rather than SMILES. While SMILES provides an unambiguous molecular representation, we selected common names because (i) they are the dominant form used in experimental sections of synthetic reports, (ii) they yield a compact and interpretable token vocabulary that aligns with how chemists describe reaction conditions, and (iii) they facilitate direct comparison with prior condition-prediction studies. To mitigate inconsistencies in the literature, all reagent names were normalized via a curated synonym dictionary prior to tokenization. Although this representation may be less precise than SMILES and could limit extrapolation to rare or previously unseen reagents, our benchmarking indicates that generalization was not significantly impaired. Future work may benefit from integrating SMILES or hybrid encodings that combine structural fingerprints with common names to further improve robustness.

### Photocatalytic synthesis planning with PhotoCat

**Fig. 10:**
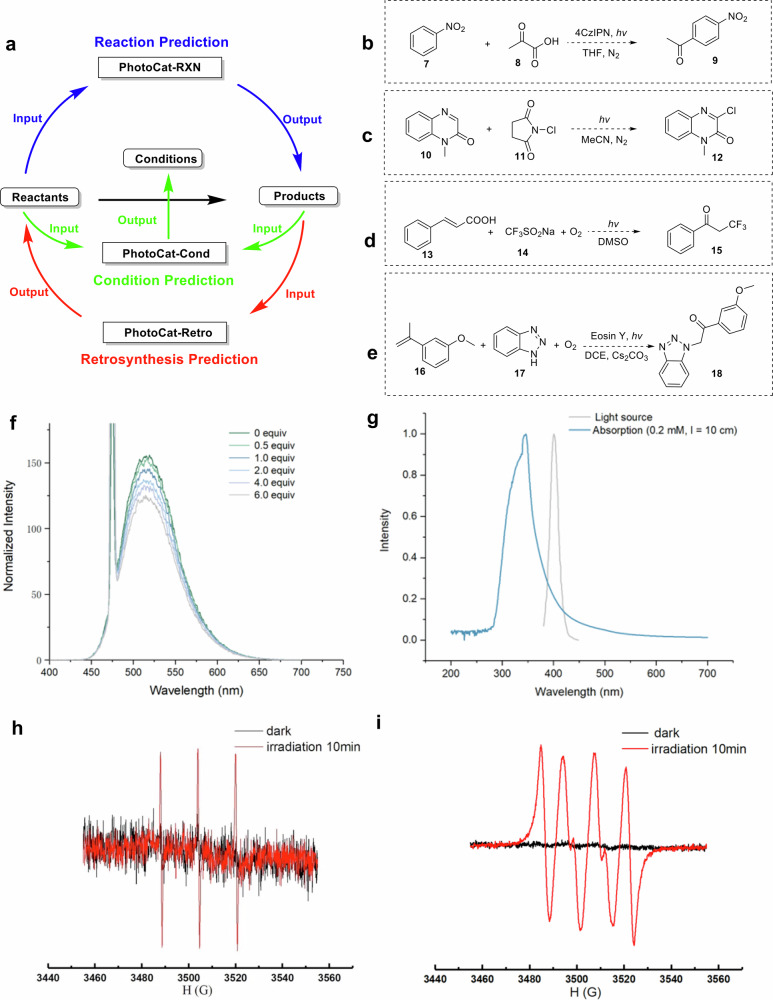
Photocatalytic synthesis planning and experimental validation guided by the PhotoCat. **a** Workflow of dry-lab (in silico) reaction planning using PhotoCat, integrating retrosynthesis, condition recommendation, and reaction prediction. **b**–**e** Four photocatalytic reactions proposed by PhotoCat and successfully validated through wet-lab experiments. **f**–**i** Mechanistic investigations supporting light-induced radical pathways.

PhotoCat provides valuable guidance for conducting wet-lab experiments through dry-lab (in silico) simulations. In this section, we demonstrate how PhotoCat assists in the synthetic planning of photocatalytic reactions (Fig. 10a). The first step is to use PhotoCat-Retro to perform retrosynthetic analysis of the target compound based on photocatalytic reaction rules, generating the corresponding reaction starting reactants. The second step involves using PhotoCat-Cond, where the reaction equation composed of the starting materials and reaction products is input to analyze possible reaction conditions, including photocatalysts, acids or bases, additives, solvents, and wavelengths. The third step is to use PhotoCat-RXN, inputting the reactants and conditions for reaction prediction. To demonstrate the practical utility of PhotoCat, we experimentally validated its Top-1 reaction predictions. This in silico reaction analysis quickly validates wet-lab experimental plans to accelerate the development of photocatalytic reactions and reduce trial-and-error costs. To ensure transparency, we summarize the overall prediction-validation workflow in Supplementary Table 27. PhotoCat-Retro generated 22 reaction candidates, of which 17 were excluded because they closely resembled reported literature reactions (Supplementary Table 28). The remaining 5 predictions, judged both novel and chemically feasible, were experimentally tested, resulting in 4 successful and 1 unsuccessful outcome, with full details provided in Supplementary Table 29. Detailed selection criteria and novelty assessment procedures are described in the “Methods” section.

With the assistance of PhotoCat, our team designed four novel photocatalytic reactions (Fig. 10b–e) in two hours, which had not been reported before. Subsequent wet-lab experiments confirmed that, under the recommended reaction conditions, reactions **b**–**e** produced the expected products efficiently. Control experiments (Supplementary Fig. 13), luminescence quenching screening studies, and electron paramagnetic resonance (EPR) analyses (Fig. 10f–i) established that these reactions proceed via a photo-induced radical initiation mechanism. Detailed comparisons of reactions **b**–**e** with the most closely related literature precedents are provided in Supplementary Table 30, clearly highlighting the novel features of our strategy over existing methods. Complete experimental procedures and mechanistic studies are described in Part 3 of the ESI.

In reaction **b**, pyruvate **7** and nitrobenzene **8** combine to produce aromatic ketone **9** under photocatalytic conditions. This reaction is reminiscent of the Friedel–Crafts acylation reaction. The introduction of strong electron-withdrawing groups, like the nitro group, desensitizes the benzene ring, making the execution of the Friedel–Crafts acylation reaction more challenging^73^. A notable advantage of Reaction **b** is that it obviates the need for the large amounts of Lewis acids (e.g., AlCl₃) typically required in traditional Friedel–Crafts acylations. Luminescence quenching screening studies (Fig. 10f) confirmed that Reaction **b** proceeds via a photocatalyzed radical pathway.

In reaction **c**, 2-aminophenol **10** and benzaldehyde **11** undergo a mild, catalyst-free photocatalytic transformation to afford 2-phenylbenzoxazole **12**. While recent reports have achieved the synthesis of product 12 through more complex strategies, such as using atomically dispersed Co/N-doped carbon catalysts obtained via high-temperature pyrolysis of ZnCo-ZIF precursors^74^, or employing NaCN as a catalyst^75^ with significant environmental hazards—our approach proceeds under ambient conditions without additional catalysts. This simplicity underscores the novelty of reaction **c**, providing a greener and more straightforward alternative that expands the chemist’s synthetic toolbox.

In reaction **d**, the photocatalytic synthesis of α-trifluoromethyl-substituted ketone **13** is achieved using cinnamic acid **14** and CF_3_SO_2_Na **15**. Compared with recently reported synthetic methods that employ AgNO₃ and K₂S₂O₈ as a catalytic oxidation system^76^, this approach requires only air as the oxidant and avoids the necessity of metals, external oxidants, or photocatalysts. The UV–visible absorption spectra (Fig. 10j) reveal an overlap between the absorption spectrum of cinnamic acid and the emission wavelength, indicating that compound **14** can absorb these wavelengths and form excited-state species. EPR trapping experiments using TEMP (Fig. 10h) and DMPO (Fig. 10i) confirmed the presence of singlet oxygen (^1^O_2_) and superoxide radical (O_2_^•−^) in the reaction under purple LED irradiation. To the best of our knowledge, this represents the inaugural documentation of an oxidative decarboxylative trifluoromethylation of α,β-unsaturated carboxylic acid employing cost-effective CF_3_SO_2_Na via any photocatalytic approach^77^.

In reaction **e**, a photo-triggered oxo-amination of an inactivated alkene **16** is developed, leading to the synthesis of α-amino ketones **18**^78^. This synthetic strategy resembles the recently reported photocatalytic method by the Shao group^79^, but utilizes the more economical eosin Y as the photocatalyst. It highlights a vicinal heterodifunctionalization of readily available olefin feedstocks, enabling the one-step construction of the target product.

## Conclusions

This study introduces PhotoCat, a deep learning platform based on Transformer architecture, designed to advance photocatalytic reaction research through artificial intelligence. We created the first specialized photocatalytic reaction database, PhotoCatDB, comprising 26,700 curated entries classified by reaction mechanisms. Using PhotoCatDB, we developed three Transformer-based modules: PhotoCat-RXN for reaction prediction, PhotoCat-Retro for retrosynthesis, and PhotoCat-Cond for reaction condition recommendation. These modules achieved top-1 accuracies of 82.3%, 69.4%, and 88.5%, respectively, demonstrating performance comparable to state-of-the-art models. Four practical photocatalytic pathways were designed and validated through wet-lab experiments, with mechanistic studies confirming that all reactions proceed through a photo-induced radical mechanism.

A significant additional finding is that classification and simplification of reaction conditions can markedly enhance the prediction accuracy of deep learning models for chemical reaction tasks. This approach addresses the common challenge that the inclusion of complex reaction conditions often reduces predictive performance. Furthermore, attention weight analysis reveals that this strategy improves model interpretability, providing insights into how reaction conditions affect prediction accuracy.

Overall, PhotoCat is a powerful tool for chemists developing photocatalytic reactions and has the potential to accelerate the broader adoption of photocatalysis as a green synthesis technology. This study also offers valuable guidance for researchers involved in scientific database construction. Future work will focus on expanding PhotoCatDB to better capture the complexity of photocatalytic reactions and further enhance PhotoCat’s utility in experimental research. In parallel, integrating PhotoCat with general synthetic planning tools will ensure that photocatalysis is proposed only when truly beneficial, thereby improving its practical value for end users.

## Methods

### PhotoCatDB

The PhotoCatDB is developed from a comprehensive review of photocatalytic reactions and our group’s expertise on photocatalytic reactions^80–85^. This database exclusively includes records from peer-reviewed published literature, excluding any pre-print versions or papers assessed as subjectively unreasonable. A team of 15 data collectors extracted and analyzed reactions from diverse sources. By analyzing the mechanisms, they organized the information into three main categories: reaction equations (expressed using SMILES; standardization of all reactions was performed using RDKit^86^), reaction conditions, and additional details. After cross-checking, the collected data was incorporated into PhotoCatDB. Reaction conditions were represented by common names mapped to unique token IDs rather than full SMILES, improving readability and reducing token complexity. Photocatalysts often contain large conjugated structures, making SMILES strings long and impractical for modeling. Similar simplification strategies have been used previously^28,42^. While common names are less standardized, we mitigate this by unique ID mapping, SMILES cross-validation, and leveraging the diversity of PhotoCatDB. A current limitation of PhotoCatDB is the lack of fine-grained reaction class annotations, which prevents us from systematically evaluating prediction performance by reaction type. Future expansions of PhotoCatDB will address this by incorporating more detailed labels, thereby enabling such analyses.

### TMAP generation

TMAP^87^ is a dimensionality reduction algorithm designed to handle millions of data points. Its advantage over other dimensionality reduction approaches lies in its two-dimensional tree-like output, which preserves both local and global structures, with an emphasis on local neighborhoods. The algorithm consists of four key steps: (1) LSH Forest-based indexing, (2) *k*-nearest-neighbor graph generation, (3) minimum spanning tree construction using Kruskal’s algorithm, and (4) creation of a tree-like layout. The resulting visualization is rendered using the interactive framework Faerun^88^. Following Schwaller et al.^52^, we generated reaction fingerprints using rxnfp (https://github.com/rxn4chemistry/rxnfp) and employed the default parameters for LSH Forest indexing, *k*-nearest-neighbor graph construction, and minimum spanning tree generation, which are sufficient to reveal the global structure of the dataset distribution. To improve interpretability in large datasets, we further adjusted node size and inter-cluster spacing during layout rendering. Labels and clustering parameters used in this study are publicly available at https://github.com/su-group/PhotoCat/tree/main/cluster_tmap, ensuring full transparency and reproducibility.

### PhotoCat-RXN

PhotoCat-RXN employs the Transformer model developed by Schwaller et al.^24^. The model was implemented using PyTorch as the backend deep learning framework. Both the encoder and decoder consisted of six layers. The word vectors and the recurrent neural network (RNN) had a dimensionality of 512. Gradient accumulation was performed eight times with a maximum vector norm of 0.0. The optimization process utilized the Adam optimizer with *β*₁ set to 0.9 and *β*₂ set to 0.998. The batch size was 4096, with batch type and gradient normalization method based on tokens. The learning rate was set to 2.0, and the decay method followed the Noam scheme. A dropout rate of 0.1 was applied, along with label smoothing (*ε*) set to 0.1. Parameter initialization was disabled, while position encoding was enabled.

### PhotoCat-Retro

PhotoCat-Retro employs Chemformer^65^, a state-of-the-art model specifically designed for chemical reaction retrosynthesis. Training was conducted for 100 epochs with a learning rate of 0.001, following a cyclical learning rate schedule. A batch size of 64 was used, with gradient accumulation over four batches. The retrosynthesis model training process involved three distinct stages. Initially, molecule-level pretraining was carried out on the ZINC-15^66^ dataset, containing 100 million molecules. During this phase, SMILES strings were subjected to span-masking, wherein contiguous short sequences were stochastically replaced with a “<MASK>“ token, thereby facilitating a deeper model comprehension of atomic connectivity and bonding patterns. Next, the pretrained molecular model underwent reaction-level pretraining using the USPTO dataset, comprising approximately 1 million chemical reactions. This step aimed to equip the model with the ability to recognize and learn diverse chemical reaction patterns. Finally, the model was fine-tuned specifically for photocatalytic reaction retrosynthesis tasks using the PhotoCatDB dataset, resulting in the specialized PhotoCat-Retro model.

### PhotoCat-Cond

PhotoCat-Cond is a Transformer-based deep learning model derived from Parrot^70^. The model is specifically optimized for predicting reaction conditions in photocatalytic reactions, aiming to assist chemists in selecting optimal parameters. PhotoCat-Cond is trained on PhotoCatDB-Cond, a curated subset designed for this task, and performs simultaneous classification of photocatalysts, solvents, and reagents in a single training process, improving both efficiency and accuracy. It is fine-tuned for two epochs using a small learning rate and a 5-fold data-augmented training set.

### Transfer learning and data augmentation

Transfer learning is a technique that leverages pre-trained models on a large dataset to improve performance on a related but typically smaller dataset. By transferring knowledge from a broader domain, models can achieve better generalization, especially when training data is limited. In our research, we utilized the USPTO dataset, originally derived from Lowe’s dataset^89^, which consists of data extracted from patents filed in the United States Patent and Trademark Office. We preprocessed this dataset by excluding reagents, solvents, temperature, and other reaction conditions, and subsequently filtered to eliminate duplicate, incorrect, and incomplete reactions. In this study, transfer learning was applied using a convex weighting scheme, where the USPTO dataset and the fine-tuning dataset were assigned weights of 9 and 1, respectively, following the approach described by Pesciullesi^43^. This strategy enables the model to retain broad chemical knowledge from USPTO while adapting to the specific characteristics of the fine-tuning dataset. Additionally, in PhotoCat-Cond, we incorporated SMILES-based data augmentation to enhance the accuracy of reaction condition predictions. Data augmentation was applied using multiple SMILES-based strategies. Specifically, we employed (i) randomized SMILES generation by altering atom order, (ii) canonical and non-canonical SMILES expansion, and (iii) span masking of subsequences with a <MASK> token. These augmentations, applied with a probability of 0.5 per sample, encourage the model to generalize across equivalent molecular encodings and mitigate overfitting. By generating augmented representations of molecular structures, this technique helps the model generalize better across diverse reaction conditions, ultimately improving its predictive performance.

### Cross-validation

Cross-validation, a widely adopted machine learning evaluation technique, assesses a model’s performance and generalization ability by dividing the dataset into exclusive subsets for training and validation. It effectively tackles overfitting and underfitting concerns while offering a comprehensive understanding of the model’s real-world performance. In this paper, all models were constructed and evaluated using 5-fold cross-validation, ensuring the robustness of the results.

### Reaction design selection and novelty assessment

For each target transformation, the integrated PhotoCat workflow (*PhotoCat-Retr*, *PhotoCat-Cond*, *PhotoCat-RXN*) was applied to generate candidate reactions. In terms of data format, taking PhotoCat-RXN as an example, the model inputs comprise reactant SMILES along with encoded condition labels (photocatalyst, solvent, base/acid, additives, and wavelength), while the output corresponds to the predicted product SMILES. Only the Top-1 prediction for each target was considered, provided that it employed readily available reagents under safe and practical conditions. For laboratory validation, concentrations and stoichiometries were guided by the molar ratios suggested by PhotoCat in combination with conventional practice for small-scale organic synthesis (0.2–0.9 mmol substrates in 2 mL solvent). Candidates overlapping with known literature or requiring rare reagents were excluded. Novelty was assessed using *Reaxys* and *SciFinder* searches with product structures, reaction types, and substrate scopes as queries. The proportion of novel reactions and the overall success rate were calculated as described in Table S26.

### Chemical synthesis

Unless otherwise specified, all reagents and solvents were obtained from commercial suppliers and used without further purification. The NMR spectra were recorded on a Bruker Avance 400 spectrometer at 400 MHz in CDCl_3_ with tetramethylsilane as the internal standard. Chemical shifts (*δ*) are reported in parts per million (ppm) and coupling constants (*J*) are reported in hertz (Hz). High-resolution mass spectra were obtained with a Bruker Impact II UHR-QTOF by electrospray ionization (ESI) on a time-of-flight (TOF) mass analyzer. Steady-state and time-resolved emission spectroscopy were conducted using an Edinburgh FLS1000. Column chromatography was performed on silica gel (200–300 mesh).

Reaction **b**: A mixture of nitrobenzene **7** (0.2 mmol), pyruvic acid **8** (0.4 mmol), and THF (2 mL) was added to a reaction tube. The tube was evacuated and backfilled with N_2_ three times. The mixture was then irradiated by 360–365 nm for 24 h. After completion of the reaction, the resulting mixture was extracted with CH_2_Cl_2_, and the organic phase was then removed under vacuum. The residue was purified by column chromatography using a mixture of petroleum ether and ethyl acetate as eluent to give the desired product **9** with 70.5% yield.

Reaction **c**: A mixture of 2-aminophenol **10** (0.2 mmol), benzaldehyde **11** (0.4 mmol), and DCM (2 mL) was added to a reaction tube. The reaction mixture was open to the air and stirred at room temperature under the irradiation of a 390 nm LED lamp for 48 h. After completion of the reaction, the resulting mixture was extracted with CH_2_Cl_2_, and the organic phase was then removed under vacuum. The residue was purified by column chromatography using a mixture of petroleum ether and ethyl acetate as eluent to give the desired product **12** with 71.1% yield.

Reaction **d**: A mixture of cinnamic acid **13** (0.2 mmol), CF_3_SO_2_Na **14** (0.4 mmol), and DMSO (2 mL) was added to a reaction tube. The reaction mixture was opened to the air and stirred at room temperature under the irradiation of purple light for 5 h. After completion of the reaction, the resulting mixture was extracted with CH_2_Cl_2_, and the organic phase was then removed under vacuum. The residue was purified by column chromatography using a mixture of petroleum ether and ethyl acetate as eluent to give the desired product **15** with 75.3% yield.

Reaction **e**: In an oven-dried reaction tube equipped with a magnetic stirrer bar was charged with α-methylstyrene **16** (0.9 mmol), benzotriazole **17** (0.3 mmol), cesium carbonate (0.9 mmol), Eosin Y (3.0 mol%), and DCE (2.0 mL). The tube was then exposed to blue LED irradiation at room temperature under an O_2_ atmosphere with stirring for 36 h. After completion of the reaction, the resulting mixture was extracted with CH_2_Cl_2_, and the organic phase was then removed under vacuum. The residue was purified by column chromatography using a mixture of petroleum ether and ethyl acetate as eluent to give the desired product **18** with a yield of 63.7%.

## Supplementary information

Supplementary Information

## Data Availability

The PhotoCatDB and supplementary datasets used in this study are available at 10.6084/m9.figshare.24532918.

## Code availability

The source code for PhotoCat, along with the associated Python scripts for data preparation, is publicly available at https://github.com/su-group/PhotoCat and archived at 10.5281/zenodo.18149429.
