# Supplementary material for: An artificial intelligence-driven synthesis planning platform (PhotoCat) for photocatalysis
Source: Commun Chem. 2026 Jan 21;9:92. doi: 10.1038/s42004-026-01894-y (PMC12913628; doi:10.1038/s42004-026-01894-y)
Supplement: Supplementary file 1 — Supplementary Information [file 42004_2026_1894_MOESM1_ESM.pdf]

Supporting Information

An artificial intelligence-driven synthesis planning platform (PhotoCat) for photocatalysis

Jiangcheng Xu,[#,1,2], Silong Zhai[#,1,3], Panyi Huang[#,1,4], Wenbo Yu[2], Qingyi Mao[1], Kui Du[5], Weike Su[1], Bin Sun[1,*], Can Jin[1,6,*], An Su[6,*]

1. National Engineering Research Center for Process Development of Active Pharmaceutical Ingredients, Collaborative Innovation Center of Yangtze River Delta Region Green Pharmaceuticals, Zhejiang University of Technology, Hangzhou, 310014, P. R. China
2. Hangzhou Polytechnic University, Hangzhou, 310014, P. R. China
3. Faculty of Applied Science, Macao Polytechnic University, Macau SAR, 999078, P. R. China
4. Institute of Advanced Studies and School of Pharmaceutical Sciences, Taizhou University, Jiaojiang, 318000, P. R. China
5. School of Chemistry and Chemical Engineering, Shaoxing University, Shaoxing 312000, P. R. China
6. Zhejiang Key Laboratory of Green Manufacturing Technology for Chemical Drugs, College of Pharmaceutical Sciences, Zhejiang University of Technology, Hangzhou, 310014, P. R. China

These authors contributed equally: Jiangcheng Xu, Silong Zhai, Panyi Huang.

***Corresponding authors:**
Prof. An Su
College of Pharmaceutical Sciences, Zhejiang University of Technology, Hangzhou, 310014, P. R. China
Email: ansu@zjut.edu.cn

Prof. Can Jin
College of Pharmaceutical Sciences, Zhejiang University of Technology, Hangzhou, 310014, P. R. China
E-mail: jincan@zjut.edu.cn

Prof. Bin Sun
Collaborative Innovation Center of Yangtze River Delta Region Green Pharmaceuticals, Zhejiang University of Technology, Hangzhou, 310014, P. R. China
E-mail: sunbin@zjut.edu.cn

Content

1. Details of PhotoCatD 1

1.1 Curation workflow and decisions 1

1.2 Classification of Reaction Conditions 2

1.3 Examples of data in PhotoCatDB. 3

1.4 Data distribution and composition analysis of PhotoCatDB-Cond 6

1.5 Visualizing PhotoCatDB: Bias, Diversity, and Limitations 7

1.6 Data overlap between USPTO and PhotoCatDB 10

1.7 Identification number for special reaction conditions (ligands) in PhotoCatDB 11

2. Test details 12

2.1 Comparison of test results between PhotoCat-RXN and Baseline models (5-fold cross-validation). 12

2.1.1 Baseline-1 model 12

2.1.2 Baseline-2 model 12

2.1.3 PhotoCat-RXN 13

2.1.4 Baseline-3 model 13

2.1.5 Pre-trained checkpoints and augmented root-aligned SMILES 13

2.2 Test results with varying numbers of reaction condition inputs 14

2.2.1 One reaction condition 14

2.2.2 Two reaction conditions 14

2.2.3 Three reaction conditions 15

2.2.4 Four reaction conditions 15

2.2.5 Five reaction conditions 16

2.3 The test results of PhotoCat-Retro 17

2.3.1 PhotoCat-Retro (PhotoCatDB) 17

2.3.2 PhotoCat-Retro(ZINC+PhotoCatDB) 17

2.3.3 PhotoCat-Retro(ZINC+USPTO+PhotoCatDB) 17

2.4 The test results of PhotoCat-Cond 18

2.4.1 The test results of photocatalyst recommendation. 18

2.4.2 The test results of base or acid recommendation. 18

2.4.3 The test results of wavelength recommendation. 18

2.4.4 The test results of additive recommendation. 19
2.4.5 The test results of solvent recommendation. 19
2.4.6 Comparison of reaction condition recommendation models 20
2.5 Graph2SMILES with Condition Encoding for Reaction Prediction 21
2.6 Reaction Conditions as Key Determinants: Case Studies 23
3. Discovery of new photocatalytic reactions. 25
3.1 Validation of the *PhotoCat-Retro* module 25
3.2 Statistical transparency and summary of experimental validation 26
3.3 The novelty of the four photocatalytic reactions 31
3.4 Chemistry synthesis 33
3.4.1 Reaction b 33
3.4.2 Reaction c 33
3.4.3 Reaction d 34
3.4.4 Reaction e 34
3.4.5 reaction 5 (Table S29) 35
3.5 Mechanism investigation 36
3.5.1. Control experiment 36
3.5.2 Luminescence Quenching Screening Studies (Reaction b) 37
3.5.3 UV-visible absorption Spectra (Reaction d) 37
3.5.4 EPR experiment (Reaction d) 38
3.6 Proposed mechanism 40
3.7 Characterization data for the products 44
3.8 ^{1}H, ^{13}C and ^{19}F NMR spectra of products 46
4. References 51

1. Details of PhotoCatD

1.1 Curation workflow and decisions

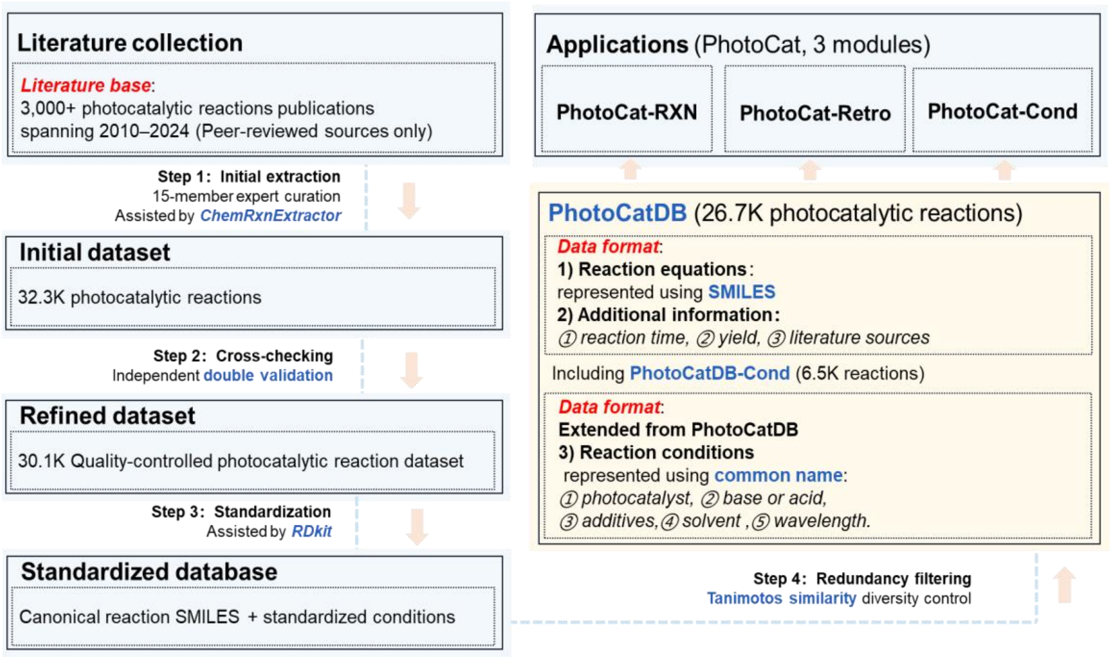

Supplementary Figure 1. Workflow for constructing PhotoCatDB. Over 3,000 peer-reviewed photocatalysis papers (2010–2024) were used to extract an initial dataset of 32.3K reactions. After manual validation and quality control, 30.1K reactions were retained. The data were standardized into canonical reaction SMILES and unified condition names, and redundancy was reduced using Tanimoto similarity. The final standardized database, PhotoCatDB, contains 26.7K curated reactions, including a condition-rich subset (PhotoCatDB-Cond, 6.5K reactions), which supports three downstream modules: PhotoCat-RXN, PhotoCat-Retro, and PhotoCat-Cond.

1.2 Classification of Reaction Conditions

Supplementary Table 1. Definition and classification of photocatalytic reaction conditions

Category	Definition	Examples	Notes
**Photocatalyst**	Substances that can absorb photons and reach an excited state, thereby initiating or promoting electron transfer/energy transfer processes.	Transition-metal complexes ($Ru(bpy)_3^{2+}$, $Ir(ppy)_3$), organic dyes (Eosin Y, Rose Bengal), semiconductor materials	In some cases, substrates themselves act as photosensitizers (autocatalysis).
**Base or Acid**	Substances that adjust the acidity/basicity of the reaction medium or participate in proton/electron transfer processes, thereby influencing the stability of radicals or intermediates.	Bases: NEt_3, Cs_2CO_3; Acids: TfOH, acetic acid.	In photochemical systems, bases such as NEt_3 are classified as "Base or Acid" even when serving as sacrificial electron donors.
**Additives**	Components other than photocatalysts and acids/bases that improve reaction efficiency, selectivity, or stability.	Ligands (e.g., bipyridines), electron-transfer mediators (e.g., TEMPO), iodine salts.	Limited to chemicals not classified as acids or bases.
**Solvent**	Liquids that dissolve substrates and reagents, provide a homogeneous medium, and potentially influence reaction mechanisms and yields through polarity, hydrogen bonding, or energy transfer properties.	DCM, DMSO, MeCN, THF.	—
**Wavelength**	The wavelength range of light used to irradiate the reaction mixture, which directly determines the excitation of the photocatalyst or substrates.	Violet (λ = 380–420 nm), Blue (λ = 400–450 nm), Green (λ = 500–540 nm), Red (λ = 600–650 nm), UV lamp (λ = 350–390 nm), White light source (broad spectrum).	The wavelength ranges included in this study were collected from reported literature and experimental data, and are presented here solely as classification criteria rather than prescriptive experimental guidance.

*Classification priority: In cases where a reagent has multiple possible roles, it is classified according to the priority order Photocatalyst > Base or Acid > Additives > Solvent.

1.3 Examples of data in PhotoCatDB.

Supplementary Table 2. Example 1. Combining photoredox-catalyzed trifluoromethylation and oxidation with DMSO: facile synthesis of α-trifluoromethylated ketones from aromatic alkenes

**Entry**	142	**Category**	three-component photocatalysis
**Reaction**	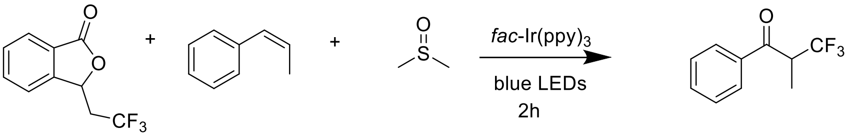		
**SMILES for reaction equations**	O=C1OC(CC(F)(F)F)C2=CC=CC=C21.C/C=C\C3=CC=CC=C3.O=S(C)C>>O=C(C(C)C(F)(F)F)C4=CC=CC=C4		
**Note**	DMSO acts both as a solvent and as a reactant to provide oxygen atoms. In the corresponding *Reaxys dataset* (*Reaction ID: 39151038*), this reactant is missing.		

**Reaction condition**	***Photocatalyst***	***Acid or base***	***Additive***	***Solvent***
	fac-Ir(ppy)3	none	none	DMSO
	Light source	***Light categories***	***Wavelength***	***Power***
	LED	Blue	425nm	-
**Additional information**	***Reaction time(h)***	***Yield(%)***	***Literature sources***	
	2	75	*Angew. Chem., Int. Ed.*, **2014**, *53*, 7144–7148.	

Supplementary Table 3. Example 2. Metal-free di- and tri-fluoromethylation of alkenes realized by visible-light-induced perylene photoredox catalysis.

**Entry**	1072	**Category**	four-component photocatalysis
**Reaction**	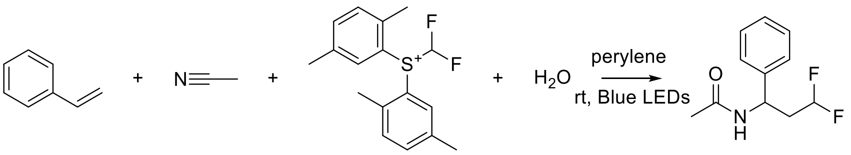		
**SMILES for reaction equations**	C=CC1=CC=CC=C1.CC#N.FC(F)[S+](C2=CC(C)=CC=C2C)C3=C(C=CC(C)=C3)C.O>>O=C(C)NC(C4=CC=CC=C4)CC(F)F		
**Note**	Acetonitrile and water are involved in the final step of the reaction (Ritter-amination). In the corresponding *Reaxys dataset (Reaction ID: 46082493)*, water as a reactant is missing.		

**Reaction condition**	***Photocatalyst***	***Acid or base***	***Additive***	***Solvent***
	Perylene	none	none	MeCN / H_2O
	Light source	***Light categories***	***Wavelength***	***Power***
	LED	Blue	425nm	-
**Additional information**	***Reaction time(h)***	***Yield(%)***	***Literature sources***	
	6	76	*Chem. Sci.*, **2017**, *8*, 6375–6379.	

Supplementary Table 4. Example 3. Visible-light-driven photoredox-catalyzed three-component radical cyanoalkylfluorination of alkenes with oxime esters and a fluoride ion

**Entry**	2117	**Category**	three-component photocatalysis

Reaction

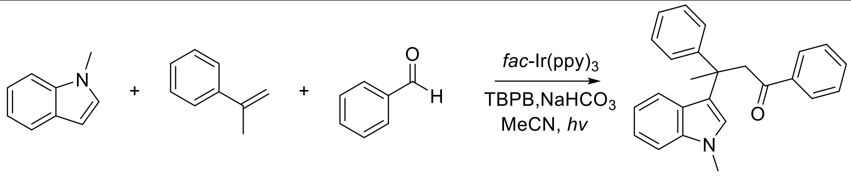

SMILES for reaction equations

CN1C=CC2=CC=CC=C21.CC(C3=CC=CC=C3)=C.[H]C(C4=CC=CC=C4)=O>>CN5C=C(C6=C5C=CC=C6)C(C7=CC=CC=C7)CCC(C8=CC=CC=C8)=O

Note

Tert-butyl perbenzoate (TBPB), the oxyl radical precursor, which could be reductively cleaved by ET from a photocatalyst, which would in turn oxidize the radical intermediate.

**Reaction condition**	***Photocatalyst***	***Acid or base***	***Additive***	***Solvent***
	fac-Ir(ppy)3	$NaHCO_3$	TBPB	MeCN
	Light source	***Light categories***	***Wavelength***	***Power***
	LED	Blue	425nm	-
**Additional information**	***Reaction time(h)***	***Yield(%)***	***Literature sources***	
	12	68	*Org. Lett.*, **2021**, *23*, 6987–6992.	

Supplementary Table 5. Example 4. Combining photoredox and silver catalysis for azidotrifluoromethoxylation of styrenes.

**Entry**	3005	**Category**	three-component photocatalysis

Reaction

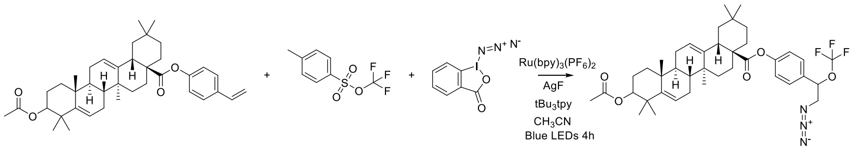

SMILES for reaction equations

O=C([C@@]12CC[C@]3([C@@]4(CC=C5C(C)(C(CC[C@@]5([C@]4(CC=C3[C@@]1(CC(C)(CC2)C)[H])[H])C)OC(C)=O)C)[H])C)OC6=CC=C(C=C6)C=C.CC7=CC=C(C=C7)S(=O)(OC(F)(F)F)=O.O=C8OI(N=[N+]=[N-])C9=C8C=CC=C9>[Ag]F.CC#N>O=C([C@@]%10%11CC[C@]%12([C@@]%13(CC=C%14C(C)(C(CC[C@@]%14([C@]%13(CC=C%12[C@@]%10(CC(C)(CC%11)C)[H])[H])C)OC(C)=O)C)[H])C)OC%15=CC=C(C=C%15)C(CN=[N+]=[N-])OC(F)(F)F

Note

The chemical reactions associated with natural drugs are important for training deep learning models. This photocatalytic reaction is not included in the *Reaxys* dataset.

**Reaction condition**	***Photocatalyst***	***Acid or base***	***Additive***	***Solvent***
	Ru(bpy)3(PF6)2	none	AgF, tBu3tpy	MeCN
	Light source	***Light categories***	***Wavelength***	***Power***
	LED	Blue	425nm	-
**Additional information**	***Reaction time(h)***	***Yield(%)***	***Literature sources***	
	4	38	*Chem. Commun.*, **2018**, *54*, 4473–4476.	

Supplementary Table 6. Example 5. Eosin Y catalyzed difunctionalization of styrenes using O2 and CS2: a direct access to 1,3-oxathiolane-2-thiones.

**Entry**	4027	**Category**	three-component photocatalysis

Reaction

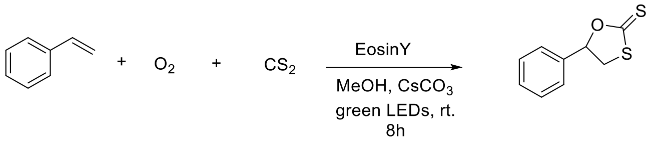

SMILES for reaction equations

C=CC1=CC=CC=C1.O=O.S=C=S>>S=C(O2)SCC2C3=CC=CC=C3

Note

In this paper the authors suggest that the oxygen in the product molecule comes from oxygen in air, whereas in the *Reaxys dataset (Reaction ID: 42975515)* it is thought to come from the solvent methanol.

**Reaction condition**	***Photocatalyst***	***Acid or base***	***Additive***	***Solvent***
	Eosin Y	$CsCO_3$	none	MeOH
	Light source	***Light categories***	***Wavelength***	***Power***
	LED	Green	500nm	5W
**Additional information**	***Reaction time(h)***	***Yield(%)***	***Literature sources***	
	8	92	*Green Chem.*, **2016**, *18*, 4240–4244.	

Supplementary Table 7. Example 6. Alkoxyl radicals generated under photoredox catalysis: a strategy for anti-markovnikov alkoxylation reactions

**Entry**	4229	**Category**	three-component photocatalysis

Reaction

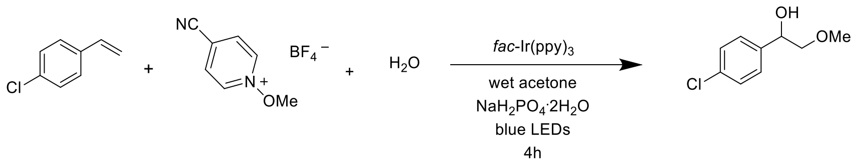

SMILES for reaction equations

C=CC1=CC=C(Cl)C=C1.N#CC2=CC=[N+](OC)C=C2.FB(F)F.[F-].O>>ClC3=CC=C(C(O)COC)C=C3

Note

In this reaction, the water as a reactant is hidden in the word 'wet', which was missed by the *Reaxys dataset (Reaction ID: 49168884)*. The *PhotoCatDB* avoids similar omissions by reviewing the literature and understanding the mechanism.

**Reaction condition**	***Photocatalyst***	***Acid or base***	***Additive***	***Solvent***
	fac-Ir(ppy)3	NaH_2PO_4	none	acetone
	Light source	***Light categories***	***Wavelength***	***Power***
	LED	Blue	425nm	-
**Additional information**	***Reaction time(h)***	***Yield(%)***	***Literature sources***	
	4	65	*Angew. Chem., Int. Ed.*, **2018**, *57*, 13790–13794.	

1.4 Data distribution and composition analysis of PhotoCatDB-Cond

The PhotoCatDB-Cond is based on a review of multicomponent photocatalytic reactions by Basso[1] and Sharma[2] and our group's experience in photocatalytic reactions[3-6]. To address the problem of over-aggregation of reactions in the same class, we grouped the literature during the literature collection following the classification of multi-component photocatalytic reactions. Analysis indicates that the data in PhotoCatDB-Cond is evenly distributed (Supplementary Figure 2).

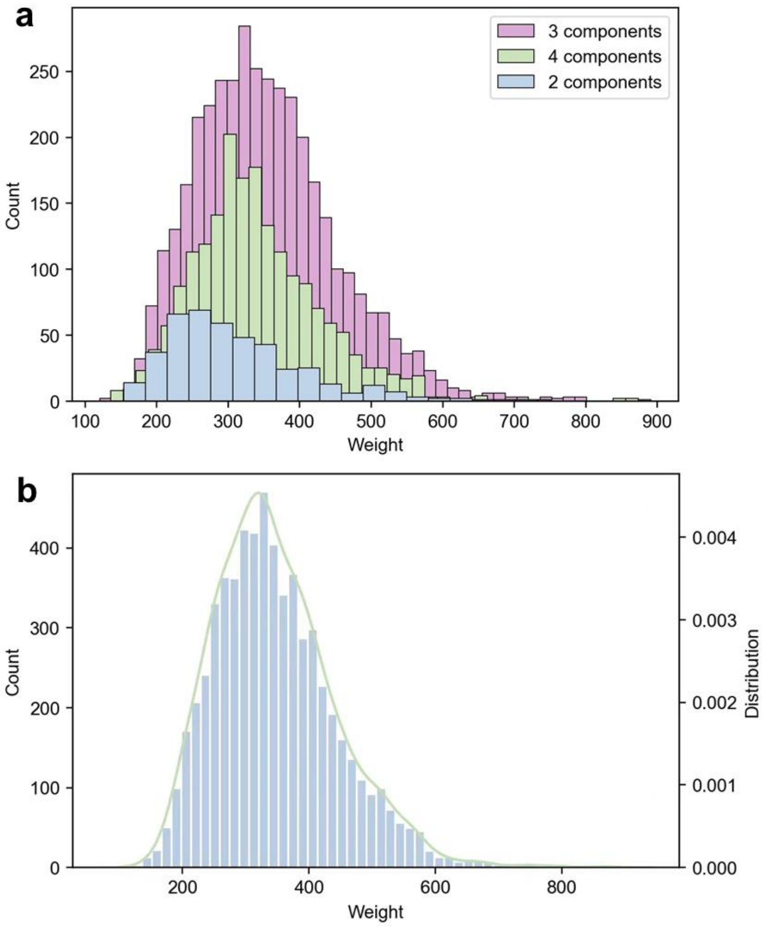

Supplementary Figure 2. Data distribution and composition analysis of PhotoCatDB-Cond. (a) Multicomponent reactions dominate the dataset, with three- and four-component reactions highlighted in pink and green, respectively. (b) The molecular weights of reaction products follow a normal distribution.

1.5 Visualizing PhotoCatDB: Bias, Diversity, and Limitations

While PhotoCatDB was curated with careful quality control and standardization, it inevitably reflects certain biases inherent to the published literature. Widely studied photocatalysts and frequently reported transformations are disproportionately represented, whereas less conventional systems and negative results are rarely included. This introduces a degree of dataset bias toward popular reaction classes and conditions.

To improve diversity, we applied redundancy filtering based on Tanimoto similarity and standardized all entries into canonical reaction SMILES and unified condition names. Our chemical space analysis shows that the curated dataset spans a broad range of scaffolds and reaction conditions, though certain regions remain relatively sparse, reflecting gaps in the current literature. Importantly, when employing T-Map for the analysis of PhotoCatDB, we observed distinct clustering patterns when categorizing by different reaction conditions (**Supplementary Figure 3-5**). This indicates that photocatalytic reactions sharing the same conditions tend to exhibit similarities within the chemical domain, supporting both the diversity and structured organization of the database.

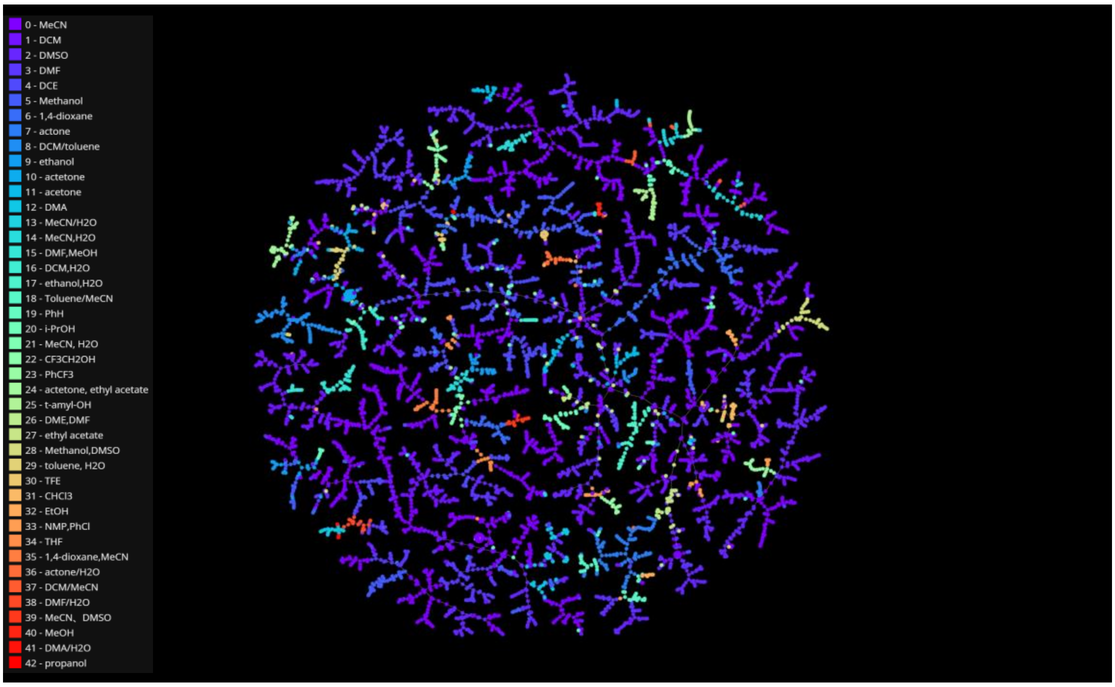

Supplementary Figure 3. Visualization of PhotoCatDB with Solvent as label.

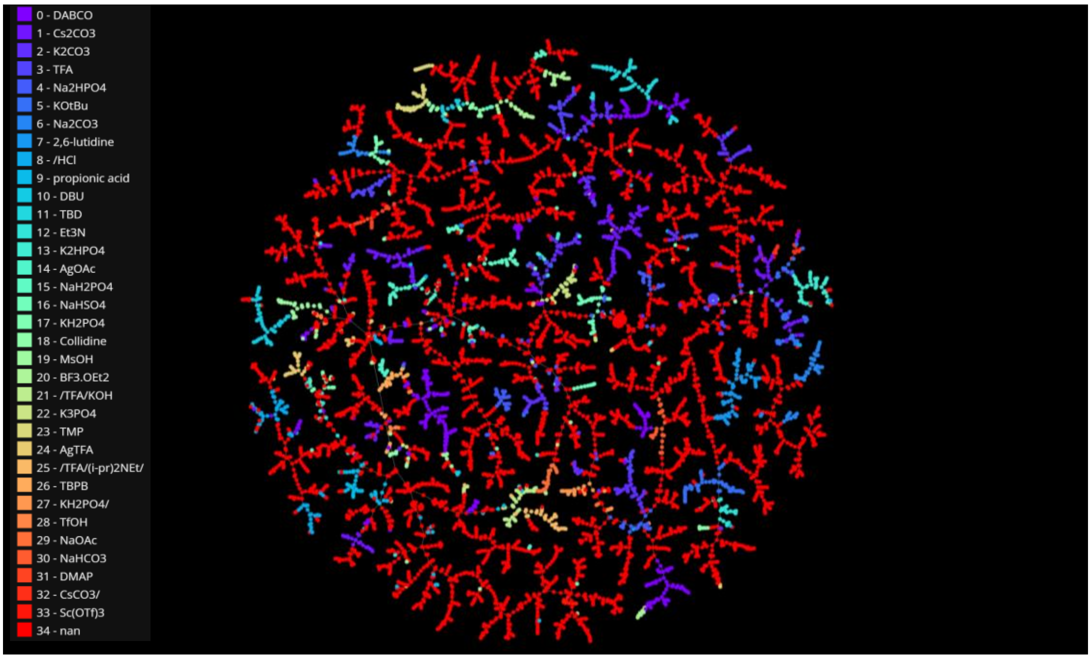

Supplementary Figure 4. Visualization of PhotoCatDB with Base or Acid as label.

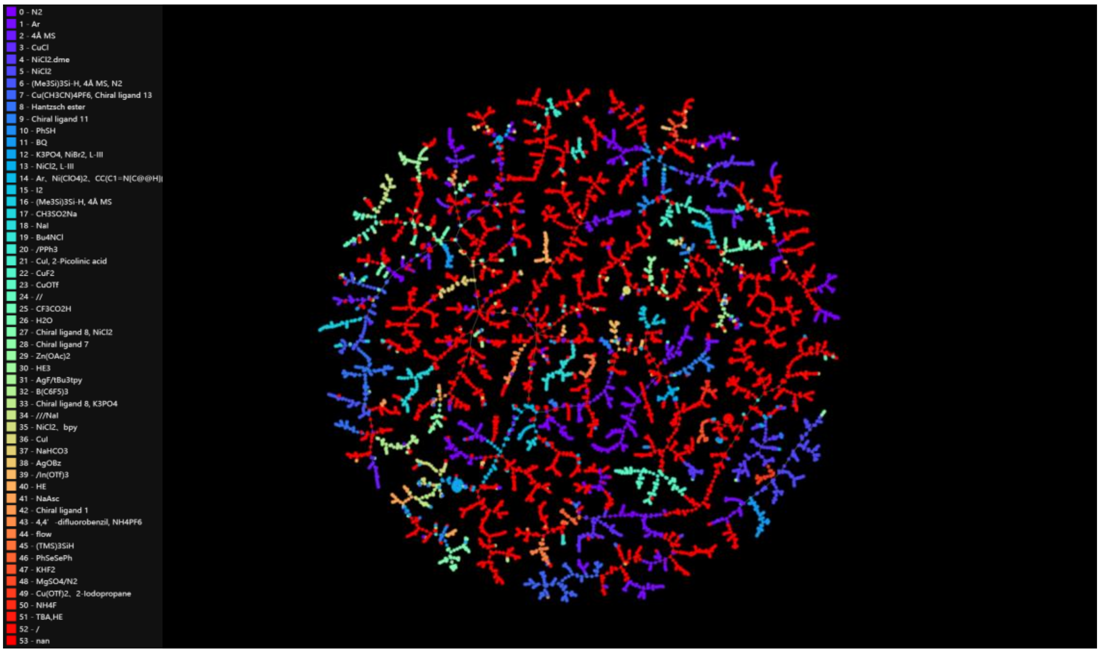

Supplementary Figure 5. Visualization of PhotoCatDB with Additive as label.

The products of the photocatalytic reactions of the different components in PhotoCatDB show an ideal normal Gaussian distribution in terms of molecular weight (**Supplementary Figure 6**).

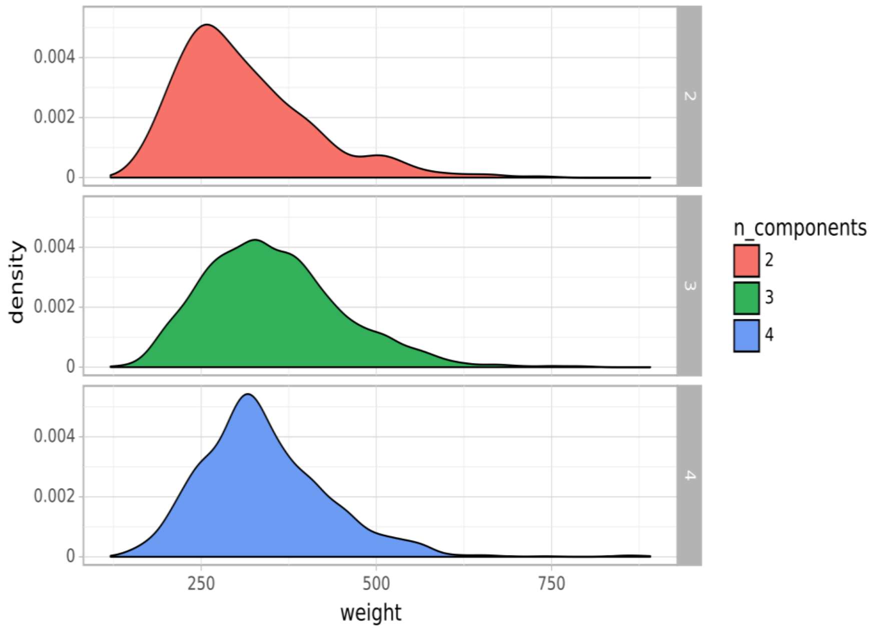

Supplementary Figure 6. The products of the photocatalytic reactions of the different components all show an ideal normal Gaussian distribution in terms of molecular mass.

Finally, we acknowledge several limitations. PhotoCatDB is built on information reported in peer-reviewed publications, which may omit unsuccessful or low-yielding experiments. Moreover, some condition descriptors remain simplified despite standardization, and mechanistic heterogeneity across reactions is not explicitly captured. To illustrate this diversity, TMAP analysis (**Fig. 3**) revealed distinct clustering of Ir- and Ru-based complexes, while organic dyes appeared more diffuse and partially overlapped with both metal-based clusters. Grouping by acid/base strength further showed that strongly basic additives clustered separately from neutral or mildly acidic conditions, indicating that condition choice is a major driver of reaction space organization. Notably, the distribution of photosensitizers in PhotoCatDB is non-uniform: Ir-based complexes dominate (42%), followed by Ru-based (33%) and organic dyes (25%). This pattern reflects the literature's bias toward particular catalyst classes and highlights opportunities to explore underrepresented regions of photocatalytic space. Therefore, PhotoCatDB should be viewed as a transparent, literature-based resource that improves accessibility to photocatalytic reaction data, while not representing an exhaustive or unbiased survey of the entire field.

1.6 Data overlap between USPTO and PhotoCatDB

During curation, we explicitly checked for potential redundancies between USPTO and PhotoCatDB, and no overlapping reactions were identified. USPTO primarily consists of patented synthetic reactions under conventional conditions, covering general organic reactions extracted from U.S. patents filed between 1976 and 2016. In contrast, PhotoCatDB was independently curated from peer-reviewed photocatalysis literature published between 2010 and 2024, and exclusively contains photocatalytic reactions. Given these distinct sources and time frames, the two datasets are non-overlapping and complementary. To further substantiate this, we added a T-Map visualization (**Fig. S7**), in which USPTO reactions are shown as blue points and PhotoCatDB reactions as red points. The two datasets form distinct and well-separated clusters with clear boundaries, providing direct evidence that there is virtually no overlap between them.

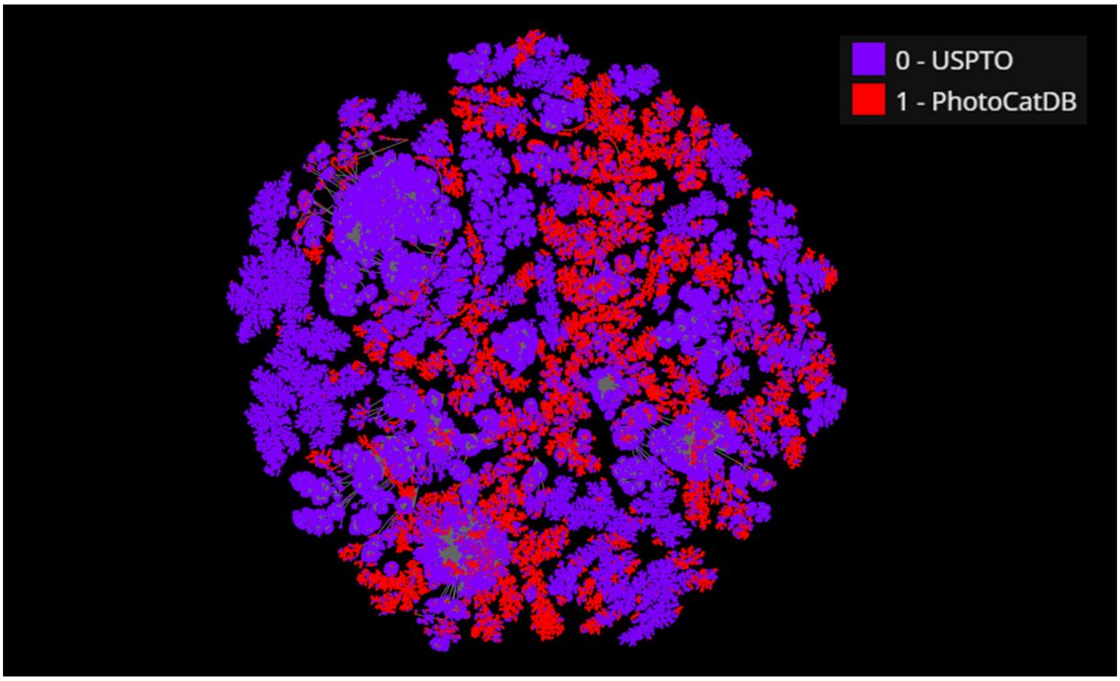

Supplementary Figure 7. T-Map visualization of USPTO and PhotoCatDB. USPTO reactions are shown as blue points, and PhotoCatDB reactions are shown as red points. The two datasets form distinct clusters with relatively clear boundaries, indicating that there is virtually no overlap between USPTO and PhotoCatDB. This visualization further confirms that the datasets are complementary.

1.7 Identification number for special reaction conditions (ligands) in PhotoCatDB

The numbers L1-L32 are used to simplify the representation of ligands (**Figure S7**). Common metal ligands, such as DPEPhos, BINAP, etc., are represented by common name.

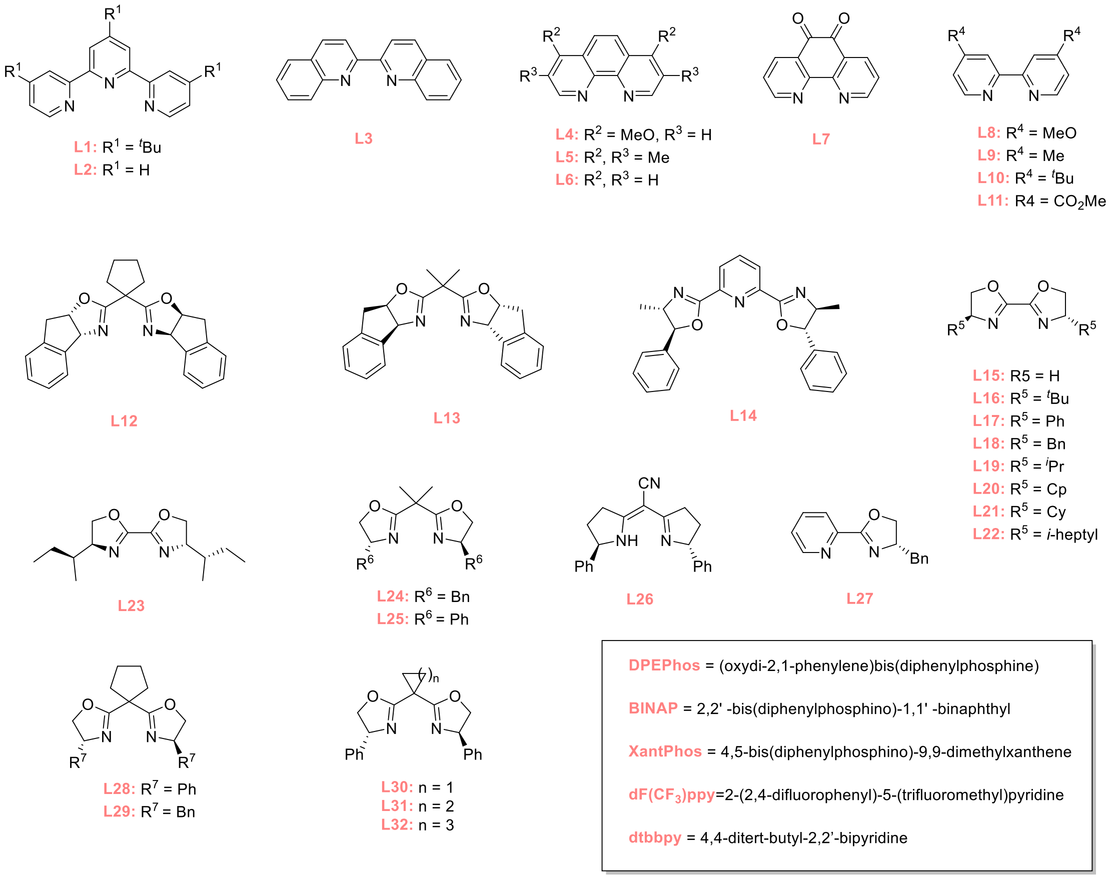

Supplementary Figure 8. ID for specific reaction conditions (ligands) in PhotoCatDB.

2. Test details

2.1 Comparison of test results between PhotoCat-RXN and Baseline models (5-fold cross-validation).

2.1.1 Baseline-1 model

Supplementary Table 8. Test detail for Baseline-1 model

**Experiment***	Top-1	Top-2	Top-3	Top-4	Top-5
1	1.17%	1.71%	2.61%	2.88%	3.15%
2	0.18%	0.27%	0.27%	0.63%	0.72%
3	0.27%	0.72%	0.90%	1.26%	1.35%
4	0.00%	0.45%	0.63%	0.81%	0.90%
5	0.72%	0.81%	0.81%	1.17%	1.17%
average	0.46%	0.79%	1.04%	1.35%	1.45%

*Steps = 50,000.

2.1.2 Baseline-2 model

Supplementary Table 9. Test detail for Baseline-2 model

**Experiment***	Top-1	Top-2	Top-3	Top-4	Top-5
1	21.62%	28.82%	32.97%	33.68%	35.29%
2	24.01%	32.85%	35.92%	40.54%	41.53%
3	26.21%	34.32%	37.38%	38.73%	41.08%
4	24.77%	42.70%	45.22%	46.93%	47.55%
5	25.40%	33.42%	38.37%	38.46%	39.54%
average	24.40%	34.42%	37.97%	39.66%	40.99%

*Steps = 50,000.

2.1.3 PhotoCat-RXN

Supplementary Table 10. Test detail for PhotoCat-RXN

**Experiment***	Top-1	Top-2	Top-3	Top-4	Top-5
1	69.55%	78.20%	80.81%	82.97%	84.14%
2	72.14%	78.72%	81.88%	83.86%	84.49%
3	70.69%	78.00%	81.06%	82.69%	83.77%
4	71.69%	78.81%	80.52%	82.15%	82.96%
5	69.34%	76.47%	78.99%	80.16%	81.61%
average	70.68%	78.04%	80.65%	82.37%	83.39%

*Steps = 50,000.

2.1.4 Baseline-3 model

Supplementary Table 11. Test detail for Baseline-3 model

**Experiment***	Top-1	Top-2	Top-3	Top-4	Top-5
1	61.14%	69.32%	72.88%	74.88%	76.15%
2	61.15%	70.13%	73.41%	75.56%	76.78%
3	59.05%	68.60%	72.44%	74.84%	76.65%
4	60.22%	69.78%	73.74%	75.46%	77.02%
5	62.88%	70.65%	73.59%	75.56%	76.93%
average	60.89%	69.70%	73.21%	75.26%	76.71%

* Steps = 50,000.

2.1.5 Pre-trained checkpoints and augmented root-aligned SMILES

Supplementary Table 12. Pre-trained checkpoints and augmented root-aligned SMILES

**Experiment***	Top-1	Top-2	Top-3	Top-4	Top-5
1	0.63%	1.35%	1.98%	2.07%	2.43%
2	0.90%	1.71%	2.25%	2.89%	3.06%
3	0.54%	1.17%	1.53%	1.89%	2.25%
4	0.63%	1.35%	1.98%	2.43%	2.43%
5	0.72%	1.26%	1.71%	2.25%	2.34%
average	0.69%	1.37%	1.89%	2.30%	2.51%

*Steps = 50,000.

2.2 Test results with varying numbers of reaction condition inputs

2.2.1 One reaction condition

Supplementary Table 13. Details of test results with the addition of one reaction condition (photocatalyst).

Experiment*	Top-1	Top-2	Top-3	Top-4	Top-5
1	79.73%	85.13%	87.21%	88.38%	89.19%
2	79.71%	86.29%	88.00%	88.36%	88.95%
3	78.72%	85.39%	86.93%	87.65%	88.46%
4	79.89%	85.75%	87.74%	88.82%	89.17%
5	81.61%	86.56%	88.19%	88.55%	88.82%
average	79.93%	85.82%	87.61%	88.35%	88.91%

*Steps = 50,000.

2.2.2 Two reaction conditions

Supplementary Table 14. Details of test results with the addition of two reaction condition (photocatalyst, Base or acid).

Experiment*	Top-1	Top-2	Top-3	Top-4	Top-5
1	80.09%	86.22%	87.84%	89.01%	89.55%
2	81.51%	85.84%	87.19%	88.64%	89.27%
3	80.16%	86.02%	87.37%	88.63%	89.18%
4	81.06%	86.03%	87.11%	88.55%	89.45%
5	82.24%	86.74%	88.28%	89.17%	89.90%
average	81.01%	86.17%	87.56%	88.80%	89.47%

*Steps = 50,000.

2.2.3 Three reaction conditions

Supplementary Table 15. Details of test results with the addition of three reaction condition (photocatalyst, Base or acid, additive).

**Experiment***	Top-1	Top-2	Top-3	Top-4	Top-5
1	80.54%	86.57%	88.65%	89.81%	90.36%
2	82.51%	86.65%	88.09%	89.09%	89.45%
3	82.15%	87.11%	88.10%	88.82%	89.27%
4	81.43%	85.48%	87.47%	88.46%	88.46%
5	82.24%	87.11%	88.28%	88.82%	88.91%
average	81.77%	86.58%	88.12%	89.00%	89.29%

*Steps = 50,000.

2.2.4 Four reaction conditions

Supplementary Table 16. Details of test results with the addition of three reaction condition (photocatalyst, Base or acid, additive, wavelength).

**Experiment***	Top-1	Top-2	Top-3	Top-4	Top-5
1	82.15%	87.01%	88.83%	88.93%	89.11%
2	81.75%	85.34%	88.49%	88.51%	88.69%
3	81.57%	87.52%	88.10%	88.82%	88.90%
4	82.87%	86.72%	89.01%	89.65%	89.91%
5	81.56%	86.78%	87.01%	89.71%	90.10%
average	81.98%	86.67%	88.29%	89.12%	89.34%

*Steps = 50,000.

2.2.5 Five reaction conditions

Supplementary Table 17. Details of test results with the addition of five reaction condition (photocatalyst, Base or acid, additive, solvent, wavelength).

**Experiment***	Top-1	Top-2	Top-3	Top-4	Top-5
1	82.26%	86.04%	87.48%	88.02%	88.56%
2	82.24%	87.65%	89.00%	90.00%	90.62%
3	81.70%	86.74%	88.81%	89.81%	90.00%
4	82.65%	87.38%	88.64%	89.36%	89.90%
5	82.41%	87.01%	88.81%	89.185	89.90%
average	82.25%	86.96%	88.55%	89.27%	89.80%

* Steps = 50,000.

2.3 The test results of PhotoCat-Retro

2.3.1 PhotoCat-Retro (PhotoCatDB)

Supplementary Table 18. The test results of PhotoCat-Retro (PhotoCatDB)

**Experiment***	Top-1	Top-3	Top-5	Top-10
1	16.57%	31.71%	40.00%	47.39%
2	15.33%	30.47%	36.34%	43.82%
3	16.86%	41.47%	38.14%	45.45%
4	17.22%	30.65%	38.32%	45.53%
5	17.04%	30.65%	37.06%	43.10%
average	16.60%	32.99%	37.97%	45.06%

2.3.2 PhotoCat-Retro(ZINC+PhotoCatDB)

Supplementary Table 19. The test results of PhotoCat-Retro (ZINC+PhotoCatDB)

**Experiment***	Top-1	Top-3	Top-5	Top-10
1	82.43%	90.18%	91.17%	91.98%
2	83.67%	91.79%	92.60%	93.15%
3	84.04%	90.89%	91.97%	92.43%
4	83.77%	89.99%	91.34%	92.25%
5	82.51%	90.17%	91.79%	92.33%
average	83.28%	90.60%	91.77%	92.42%

2.3.3 PhotoCat-Retro(ZINC+USPTO+PhotoCatDB)

Supplementary Table 20. The test results of PhotoCat-Retro (ZINC+USPTO+PhotoCatDB)

**Experiment***	Top-1	Top-3	Top-5	Top-10
1	84.59%	90.54%	90.99%	91.53%
2	83.86%	91.61%	92.06%	92.6%
3	84.31%	92.43%	93.05%	93.59%
4	85.84%	90.89%	91.97%	92.69%
5	83.58%	90.80%	91.34%	91.97%
average	84.43%	91.25%	91.88%	92.48%

2.4 The test results of PhotoCat-Cond

2.4.1 The test results of photocatalyst recommendation.

Supplementary Table 21. The test results of photocatalyst recommendation

**Experiment***	Top-1	Top-3	Top-5	Top-10
1	90.55%	95.09%	96.07%	99.19%
2	91.56%	95.17%	95.75%	97.48%
3	89.85%	94.52%	95.77%	98.08%
4	90.90%	95.00%	94.34%	97.50%
5	87.24%	95.91%	96.17%	97.43%
average	90.02%	95.13%	95.62%	97.93%

2.4.2 The test results of base or acid recommendation.

Supplementary Table 22. The test results of base or acid recommendation

**Experiment***	Top-1	Top-3	Top-5	Top-10
1	44.58%	48.68%	49.03%	49.60%
2	47.03%	50.96%	51.39%	51.85%
3	45.83%	50.84%	51.66%	52.30%
4	45.61%	50.01%	50.96%	51.51%
5	47.10%	49.50%	51.69%	52.48%
average	46.03%	49.99%	50.95%	51.54%

2.4.3 The test results of wavelength recommendation.

Supplementary Table 23. The test results of wavelength recommendation

**Experiment***	Top-1	Top-3	Top-5	Top-10
1	97.55%	97.72%	97.99%	98.22%
2	96.12%	97.25%	97.92%	98.96%
3	92.18%	96.51%	97.75%	98.93%
4	99.05%	98.43%	98.04%	95.91%
5	97.65%	98.29%	97.94%	98.61%
average	96.51%	97.64%	97.93%	98.13%

2.4.4 The test results of additive recommendation.

Supplementary Table 24. The test results of additive recommendation

**Experiment***	Top-1	Top-3	Top-5	Top-10
1	49.87%	53.32%	53.35%	54.70%
2	49.82%	50.21%	51.50%	54.28%
3	50.61%	53.67%	55.72%	56.85%
4	49.85%	52.98%	53.01%	53.88%
5	48.72%	52.72%	53.64%	54.97%
average	49.77%	52.58%	53.44%	54.93%

2.4.5 The test results of solvent recommendation.

Supplementary Table 25. The test results of solvent recommendation

**Experiment***	Top-1	Top-3	Top-5	Top-10
1	87.76%	91.82%	93.45%	98.86%
2	88.43%	93.63%	95.76%	97.44%
3	88.79%	93.84%	95.11%	95.90%
4	88.41%	94.38%	94.96%	97.86%
5	89.00%	93.42%	95.95%	97.72%
average	88.48%	93.42%	95.05%	97.56%

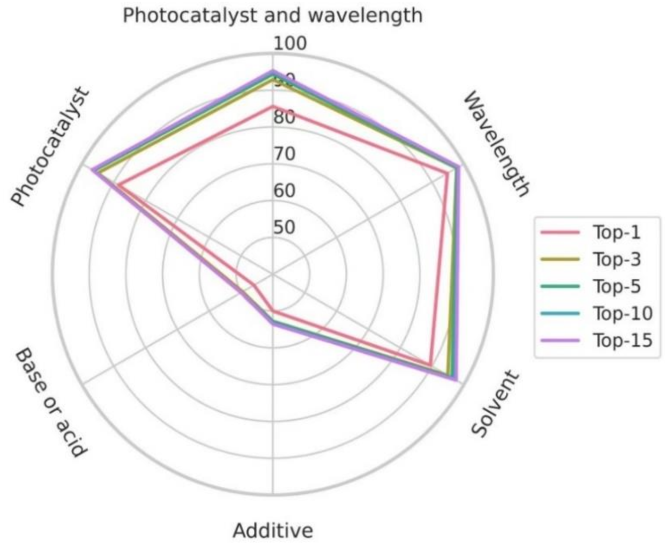

Supplementary Figure 9. PhotoCat demonstrates optimal accuracy when predicting both photocatalysts and light types simultaneously.

2.4.6 Comparison of reaction condition recommendation models

Supplementary Table 26. Summary of reported performance of condition recommendation models in different reaction domains

Model name	Original dataset(s)	Reaction class / domain	Condition categories predicted	Evaluation metric(s)	Reported Top-1 accuracy in original paper
**PhotoCat-Cond**	PhotoCatDB-Cond	Photocatalytic organic synthesis	Photocatalyst, wavelength, solvent, additive, base/acid	Top-1 per category; overall match	88.50% (photosensitizer), 94.82% (wavelength)
**Parrot-LM-E** (Ref. 64)	USPTO-Cond	General organic reactions	Catalyst, solvent	Top-1 per category	92.5% (catalyst), 50.2% (solvent)
**AR-GCN** (Ref.65)	Suzuki coupling dataset	Cross-coupling reactions (Suzuki)	Catalyst, ligand, solvent	Top-1 per category	64.9% (catalyst), 90.8% (ligand), 72.2% (solvent)
**CIMG-Condition** (Ref.66)	General reaction dataset	General organic reactions	Catalyst, solvent	Top-1 per category	59.0% (catalyst), 93.0% (solvent)

***Note:** The models listed above were trained and evaluated on different datasets and reaction domains. Therefore, the results are not directly comparable. The data are presented for contextual reference only..

2.5 Graph2SMILES with Condition Encoding for Reaction Prediction

In this study, we retrained the classical graph-based model Graph2SMILES proposed by Tu et al.[1] for the task of forward photocatalytic reaction prediction. The model was trained on the PhotocatDB-Conditions dataset. Specifically, SMILES strings of reactants were first converted into molecular graphs to extract structural information, which was then processed through a Graph Attention Network (GAT) encoder. The extracted features were subsequently passed to a decoder, which autoregressively generated the product SMILES string.

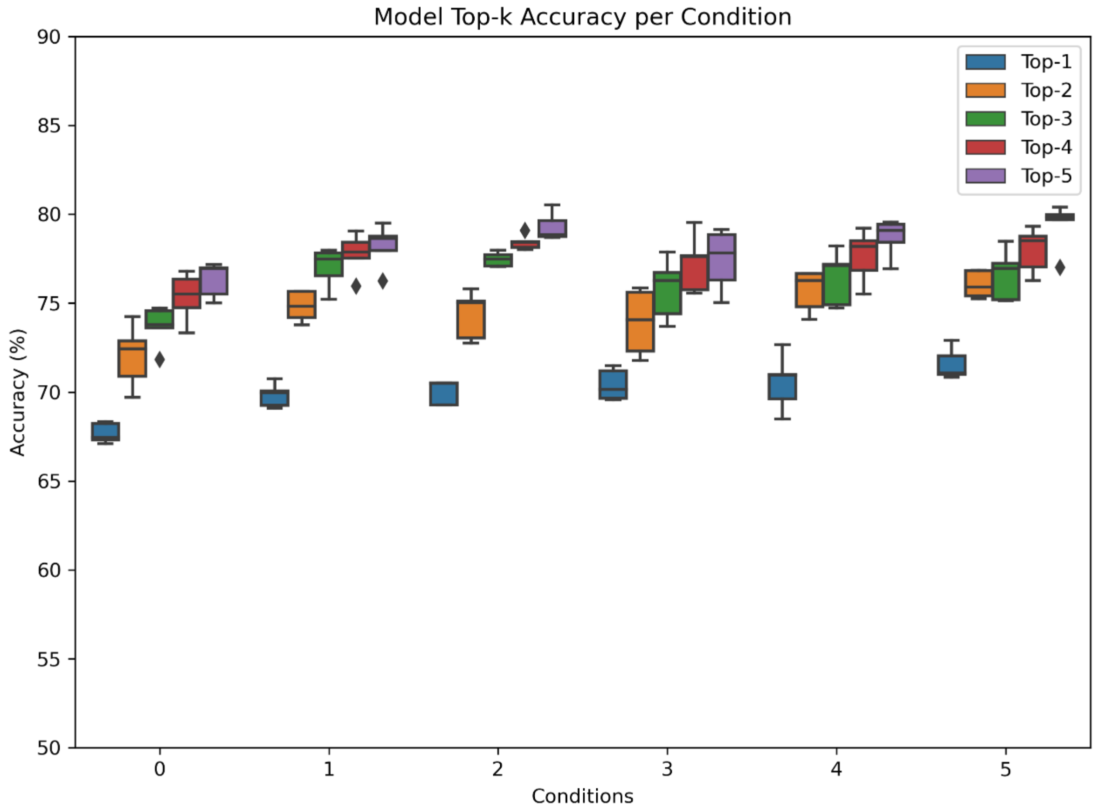

Supplementary Figure 10. Performance of the Graph2SMILES model with reaction condition inputs. Box plots show Top-1 to Top-5 prediction accuracy across different test splits. The consistent improvement from Top-1 to Top-5 demonstrates that incorporating reaction condition information benefits not only transformer-based architectures but also graph neural network-based models, confirming the generality of condition-aware inputs.

The original model, however, was not designed for multi-class conditioning, as it did not explicitly embed reaction condition properties into the graph encoder (Fig. 2). To address this, we modified the model to incorporate reaction condition class labels.

These labels were concatenated with the node features of the reactant graphs and subsequently used as encoding memory to guide the decoder during generation. Concretely, we replaced the original single integer representation of conditions with five placeholder vectors to represent the five key types of reaction conditions in our photocatalysis setting.

As shown in Supplementary Figure 10, the modified Graph2SMILES model also benefits from incorporating reaction conditions: box plots of Top-1 to Top-5 prediction accuracy demonstrate consistent improvements across different test splits. These results indicate that the advantage of condition-aware inputs generalizes beyond transformer-based architectures and extends to graph neural network models, thereby confirming the robustness of this strategy.

2.6 Reaction Conditions as Key Determinants: Case Studies

Reaction conditions provide essential contextual information that goes beyond molecular structures and are therefore critical for accurate prediction of photocatalytic transformations. In many cases, identical reactants can undergo different pathways depending on the photocatalyst, wavelength, or solvent, and such distinctions cannot be resolved from reactant structures alone. By encoding these variables, models are better able to disambiguate structurally similar reactions and produce chemically realistic outcomes.

For example, when an asymmetric catalyst is involved, the product distribution is strongly influenced by the conformation of the ligand[2] (Supplementary Figure 11). This scenario also demonstrates the synergistic or uncoordinated interplay between the photocatalyst and additives, as the combination of different photosensitizers and additives can significantly alter product configuration. Such patterns suggest that the models may have acquired reasoning strategies analogous to those used by human chemists.

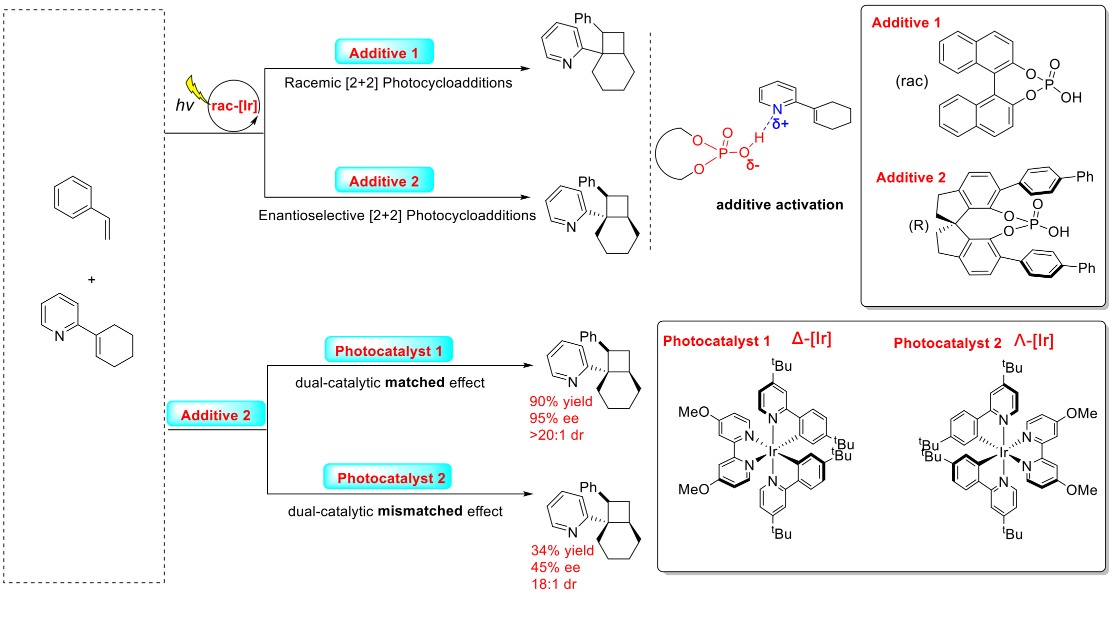

Supplementary Figure 11. Example where reaction conditions interact and determine the main products of photocatalytic reactions. The combination of different photosensitizers and additives can also influence the product's configuration.

Similarly, reaction conditions such as solvent choice can decisively shape product

selectivity. The same transformation yields entirely different products in DMSO versus MeCN[3] (Supplementary Figure12). In these cases, the model implicitly accounts for co-factors such as additives that facilitate photocatalysts, electron-transfer agents, or co-oxidation systems, which together influence the reaction mechanism.

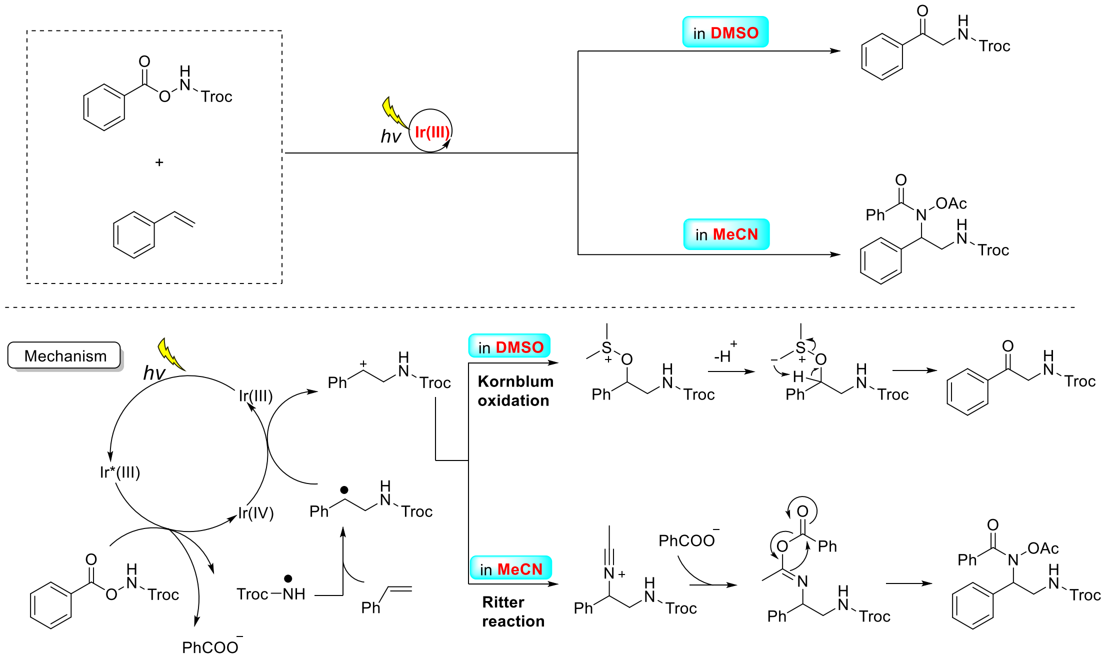

Supplementary Figure12. Case where reaction conditions determine the primary products of photocatalytic reactions. In varying solvent, reactions follow distinct mechanisms, yielding different primary products.

Taken together, these examples underscore that the improved performance observed with condition-aware models arises because reaction condition variables jointly determine reactivity and selectivity. By capturing these multivariate effects, the models not only achieve higher accuracy but also more faithfully reflect chemical reality.

3. Discovery of new photocatalytic reactions.

3.1 Validation of the *PhotoCat-Retro* module

To further justify the utility of the PhotoCat-Retro retrosynthesis module, an illustrative example is provided in Fig. 8, demonstrating how this model generates a non-obvious disconnection and contributes directly to the discovery of one of the experimentally validated photocatalytic reactions (Reaction **b** in the main text, Fig. 10b).

As shown in Fig. 8, through literature survey, we found that previously reported aromatic ketone syntheses involving similar bond disconnections typically proceed via two-electron acylation pathways, which require halogenated arenes and aryl boron derivatives as precursors. These reactions generally involve palladium-complex intermediates, and the preparation of Pd catalysts often demands stringent and complex synthetic procedures[4–6] (*Reaxys reaction IDs: 52389106, 34479583, 50969579*).

In contrast, *PhotoCat-Retro* automatically identified an alternative disconnection for the same target molecule **9**, proposing the coupling of nitrobenzene **7** and pyruvic acid **8** via a radical acylation route. The predicted reaction was subsequently processed by PhotoCat-Cond, which recommended the optimal photocatalytic conditions: *4CzIPN* as photocatalyst, *THF* as solvent, neutral medium, N_2 as atmosphere, and blue-light irradiation (450 nm). Under these conditions, the forward prediction generated by *PhotoCat-RXN* was experimentally confirmed, affording **9** in 70.5 % yield.

This example clearly demonstrates: (i) PhotoCat-Retro proposed a novel disconnection distinct from known metal-catalyzed or acid-mediated routes; (ii) The closed-loop process of *PhotoCat-Retro → PhotoCat-Cond → PhotoCat-RXN* provided a complete, machine-generated synthesis plan that successfully guided laboratory validation. (iii) The reaction proceeded through a radical pathway, as supported by luminescence-quenching and EPR studies described in the main text (Fig. 10f–i).

Collectively, these results confirm that PhotoCat-Retro is a functional and generative retrosynthetic engine, capable of identifying chemically meaningful and experimentally feasible photocatalytic disconnections that extend beyond simple database retrieval or rule-based template matching.

3.2 Statistical transparency and summary of experimental validation

To clarify the overall performance and transparency of the PhotoCat framework, a statistical summary of PhotoCat predictions and experimental validation was compiled (Supplementary Table 27). *PhotoCat* generated a total of 22 photocatalytic reaction predictions, among which 17 reactions showed similarity to previously reported literature examples and were therefore considered less innovative (Table S28). The remaining 5 reactions, identified through literature analysis, were regarded as methodologically novel. These 5 reactions were further evaluated through expert review and analyzed using the *PhotoCat-Cond* and *PhotoCat-RXN* modules, confirming their chemical feasibility. Subsequently, all 5 reactions were experimentally tested, and 4 of them were successfully realized under the predicted photocatalytic conditions (Table S29, corresponding to Reactions **b** – **e** in the main text). This analysis provides a transparent overview of the prediction-validation process, illustrating the practical performance of the PhotoCat framework.

Supplementary Table 27. Summary of model predictions and experimental validation in the PhotoCat workflow.

Stage	Number	Description
Total predictions	22	Reactions proposed by PhotoCat-Retro
Excluded	17	Similar to reported literature examples; not novel
Deemed feasible	5	Evaluated by expert review + PhotoCat-RXN + PhotoCat-Cond
Experimentally tested	5	Carried out under predicted conditions
Successfully realized	4	Correspond to validated reactions (Reaction **b**–**e**)

Supplementary Table 27 lists the specific reactions that were predicted as feasible and selected for experimental testing. Four reactions were successfully reproduced as predicted, while one reaction (Entry 5) failed to yield the target product **22**, instead affording an unexpected side product **23** in 67% isolate yield.

Supplementary Table 28. PhotoCat-generated reactions similar to reported literature examples (17 reactions, not experimentally validated)

Entry	Reactions proposed by PhotoCat	Similar to previous reports
1	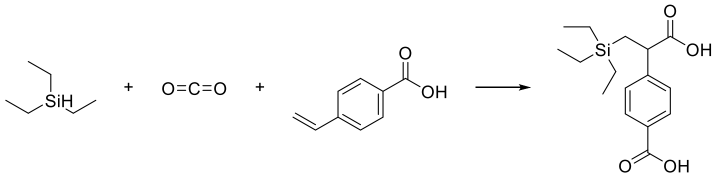 CC[SiH](CC)CC.O=C=O.C=CC1=CC=C(C(O)=O)C=C1>>O=C(C2=CC=C(C(C(O)=O)C[Si](CC)(CC)CC)C=C2)O	Ref [7]
2	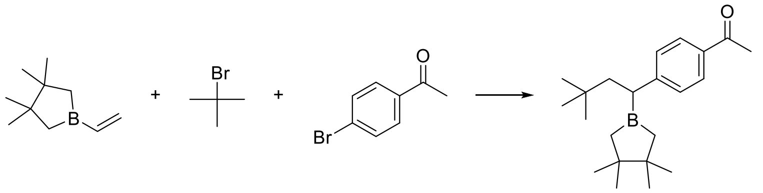 C=CB1CC(C)(C)C(C)(C)C1.CC(C)(Br)C.BrC2=CC=C(C(C)=O)C=C2>>CC(C(C)(C)C3)(C)CB3C(CC(C)(C)C)C4=CC=C(C(C)=O)C=C4	Ref [8]
3	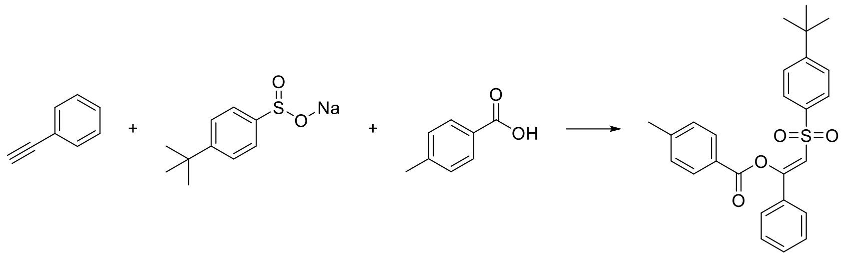 C#CC1=CC=CC=C1.O=S(C2=CC=C(C(C)(C)C)C=C2)O[Na].O=C(C3=CC=C(C)C=C3)O>>O=C(C4=CC=C(C)C=C4)O/C(C5=CC=CC=C5)=C\S(C6=CC=C(C(C)(C)C)C=C6)(=O)=O	Ref [9]
4	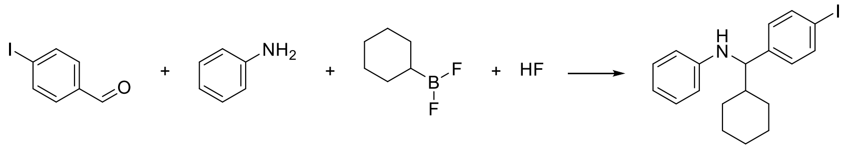 IC1=CC=C(C=O)C=C1.NC2=CC=CC=C2.FB(C3CCCCC3)F.[F-].[K+]>>IC4=CC=C(C(C5CCCCC5)NC6=CC=CC=C6)C=C4	Ref [10]
5	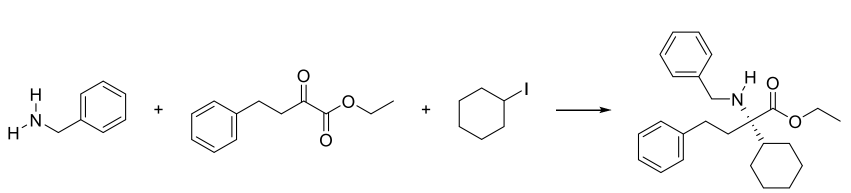 [H]N(CC1=CC=CC=C1)[H].O=C(CCC2=CC=CC=C2)C(OCC)=O.IC3CCCCC3>>[H]N(CC4=CC=CC=C4)[C@@](C5CCCCC5)(C(OCC)=O)CCC6=CC=CC=C6	Ref [11]
6	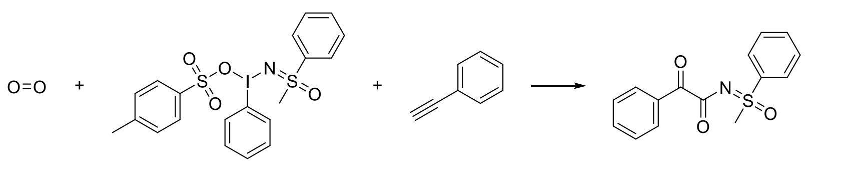 O=O.CS(=NI(C1=CC=CC=C1)OS(C2=CC=C(C)C=C2)(=O)=O)(C3=CC=CC=C3)=O.C#CC4=CC=CC=C4>>O=C(C(N=S(C)(C5=CC=CC=C5)=O)=O)C6=CC=CC=C6	Ref [12]

Entry	Reactions proposed by PhotoCat	Similar to previous reports
7	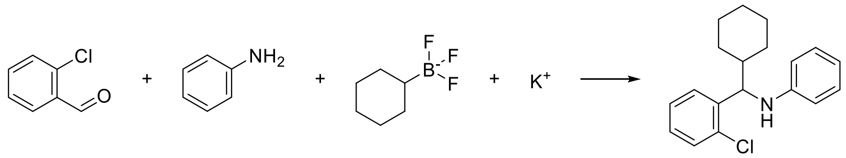<br>O=CC1=C(Cl)C=CC=C1.NC2=CC=CC=C2.F[B-](C3CCCCC3)(F)F.[K+]>>ClC(C=CC=C4)=C4C(C5CCCCC5)NC6=CC=CC=C6	Ref [10]
8	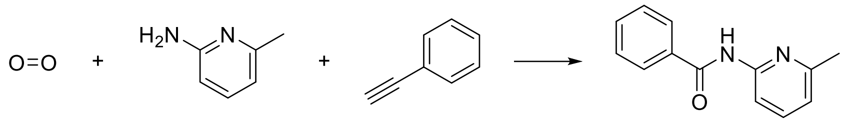<br>O=O.NC1=NC(C)=CC=C1.C#CC2=CC=CC=C2>>O=C(C3=CC=CC=C3)NC4=NC(C)=CC=C4	Ref [13]
9	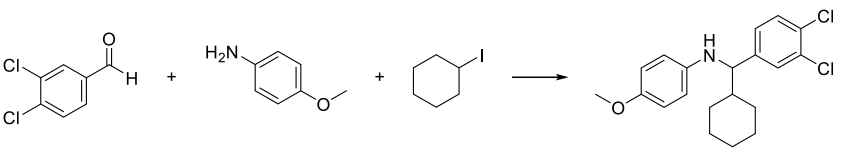<br>ClC1=CC=C(C([H])=O)C=C1Cl.NC2=CC=C(OC)C=C2.IC3CCCCC3>>ClC4=CC=C(C(NC5=CC=C(OC)C=C5)C6CCCCC6)C=C4Cl	Ref [14]
10	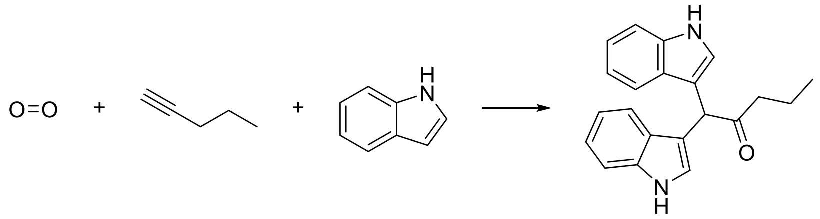<br>O=O.C#CCCC.C12=CC=CC=C1C=CN2>>O=C(CCC)C(C3=CNC4=C3C=CC=C4)C5=CNC6=CC=CC=C65	Ref [15]
11	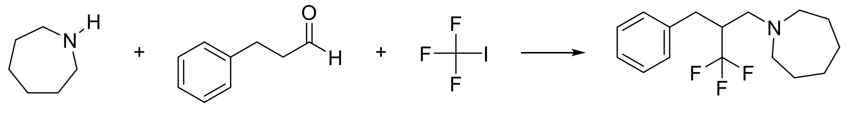<br>[H]N1CCCCCC1.[H]C(CCC2=CC=CC=C2)=O.FC(F)(I)F>>FC(C(CC3=CC=CC=C3)CN4CCCCCC4)(F)F	Ref [16]
12	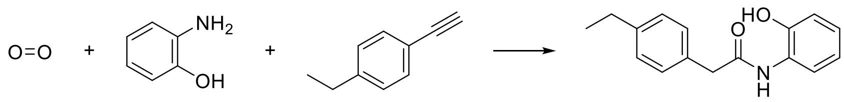<br>O=O.NC1=C(O)C=CC=C1.C#CC2=CC=C(CC)C=C2>>O=C(CC3=CC=C(CC)C=C3)NC4=C(O)C=CC=C4	Ref [17]

Entry	Reactions proposed by PhotoCat	Similar to previous reports
13	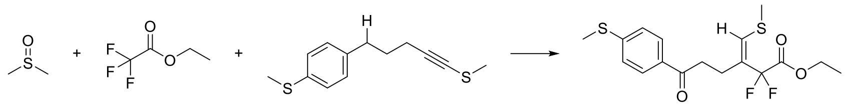 CS(C)=O.FC(F)(C(OCC)=O)F.[H]C(C1=CC=C(SC)C=C1)CCC#CSC>>O=C(C2=CC=C(SC)C=C2)CC/C(C(F)(C(OCC)=O)F)=C([H])/SC	Ref [18]
14	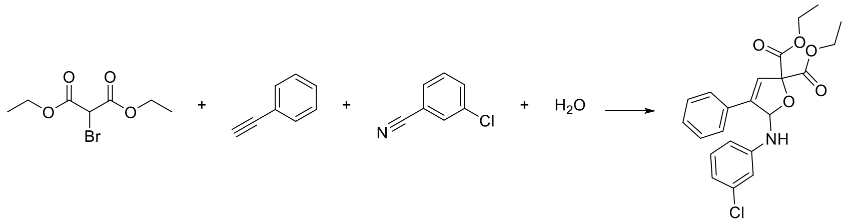 BrC(C(OCC)=O)C(OCC)=O.C#CC1=CC=CC=C1.ClC2=CC=CC(C#N)=C2.O>>ClC3=CC(NC4OC(C=C4C5=CC=CC=C5)(C(OCC)=O)C(OCC)=O)=CC=C3	Ref [19]
15	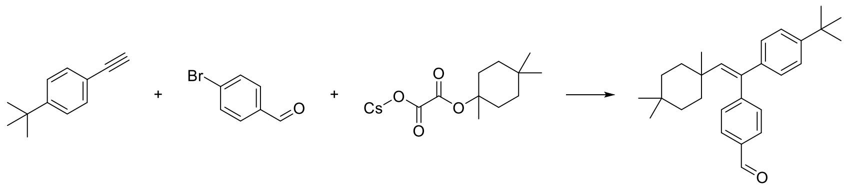 C#CC1=CC=C(C(C)(C)C)C=C1.BrC2=CC=C(C=O)C=C2.O=C(C(O[Cs])=O)OC3(C)CCC(C)(C)CC3>>O=CC4=CC=C(/C(C5=CC=C(C(C)(C)C)C=C5)=C\C6(C)CCC(C)(C)CC6)C=C4	Ref [20]
16	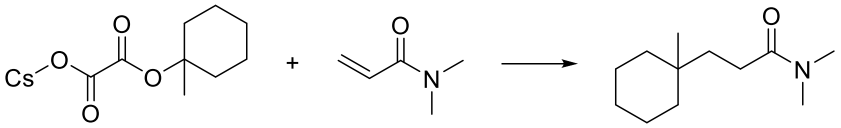 CC1(OC(C(O[Cs])=O)=O)CCCCC1.C=CC(N(C)C)=O>>O=C(N(C)C)CCC2(C)CCCCC2	Ref [21]
17	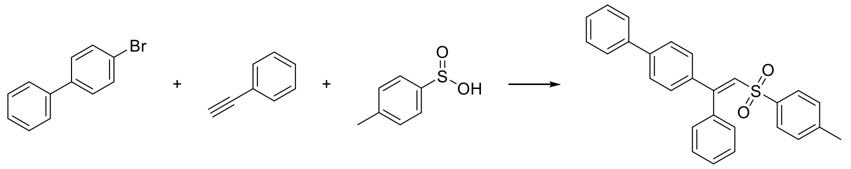 BrC1=CC=C(C2=CC=CC=C2)C=C1.C#CC3=CC=CC=C3.CC4=CC=C(S(O)=O)C=C4>>CC5=CC=C(S(/C=C(C6=CC=CC=C6)/C7=CC=C(C8=CC=CC=C8)C=C7)(=O)=O)C=C5	Ref [22]

Supplementary Table 29. List of reactions predicted as feasible and experimentally tested.

Entry	Deemed feasible (by expert & PhotoCat), and Attempted experimentally	Successfully realized	Note
**1**	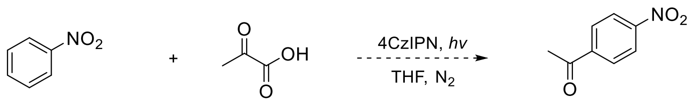 O=[N+](C1=CC=CC=C1)[O-].CC(C(O)=O)=O>>CC(C2=CC=C([N+]([O-])=O)C=C2)=O	√	Reaction **b**
**2**	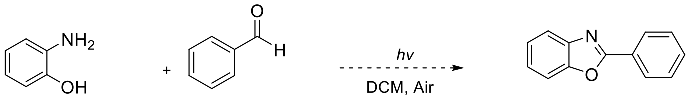 OC1=CC=CC=C1N.[H]C(C2=CC=CC=C2)=O>>C34=CC=CC=C3N=C(C5=CC=CC=C5)O4	√	Reaction **c**
**3**	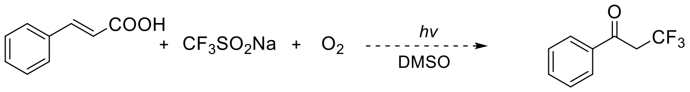 O=C(/C=C/C1=CC=CC=C1)O.FC(F)(S(O[Na])=O)F.O=O>>O=C(CC(F)(F)F)C2=CC=CC=C2	√	Reaction **d**
**4**	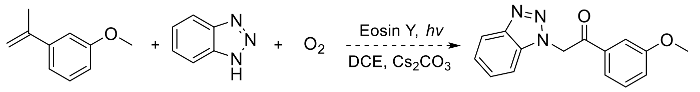 C=C(C)C1=CC=CC(OC)=C1.O=O.C23=CC=CC=C2N=NN3>>COC4=CC(C(CN5N=NC6=CC=CC=C65)=O)=CC=C4	√	Reaction **e**
**5**	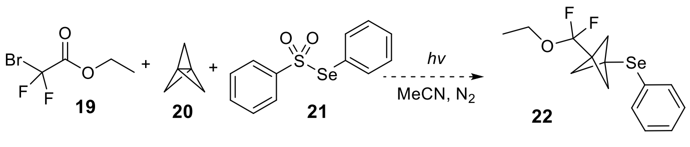 O=C(OCC)C(F)(F)Br.O=S(C1=CC=CC=C1)([Se]C2=CC=CC=C2)=O.C3C45C3(C5)C4>>FC(OCC)(F)C67CC(C7)([Se]C8=CC=CC=C8)C6	×	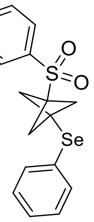 23 (Main product **23**, 67% isolate yield)

3.3 The novelty of the four photocatalytic reactions

Supplementary Table 30. Literature comparators and novelty of the photocatalytic reactions in **Figure 10**.

Reaction	Transformation	Closest Literature Comparator	Key Differences / Novelty
**b**	Aromatic ketone formation from pyruvate + nitrobenzene	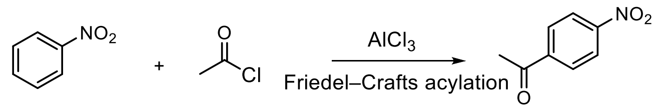<br>♦ Strong EWG➞para Friedel–Crafts acylation difficult<br>♦ Requires excess $AlCl_3$ + acyl chloride<br>(Ref. 67, *Chem. Rev.* **2006**, *106*, 1077)	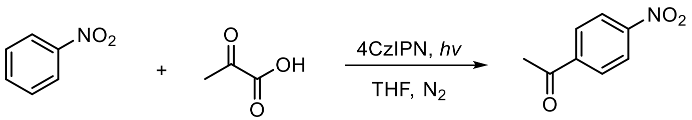<br>♦ Mild photocatalysis<br>♦ Glyoxylic acid as nitrating reagent<br>♦ High selectivity, para-acylation yield 70.5%<br>♦ No excess Lewis acid
**c**	2-Phenylbenzoxazole formation from 2-aminophenol + benzaldehyde	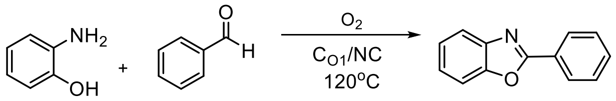<br>♦ High-cost catalyst prep/characterization<br>♦ Atomically dispersed Co/N－C via ZnCo-ZIF pyrolysis<br>♦ Reaction at 120°C； O_2 required<br>(Ref. 68, *Green Chem.* **2024**, *26*, 4834)<br>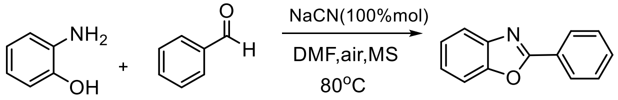<br>♦ Requires 100 mol% NaCN<br>♦ Reaction at 80°C<br>(Ref. 69, *Adv. Synth. Catal.* **2012**, *354*, 2992)	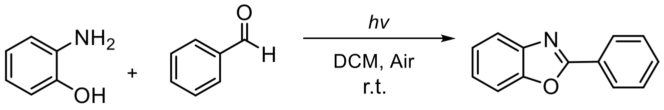<br>♦ No additional catalyst required<br>♦ Mild light irradiation<br>♦ Room-temperature reaction<br>♦ Air as the oxygen source

Reaction	Transformation	Closest Literature Comparator	Key Differences / Novelty
**d**	α-Trifluoromethyl ketone formation from cinnamic acid + CF_3SO_2Na	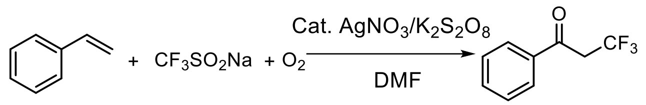<br>• Requires $AgNO_3$ catalyst<br>• Additional oxidant needed<br>(Ref. 69, *Angew. Chem.* **2013**, *52*, 9747)	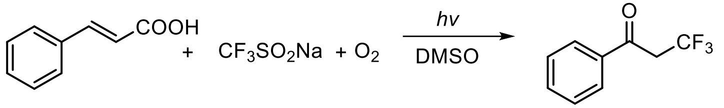<br>• Metal-free,<br>• Oxidant-free (open flask)<br>• No additional catalyst required<br>• Mild light irradiation
**e**	Oxo-amination of alkenes to α-amino ketones	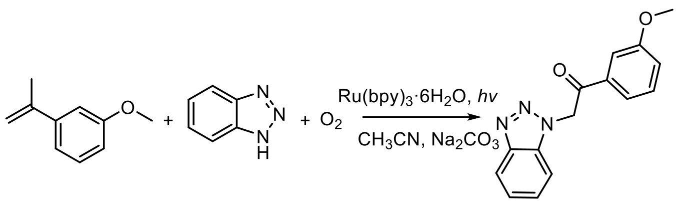<br>(Ref. 73, *Org. Lett.* **2023***, 25, 5333*)	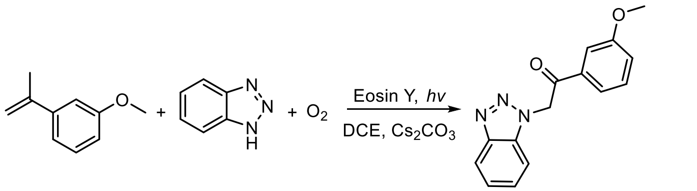<br>• Minor modifications to existing work<br>• Single-step vicinal oxo-amination of unactivated alkenes.

3.4 Chemistry synthesis

3.4.1 Reaction b

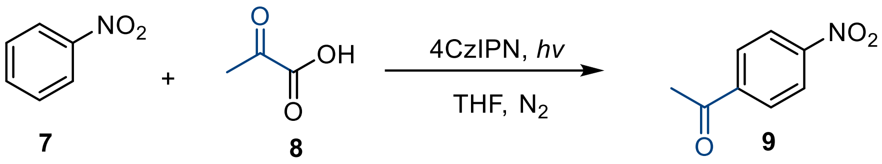

General procedure: A mixture of nitrobenzene **7** (0.2 mmol), pyruvic acid **8** (0.4 mmol), and THF (2 mL) were added to a reaction tube. The tube was evacuated and backfilled with N_2 for three times. The mixture was then irradiated by 360–365 nm (10 w) for 24 h. After completion of the reaction, the resulting mixture was extracted with CH_2Cl_2, and the organic phase was then removed under vacuum. The residue was purified by column chromatography using a mixture of petroleum ether and ethyl acetate as eluent to give the desired product **9** with 70% yield.

3.4.2 Reaction c

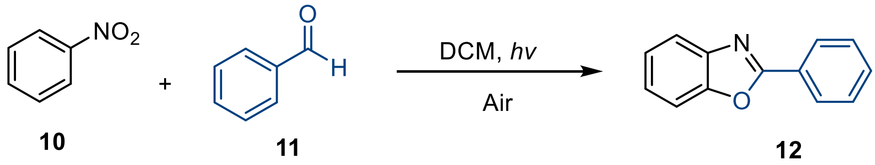

General procedure: A mixture of 2-aminophenol **10** (0.2 mmol), benzaldehyde **11** (0.4 mmol), and DCM (2 mL) were added to a reaction tube. The reaction mixture was open to the air and stirred at room temperature under the irradiation of a 390 nm LED lamp for 48 h. After completion of the reaction, the resulting mixture was extracted with CH_2Cl_2, and the organic phase was then removed under vacuum. The residue was purified by column chromatography using a mixture of petroleum ether and ethyl acetate as eluent to give the desired product **12** with 73% yield.

3.4.3 Reaction d

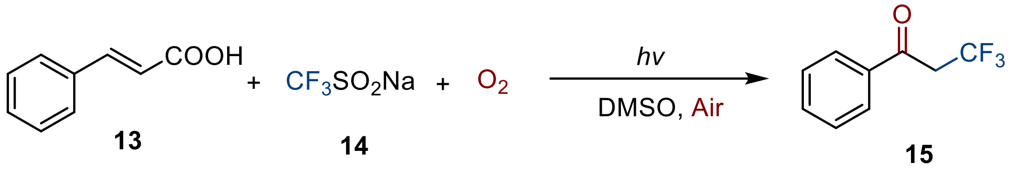

General procedure: A mixture of cinnamic acid **13** (0.2 mmol), CF_3SO_2Na **14** (0.4 mmol), and DMSO (2 mL) were added to a reaction tube. The reaction mixture was opened to the air and stirred at room temperature under the irradiation of purple light for 5 h. After completion of the reaction, the resulting mixture was extracted with CH_2Cl_2, and the organic phase was then removed under vacuum. The residue was purified by column chromatography using a mixture of petroleum ether and ethyl acetate as eluent to give the desired product **15** with 75% yield.

3.4.4 Reaction e

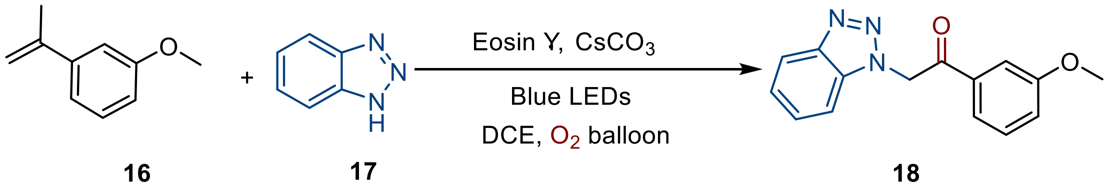

General procedure: In an oven-dried reaction tube equipped with a magnetic stirrer bar was charged with α-methylstyrene **16** (0.9 mmol), benzotriazole **17** (0.3 mmol), caesium carbonate (0.9 mmol), Eosin Y (3.0 mol %) and DCE (2.0 mL). The tube was then exposed to blue LEDs irradiation at room temperature under O_2 atmosphere with stirring for 36 h. After completion of the reaction, the resulting mixture was extracted with CH_2Cl_2, and the organic phase was then removed under vacuum. The residue was purified by column chromatography using a mixture of petroleum ether and ethyl acetate as eluent to give the desired product **18** with a 63% yield.

3.4.5 reaction 5 (Table S29)

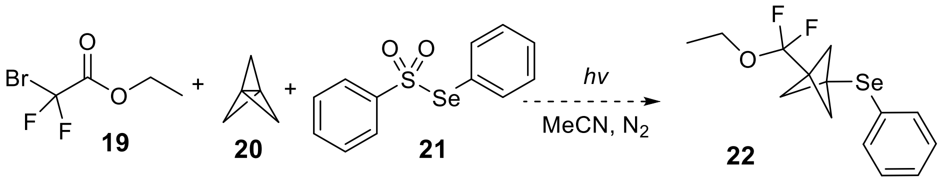

General procedure: A mixture of ethyl bromodifluoroacetate **19** (0.2 mmol), bicyclo[1.1.1]pentane **20** (0.4 mmol), and selenosulfonate **21** (0.2 mmol) in acetonitrile (2 mL) was placed in a reaction tube. The tube was evacuated and purged with nitrogen three times. The resulting mixture was then irradiated with 400–405 nm light for 24 h. Upon completion, the reaction mixture was extracted with dichloromethane, and the combined organic layers were concentrated under reduced pressure. The crude residue was purified by column chromatography using a petroleum ether/ethyl acetate mixture as the eluent. Unexpectedly, the desired product **22** was not obtained; instead, product **23** was isolated in 67% yield**.**

3.5 Mechanism investigation

3.5.1. Control experiment

Under the standard conditions of the four photocatalytic reactions, the transformation is significantly inhibited by avoiding light or adding radical trapping agents. These phenomena suggest that light is necessary for the four reaction pathways and these involve a radical pathway.

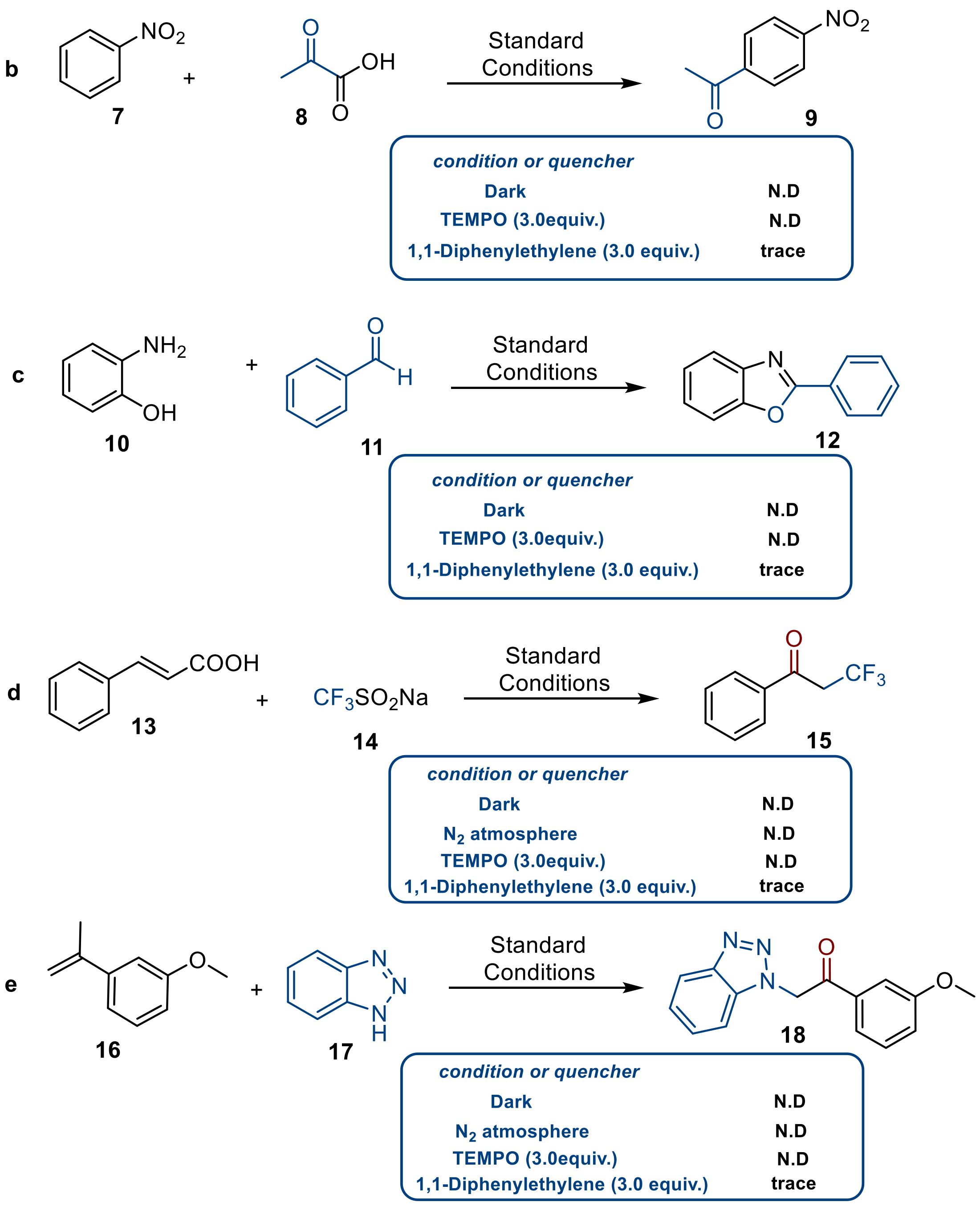

Supplementary Figure 13. Control experiment.

3.5.2 Luminescence Quenching Screening Studies (Reaction b)

Emission intensities were recorded using a F-7000 FL Spectrophotometer, First, the emission intensity of 4CzIPN solutions was observed at 520 nm. The solutions were irradiated at 475 nm and fluorescence was measured from 400 nm to 750 nm. The emission spectrum of a 5x10^{-5} M solution of 4CzIPN with different concentrations of pyruvic acid in degassed anhydrous THF in 1 cm cuvette with a sealed stopper was collected.

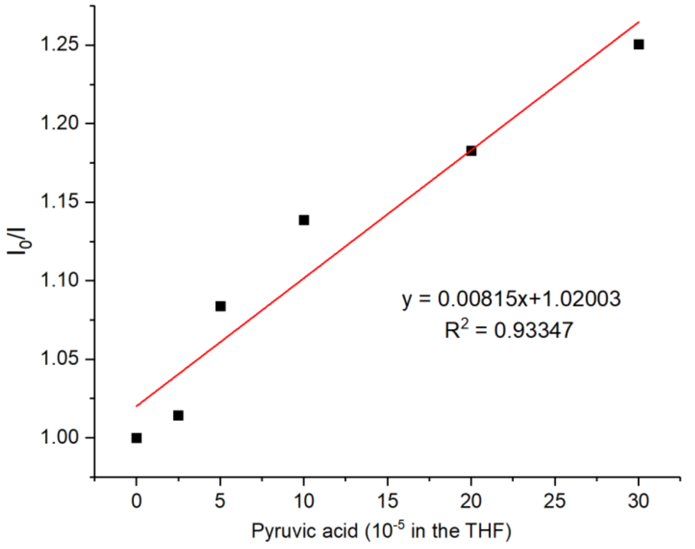

Supplementary Figure 14. Stern-Volmer plot of Fluorescence quenching of 4CzIPN by pyruvic acid

3.5.3 UV-visible absorption Spectra (Reaction d)

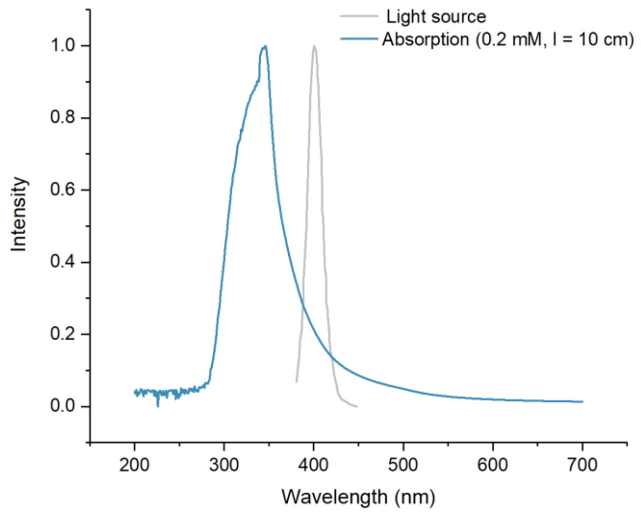

Supplementary Figure 15. Absorption spectra of **8** (dissolved in DMSO)

There is a substantial overlap between the absorption spectrum of cinnamic acid **8** and the emission wavelength, which indicates that **8** can absorb the corresponding wavelengths to form the excited species.

3.5.4 EPR experiment (Reaction d)

For further explore the active species of singlet oxygen involved in the reaction, 2,2,6,6-tetramethylpiperidine (TEMP) were used to trap $^1O_2$ (g = 2.0065). Irradiation of the reaction solution of TEMP with cinnamic acid **13** and CF_3SO_2Na **14** in DMSO under air with purple LEDs resulted in the formation of a strong characteristic signal $^1O_2$ adduct with TEMP (Figure S6), implying that $^1O_2$ is also present during the reaction.

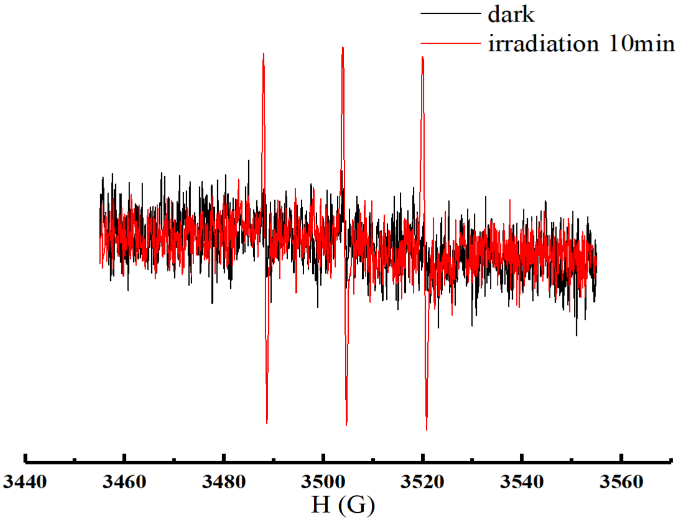

Supplementary Figure 16. Electron spin resonance (ESR) spectra of TEMP with $^1O_2$

(a) A solution of TEMP (0.20 mol/L) with cinnamic acid **13** and CF_3SO_2Na **14** in air-saturated DMSO without light irradiation.

(b) A solution of TEMP (0.2 mol/L) with cinnamic acid **13** and CF_3SO_2Na **14** in air-saturated DMSO under purple LEDs irradiation for 10min.

In order to determine the active species of oxygen involved in the present reaction, 5,5-dimethyl-pyrroline-*N*-oxide (DMPO) were employed to capture $O_2^{\bullet-}$ (g = 2.0069). There was no signal when DMPO was added into air-saturated DMSO solution of cinnamic acid **13** and CF_3SO_2Na **14** without light irradiation (Supplementary Figure 16a). Irradiation of the above solution in air with purple LEDs resulted in the formation of a strong characteristic signal of $O_2^{\bullet-}$ adduct with DMPO (Supplementary Figure 16b), indicating the formation of $O_2^{\bullet-}$ in the reaction.

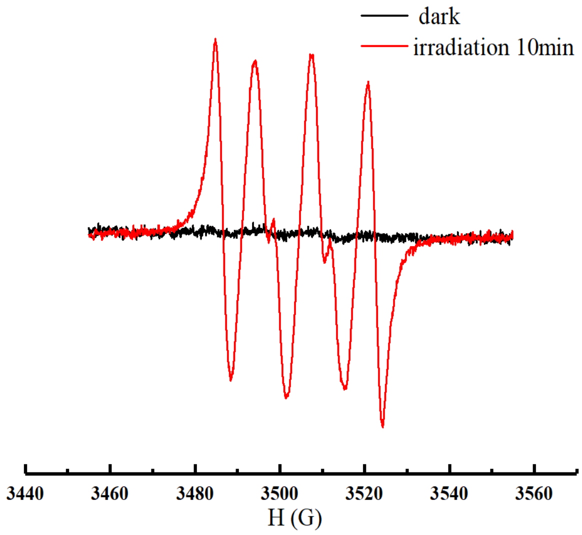

Supplementary Figure 17. Electron spin resonance (ESR) spectra of DMPO with $O_2^{\bullet-}$

(a) A solution of DMPO (0.20 mol/L) with cinnamic acid **13** and CF_3SO_2Na **14** in air-saturated DMSO without light irradiation.

(b) A solution of DMPO (0.2 mol/L) with cinnamic acid **13** and CF_3SO_2Na **14** in air-saturated DMSO under purple LEDs irradiation for 10min.

3.6 Proposed mechanism

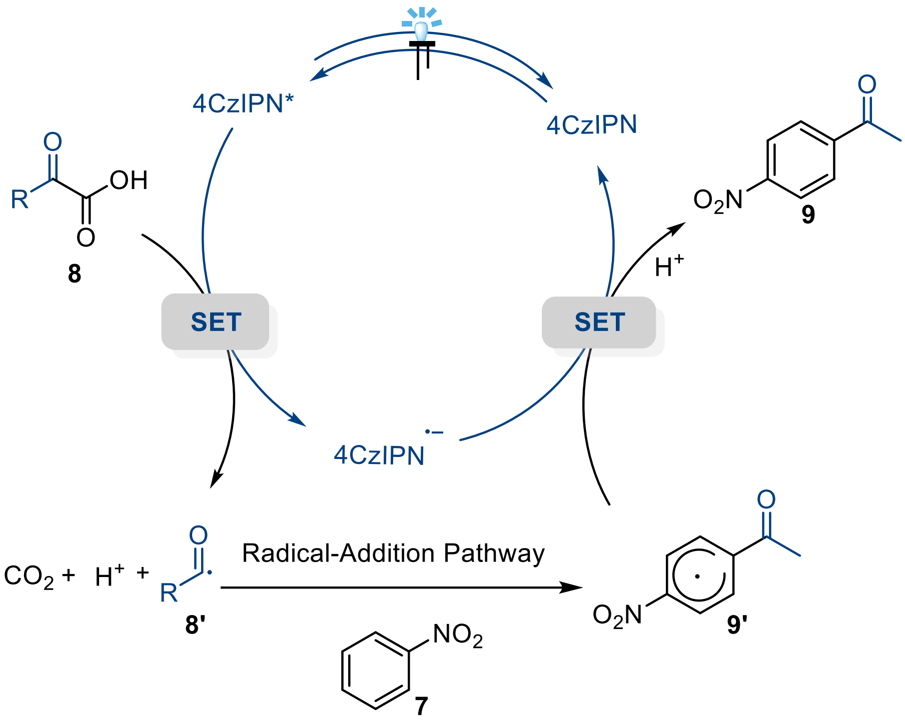

Supplementary Scheme 1. Possible Mechanisms for Reaction **b**

Based on the previous reports[23–25] and the control experiment results, a reasonable mechanism is proposed in Scheme S1. The photocatalyst 4CzIPN leaps from the ground state to the excited state under visible light irradiation and undergoes a single electron transfer (SET) with pyruvic acid **8** to produce an acyl radical **8'**, while 4CzIPN is reduced to the radical anion. The acyl radical **8'** attacked at the nitrobenzene **7** to afford an intermediate **9'**, followed by a single electron transfer with the radical anion state of the photocatalyst to give the final acylation product **9.** Meanwhile, 4CzIPN returns to the ground state, which forms a catalytic cycle.

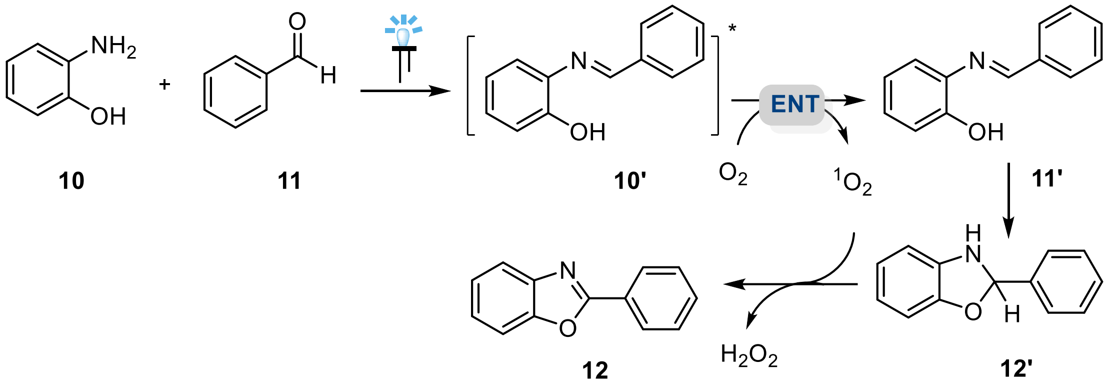

Supplementary Scheme 2. Possible Mechanisms for Reaction **c**.

Based on the previous reports[26,27] and the control experiment results, a reasonable mechanism is proposed in Scheme S2. Initially, 2-aminophenol **10**, benzaldehyde **11** reacted to form intermediate **11'** under blue LEDs irradiation. Then, **10'** is formed as an excited state species upon light irradiation. Subsequently, the ground-state triplet oxygen $^3O_2$ could be converted into the higher active excited-state singlet oxygen $^1O_2$ via an energy transfer (EnT) process between **10'** and $^3O_2$, accompanied with the regeneration of ground state **11'**. The electron-rich OH group underwent intramolecular nucleophilic addition to the C=N bond to form the ring-closure intermediate**12'**. Finally, intermediate **12'** was oxidized by $^1O_2$ to release an H_2O_2 molecule to form the target product **12**.

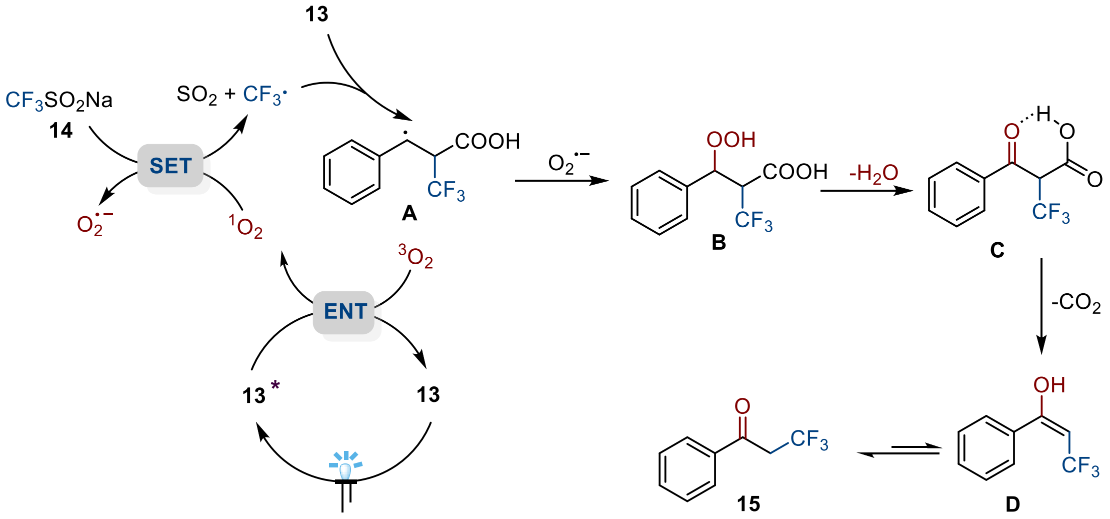

Supplementary Scheme 3. Possible Mechanisms for Reaction **d**

Based on the previous reports[28,29] and the control experiment results, a reasonable mechanism is proposed in Scheme S3. Initially, cinnamic acid **13** was irradiated by

purple LEDs to form the excited species **13***. Subsequently, the ground-state triplet oxygen $^3O_2$ could be converted into the higher active excited-state singlet oxygen $^1O_2$ via an energy transfer (EnT) process between **13*** and $^3O_2$, accompanied with the regeneration of ground state **13**. Then the CF_3SO_2• radical was formed via oxidation of CF_3SO_2Na with active $^1O_2$ through a single electron-transfer (SET) process, which subsequently released a molecule of SO_2 to give the CF_3• radical. The acquired CF_3• attacked at the double bond of cinnamic acid **13** to afford an intermediate **A**. After being oxidized by $O_2^{\bullet -}$, peroxide **B** is formed, which could release a molecule of water to produce ketone compound **C**. Finally, compound **C** via a cyclization-decarboxylation-aromatization process under the standard conditions produces **D**, which isomerizes and forms product **15**.

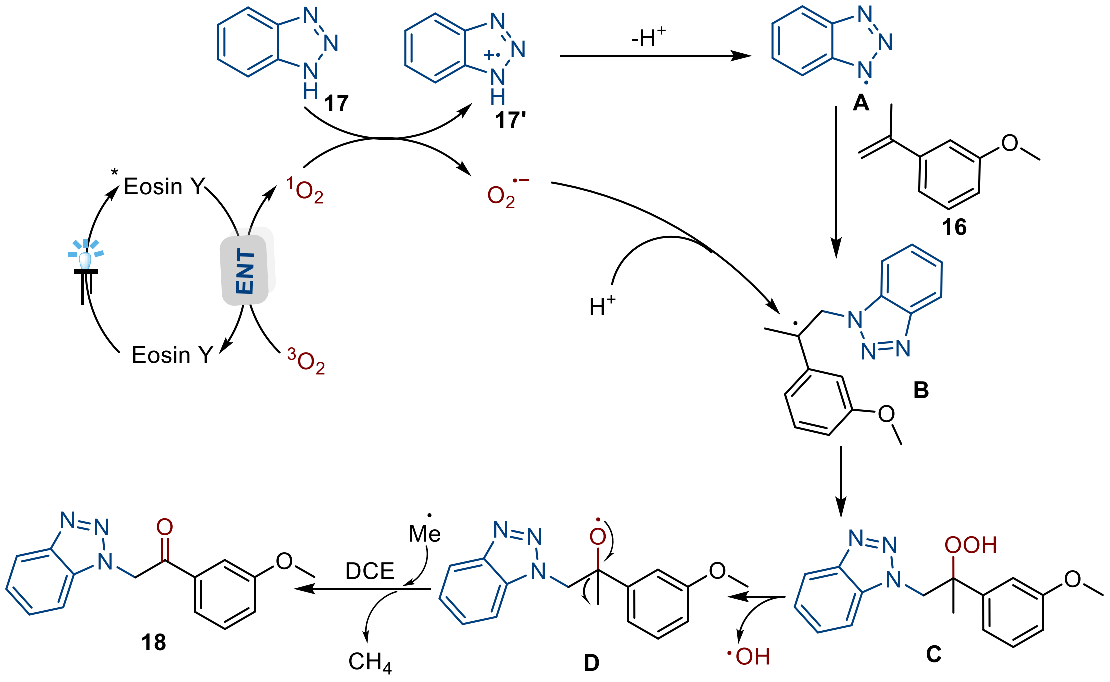

Supplementary Scheme 4. Possible Mechanisms for Reaction **e**

Based on the previous reports[30] and the control experiment results, a reasonable mechanism is proposed in Scheme S4. Initially, Eosin Y absorbs photons under visible light irradiation, leading to the excited species *Eosin Y, which further undergoes an energy transfer (ENT) process with triplet oxygen $^3O_2$ to afford singlet oxygen $^1O_2$ along with the regeneration of Eosin Y. The nitrogen-centered cation radical **17'** is formed by oxidation of singlet $^1O_2$ via a single electron transfer (SET) pathway, followed by deprotonation to give the radical **A**, meanwhile, $^1O_2$ is reduced to the superoxide anion $O_2^{\bullet -}$. Subsequently, the radical addition of **A** to α-methylstyrene **16**

forms the radical intermediate **B**, which further couples with $O_2^{\bullet -}$, followed by protonation to provide peroxide species **C**. The peroxide **C** can immediately proceed the homolysis of the O-O bond to provide the oxygen-centered radical **D**. Finally, the alkoxyl radical **D** is able to weaken the adjacent C-C bonds, causing the formation of desired product **18** via β-scission along with the release of methane resulting from the methyl radical via a H-abstraction process from the reaction mixture.

3.7 Characterization data for the products

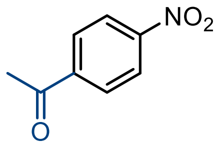

1-(4-nitrophenyl)ethan-1-one (9): Eluent: Hexane/ethyl acetate (10:1). Yield: 70%. white solid, m.p. 78.2–80.4 °C. ^{1}H NMR (400 MHz, $CDCl_3$) δ 8.34 (d, J = 8.7 Hz, 2H), 8.13 (d, J = 8.8 Hz, 2H), 2.70 (s, 3H). ^{13}C NMR (101 MHz, $CDCl_3$) δ 196.30, 150.37, 141.37, 129.32, 123.88, 27.02.

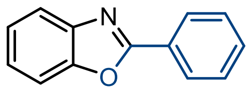

2-phenylbenzoxazole (12)[26]**:** Eluent: Hexane/ethyl acetate (10:1). Yield: 73%. white solid. ^{1}H NMR (400 MHz, $CDCl_3$) δ 8.34-8.26 (m, 2H), 7.84 -7.78 (m, 1H), 7.64 - 7.59 (m, 1H), 7.59 - 7.52 (m, 3H), 7.42 - 7.35 (m, 2H). ^{13}C NMR (101 MHz, $CDCl_3$) δ 163.07, 150.78, 142.08, 131.56, 128.94, 127.66, 127.17, 125.14, 124.61, 120.03, 110.62.

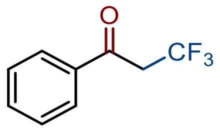

3,3,3-trifluoro-1-phenylpropan-1-one (15): Eluent: Hexane/ethyl acetate (50:1). Yield: 75%. white solid. ^{1}H NMR (400 MHz, $CDCl_3$) δ 7.96 (d, J = 7.2 Hz, 2H), 7.69-7.64 (m,1H), 7.56-7.51 (m, 2H), 3.82 (q, J = 10.0 Hz, 2H). ^{13}C NMR (101 MHz, $CDCl_3$) δ 189.70, 135.82, 134.20, 128.94, 128.35, 124.01 (C-F, $^{1}J_{C\text{-}F}$, J = 278.0 Hz), 42.10 (C-F, $^{2}J_{C\text{-}F}$, J = 28.3 Hz). ^{19}F NMR (376 MHz, $CDCl_3$) δ -62.04; HRMS (ESI) m/z: [M-H]$^{+}$ Calcd for $C_9H_6F_3O$ 187.0365; Found 187.0374.

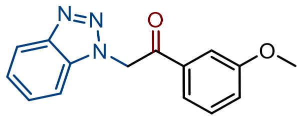

2-(1H-benzo[d][1,2,3]triazol-1-yl)-1-(3-methoxyphenyl)ethan-1-one (18): Eluent: Hexane/ethyl acetate (4:1). Yield: 63%. white solid. ^{1}H NMR (400 MHz, $CDCl_3$) δ 7.95 – 7.90 (m, 2H), 7.61 (d, *J* = 7.6 Hz, 1H), 7.56 – 7.53 (m, 1H), 7.48 – 7.41 (m, 3H), 7.21 (d, *J* = 8.3 Hz, 1H), 6.20 (s, 2H), 3.88 (s, 3H). ^{13}C NMR (101 MHz, $CDCl_3$) δ 190.17, 160.15, 145.04, 135.60, 130.09, 126.73, 120.90, 120.62, 118.25, 112.52, 61.99, 55.53. HRMS (ESI) m/z: $[M+H]^+$ Calcd for $C_{15}H_{14}N_3O_2$ 268.1081; Found 268.1070.

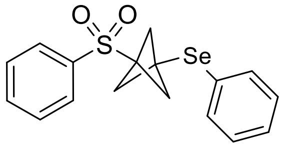

phenyl(3-(phenylsulfonyl)bicyclo[1.1.1]pentan-1-yl) selane (23): Eluent: Hexane/ethyl acetate (10:1). Yield: 67%. white solid, m.p. 79.2–80.5 °C. 1H NMR (400 MHz, CDCl3) δ 7.83 (d, J = 7.2 Hz, 2H), 7.70 – 7.64 (m, 1H), 7.59 – 7.50 (m, 4H), 7.38 – 7.28 (m, 3H), 2.17 (s, 6H). 13C NMR (101 MHz, $CDCl_3$) δ 136.74, 135.92, 133.87, 129.24, 129.23, 128.58, 128.55, 126.95, 54.65, 53.80, 33.86.

3.8 ^{1}H, ^{13}C and ^{19}F NMR spectra of products

4 ^{1}H, ^{13}C and ^{19}F NMR spectra of products

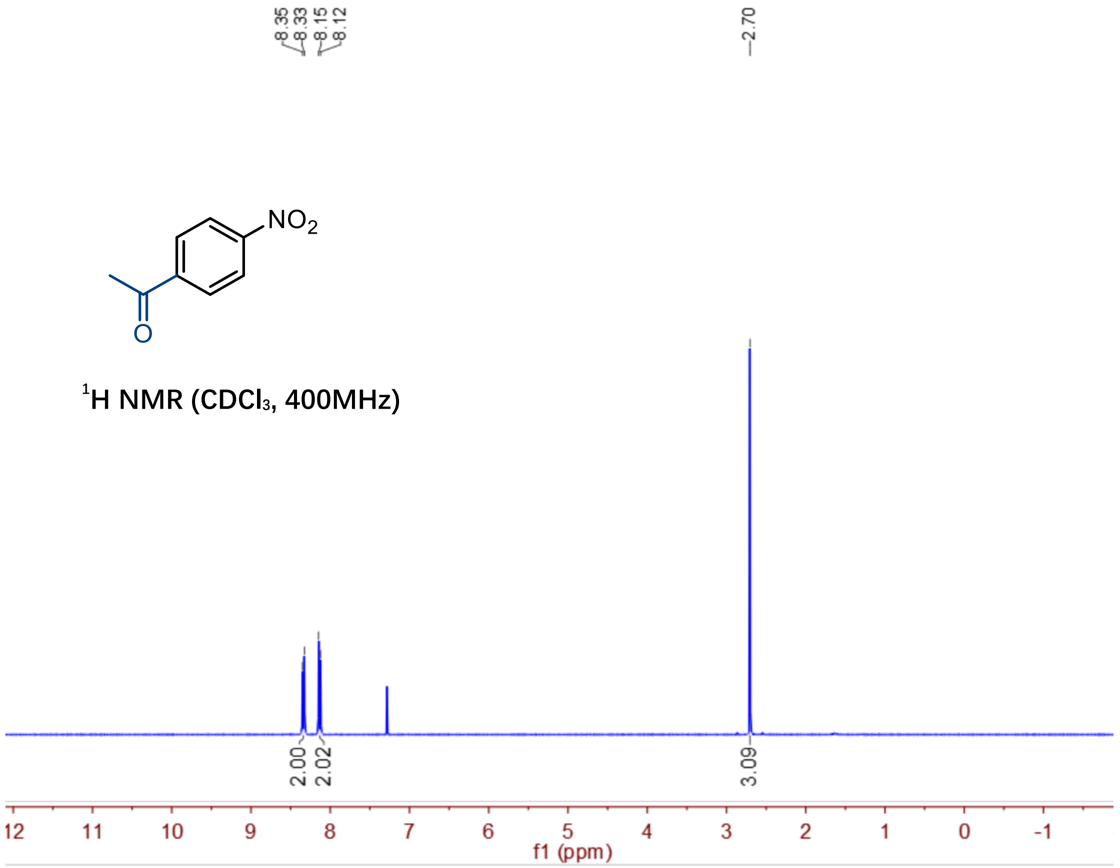

^{1}H NMR Spectrum of Compound 9

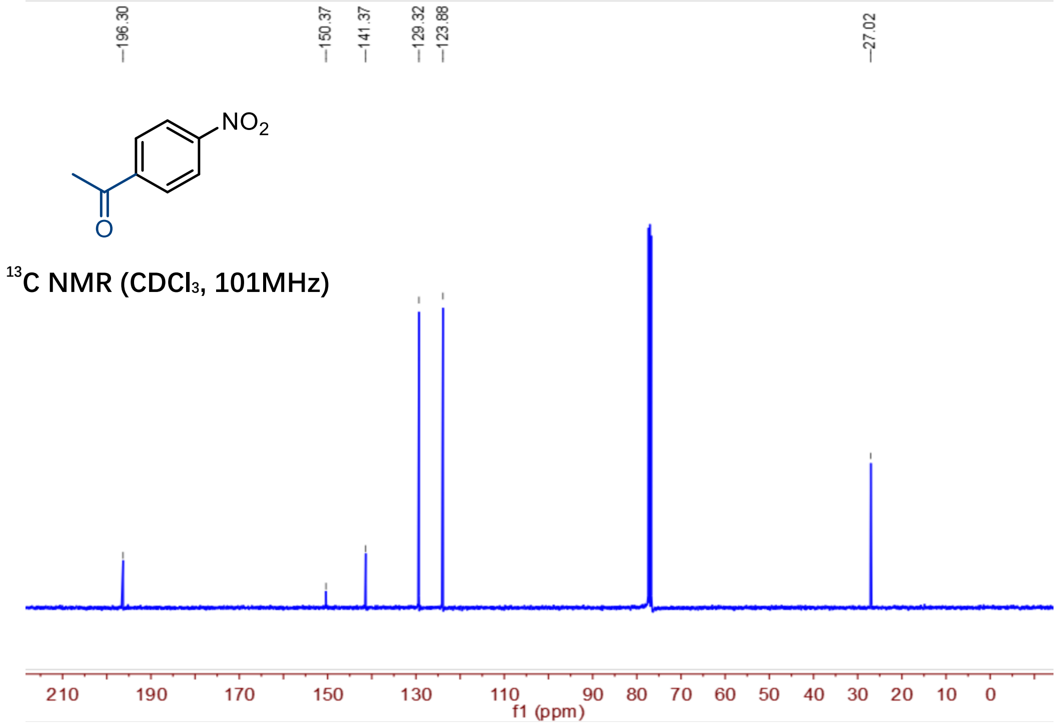

^{13}C NMR Spectrum of Compound 9

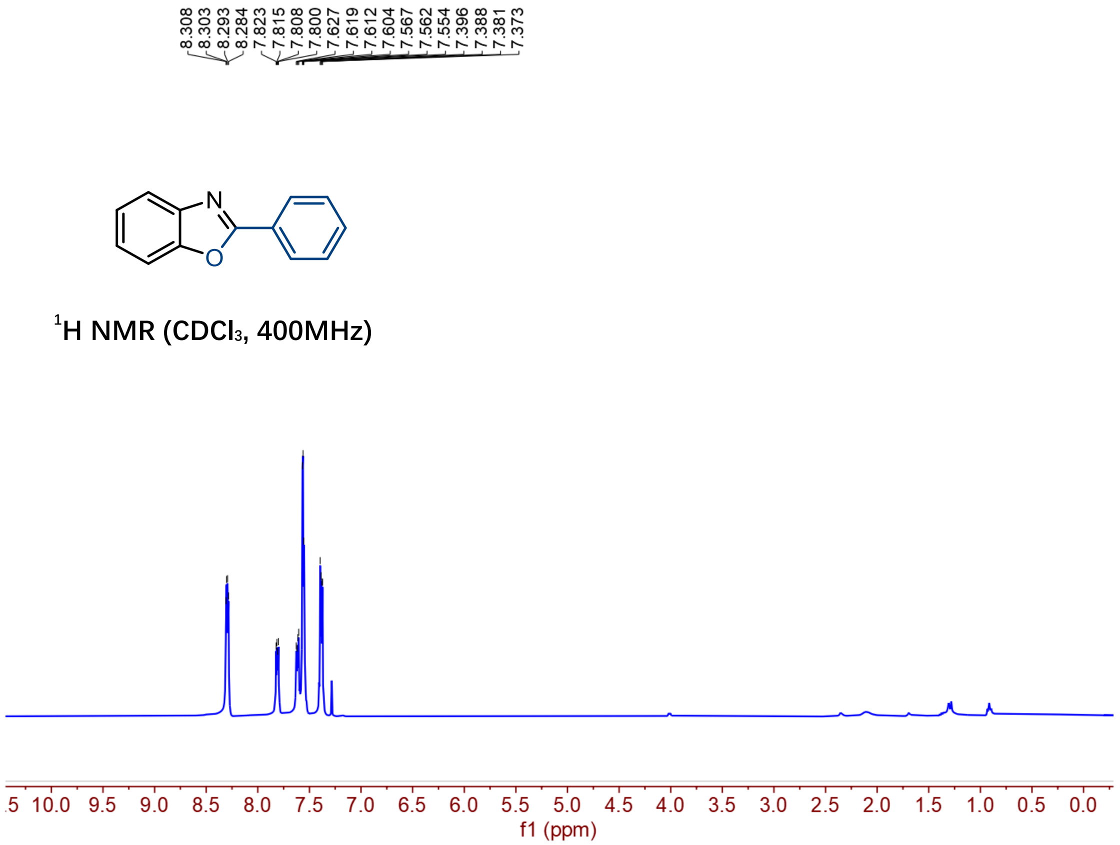

^{1}H NMR Spectrum of Compound 12

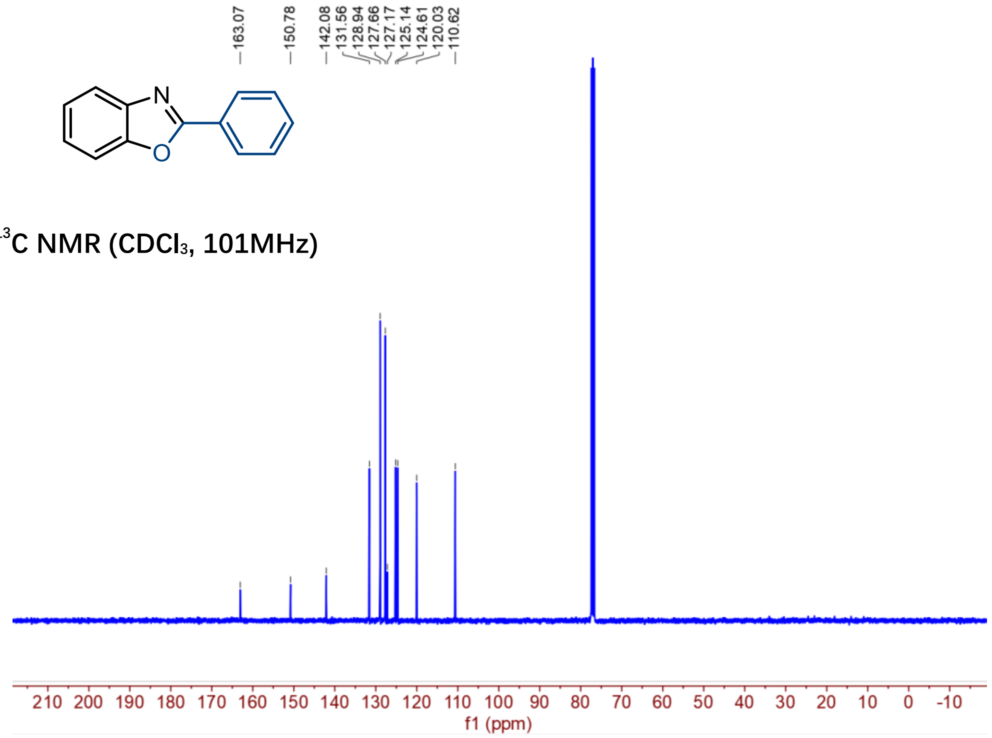

^{13}C NMR Spectrum of Compound 12

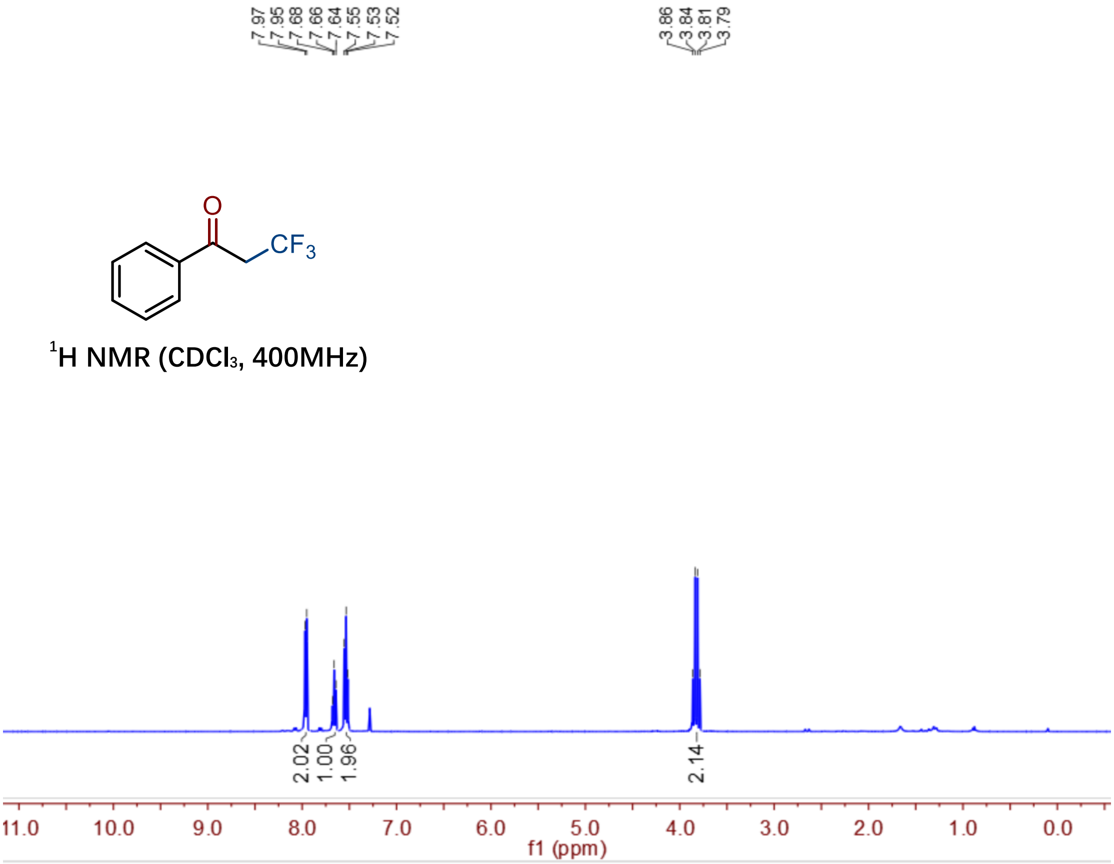

^{1}H NMR Spectrum of Compound 15

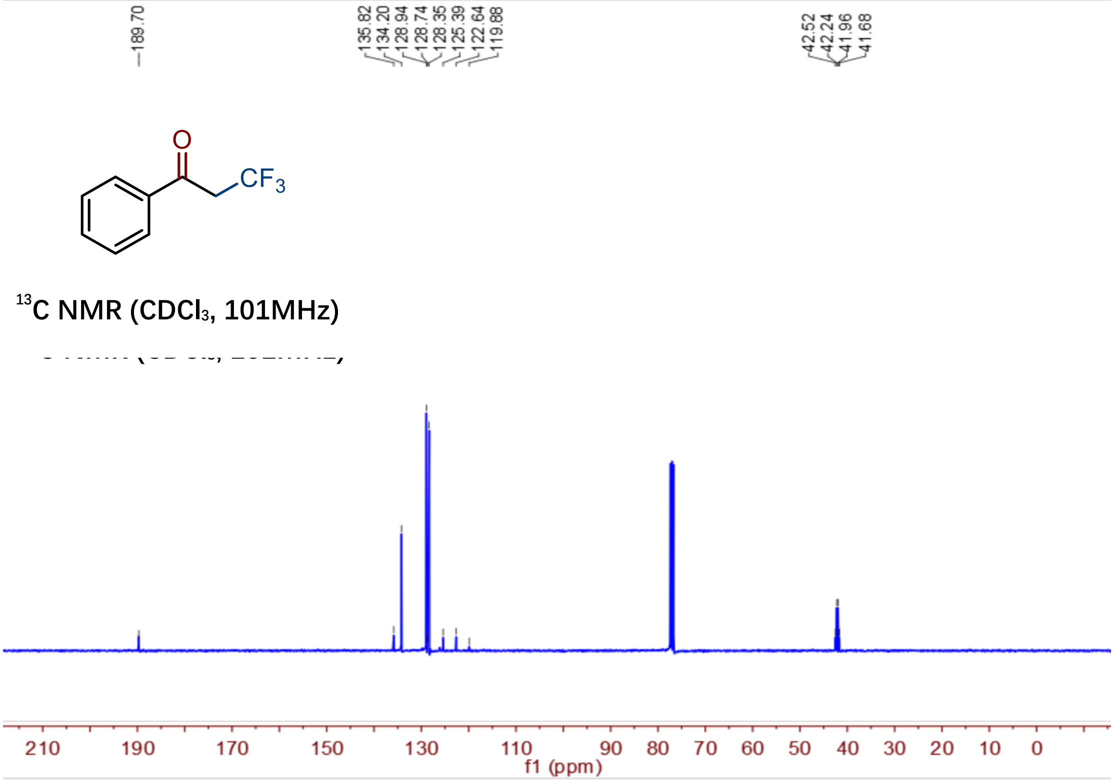

^{13}C NMR Spectrum of Compound 15

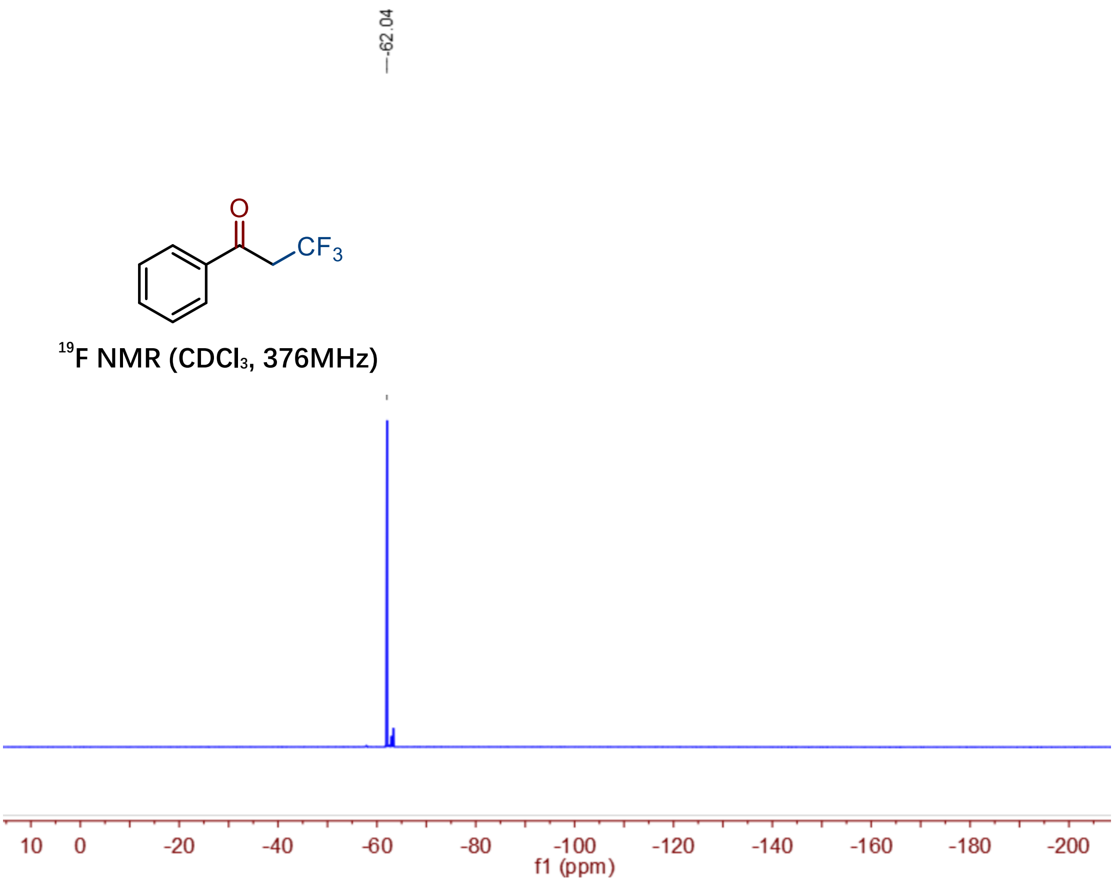

^{19}F NMR Spectrum of Compound 15

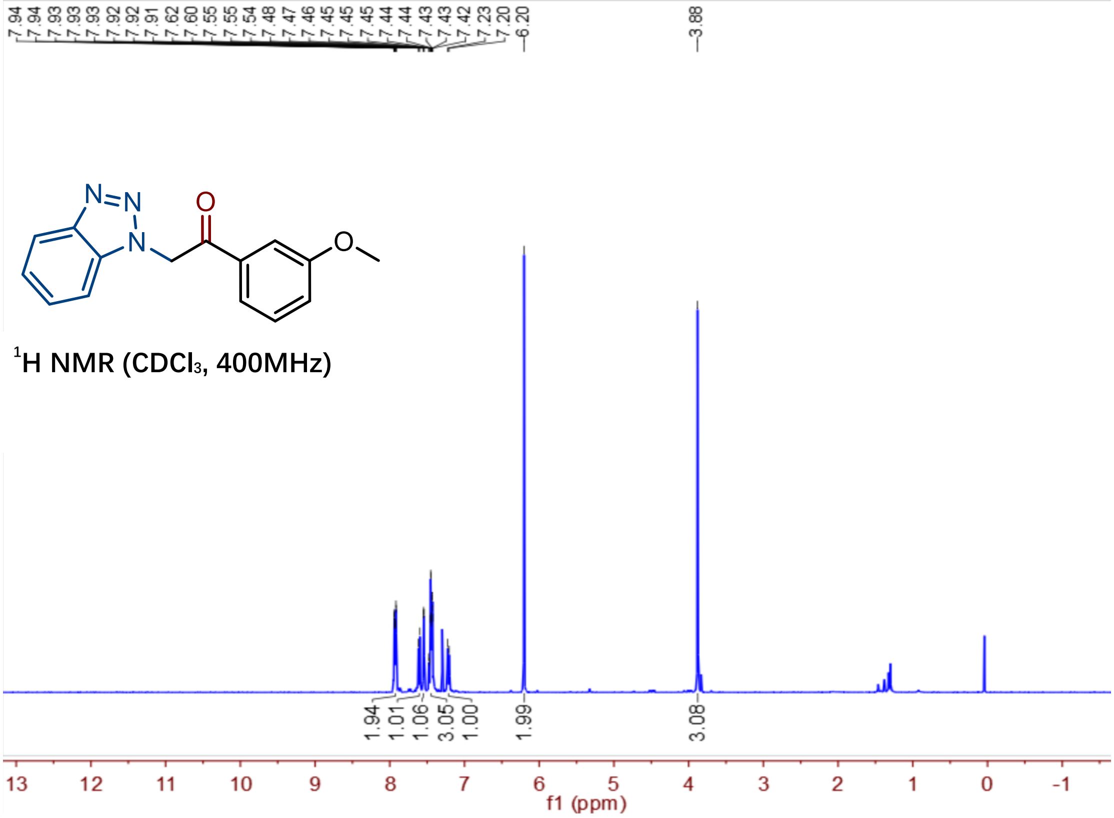

^{1}H NMR Spectrum of Compound 18

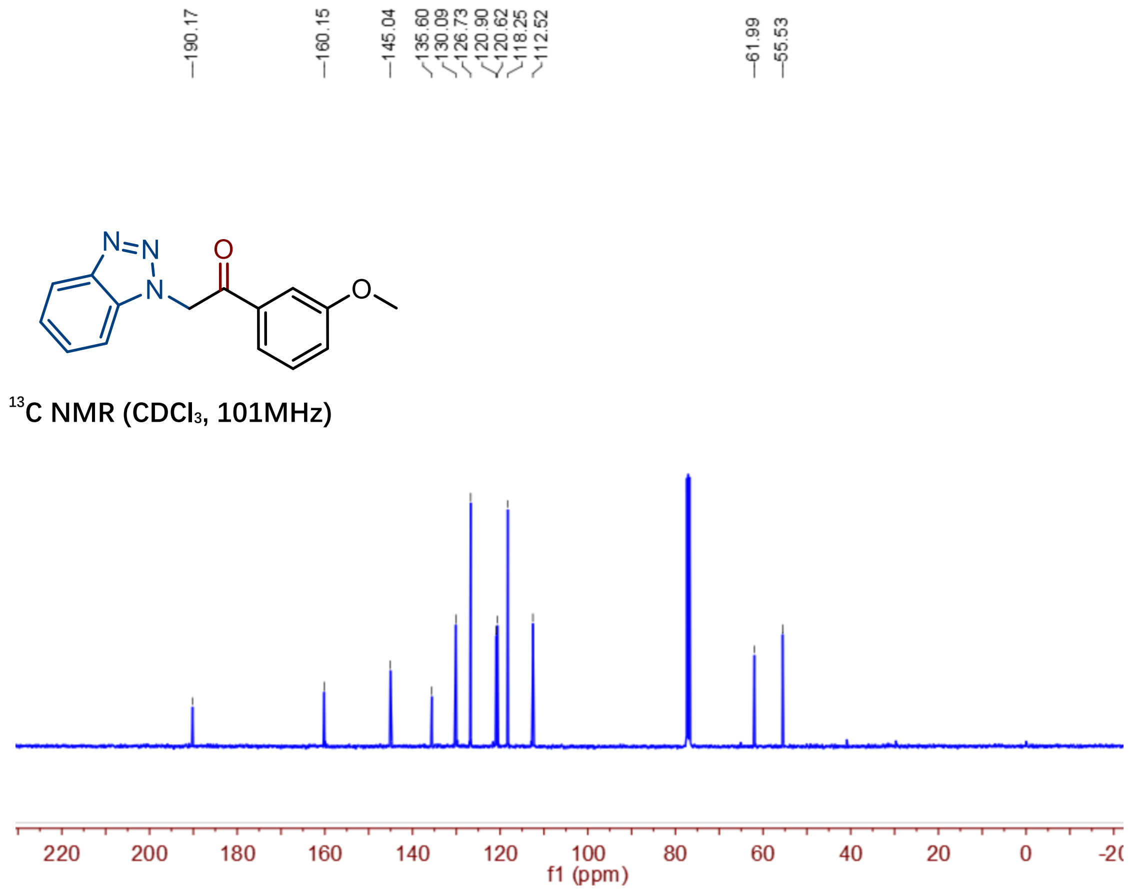

^{13}C NMR Spectrum of Compound 18

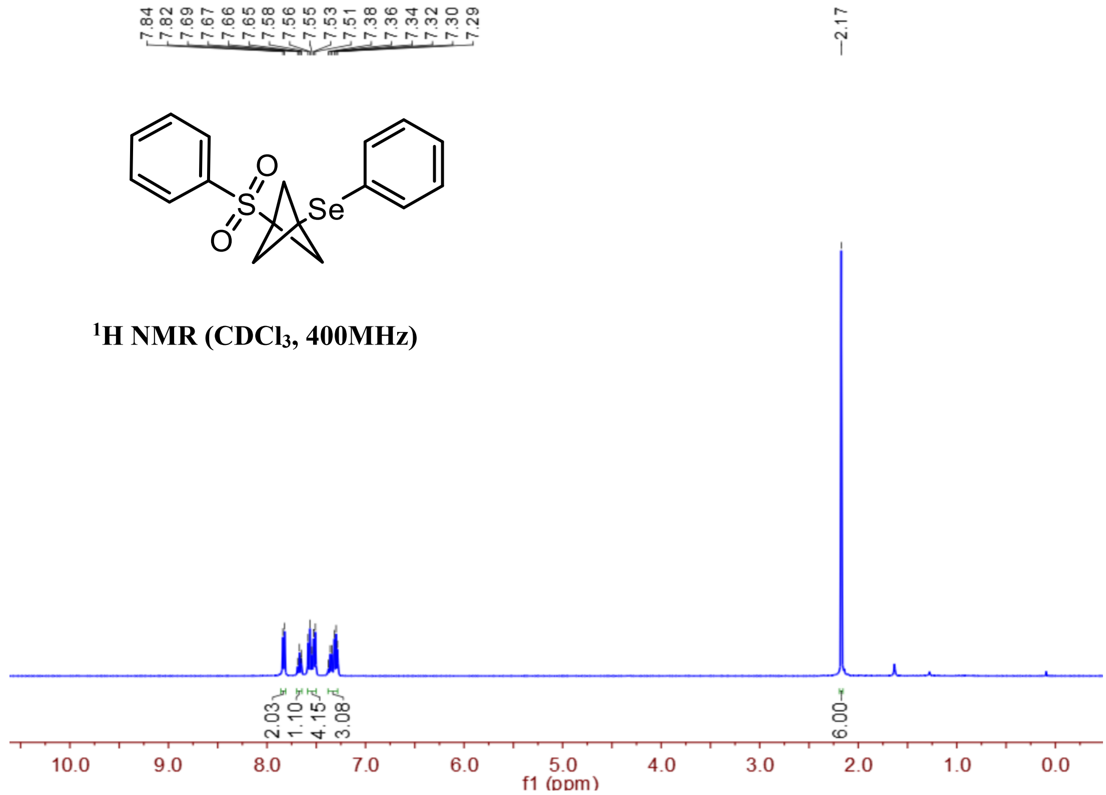

^{1}H NMR Spectrum of Compound 23

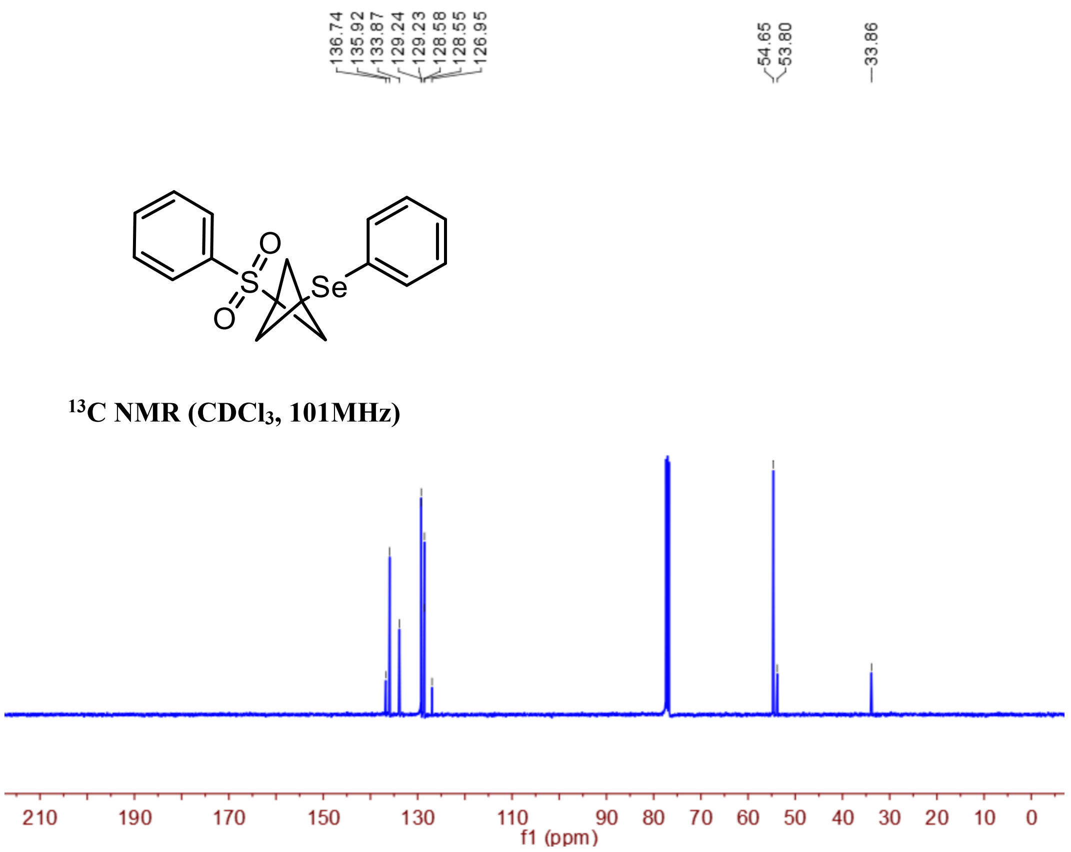

^{13}C NMR Spectrum of Compound 23

4. Supplementary References

(1) Tu, Z.; Coley, C. W. Permutation Invariant Graph-to-Sequence Model for Template-Free Retrosynthesis and Reaction Prediction. *J. Chem. Inf. Model.* **2022**, *62* (15), 3503–3513. https://doi.org/10.1021/acs.jcim.2c00321.

(2) Chapman, S. J.; Swords, W. B.; Le, C. M.; Guzei, I. A.; Toste, F. D.; Yoon, T. P. Cooperative Stereoinduction in Asymmetric Photocatalysis. *J. Am. Chem. Soc.* **2022**, *144* (9), 4206–4213. https://doi.org/10.1021/jacs.2c00063.

(3) Qin, Q.; Han, Y.-Y.; Jiao, Y.-Y.; He, Y.; Yu, S. Photoredox-Catalyzed Diamidation and Oxidative Amidation of Alkenes: Solvent-Enabled Synthesis of 1,2-Diamides and α-Amino Ketones. *Org. Lett.* **2017**, *19* (11), 2909–2912. https://doi.org/10.1021/acs.orglett.7b01145.

(4) Bazyar, Z.; Hosseini-Sarvari, M. Visible-Light-Driven Direct Oxidative Coupling Reaction Leading to Alkyl Aryl Ketones, Catalyzed by Nano Pd/ZnO. *European Journal of Organic Chemistry* **2019**, *2019* (12), 2282–2288. https://doi.org/10.1002/ejoc.201900021.

(5) Ruan, J.; Li, X.; Saidi, O.; Xiao, J. Oxygen and Base-Free Oxidative Heck Reactions of Arylboronic Acids with Olefins. *J. Am. Chem. Soc.* **2008**, *130* (8), 2424–2425. https://doi.org/10.1021/ja0782955.

(6) Qian, W.; Zhang, L.; Sun, H.; Jiang, H.; Liu, H. Microwave-Assisted One-Step

Synthesis of Acetophenones via Palladium-Catalyzed Regioselective Arylation of Vinyloxytrimethylsilane. *Advanced Synthesis & Catalysis* **2012**, *354* (17), 3231–3236. https://doi.org/10.1002/adsc.201200334.
(7) Hou, J.; Ee, A.; Cao, H.; Ong, H.-W.; Xu, J.-H.; Wu, J. Visible-Light-Mediated Metal-Free Difunctionalization of Alkenes with CO2 and Silanes or C(Sp3)−H Alkanes. *Angewandte Chemie International Edition* **2018**, *57* (52), 17220–17224. https://doi.org/10.1002/anie.201811266.
(8) Sun, S.-Z.; Duan, Y.; Mega, R. S.; Somerville, R. J.; Martin, R. Site-Selective 1,2-Dicarbofunctionalization of Vinyl Boronates through Dual Catalysis. *Angewandte Chemie International Edition* **2020**, *59* (11), 4370–4374. https://doi.org/10.1002/anie.201916279.
(9) Sahoo, A. K.; Dahiya, A.; Das, B.; Behera, A.; Patel, B. K. Visible-Light-Mediated Difunctionalization of Alkynes: Synthesis of β-Substituted Vinylsulfones Using O- and S-Centered Nucleophiles. *J. Org. Chem.* **2021**, *86* (17), 11968–11986. https://doi.org/10.1021/acs.joc.1c01350.
(10) Yi, J.; Badir, S. O.; Alam, R.; Molander, G. A. Photoredox-Catalyzed Multicomponent Petasis Reaction with Alkyltrifluoroborates. *Org. Lett.* **2019**, *21* (12), 4853–4858. https://doi.org/10.1021/acs.orglett.9b01747.
(11) Blackwell, J. H.; Kumar, R.; Gaunt, M. J. Visible-Light-Mediated Carbonyl Alkylative Amination to All-Alkyl α-Tertiary Amino Acid Derivatives. *J. Am. Chem. Soc.* **2021**, *143* (3), 1598–1609. https://doi.org/10.1021/jacs.0c12162.
(12) Wang, C.; Ma, D.; Tu, Y.; Bolm, C. Use of Hypervalent Iodine Reagents in Visible Light-Promoted α-Ketoacylations of Sulfoximines with Aryl Alkynes. *Org. Lett.* **2020**, *22* (22), 8937–8940. https://doi.org/10.1021/acs.orglett.0c03338.
(13) Ragupathi, A.; Sagadevan, A.; Lin, C.-C.; Hwu, J.-R.; Hwang, K. C. Copper(i)-Catalysed Oxidative C–N Coupling of 2-Aminopyridine with Terminal Alkynes Featuring a C C Bond Cleavage Promoted by Visible Light. *Chem. Commun.* **2016**, *52* (79), 11756–11759. https://doi.org/10.1039/C6CC05506K.
(14) Wang, X.; Zhu, B.; Dong, J.; Tian, H.; Liu, Y.; Song, H.; Wang, Q. Visible-Light-Mediated Multicomponent Reaction for Secondary Amine Synthesis. *Chem. Commun.* **2021**, *57* (41), 5028–5031. https://doi.org/10.1039/D1CC01560E.
(15) Chalotra, N.; Ahmed, A.; Rizvi, M. A.; Hussain, Z.; Ahmed, Q. N.; Shah, B. A. Photoredox Generated Vinyl Radicals: Synthesis of Bisindoles and β-Carbolines. *J. Org. Chem.* **2018**, *83* (23), 14443–14456. https://doi.org/10.1021/acs.joc.8b02193.
(16) Kolahdouzan, K.; Kumar, R.; Gaunt, M. J. Visible-Light Mediated Carbonyl Trifluoromethylative Amination as a Practical Method for the Synthesis of β-Trifluoromethyl Tertiary Alkylamines. *Chem. Sci.* **2020**, *11* (44), 12089–12094. https://doi.org/10.1039/D0SC04853D.
(17) Pampana, V. K. K.; Sagadevan, A.; Ragupathi, A.; Hwang, K. C. Visible Light-Promoted Copper Catalyzed Regioselective Acetamidation of Terminal Alkynes by Arylamines. *Green Chem.* **2020**, *22* (4), 1164–1170. https://doi.org/10.1039/C9GC03608C.
(18) Shang, T.; Zhang, J.; Zhang, Y.; Zhang, F.; Li, X.-S.; Zhu, G. Photocatalytic Remote Oxyfluoroalkylation of Heteroalkynes: Regio-, Stereo-, and Site-Selective

Access to Complex Fluoroalkylated (Z)-Alkenes. *Org. Lett.* **2020**, *22* (9), 3667–3672. https://doi.org/10.1021/acs.orglett.0c01163.

(19) Pelliccia, S.; Alfano, A. I.; Luciano, P.; Novellino, E.; Massarotti, A.; Tron, G. C.; Ravelli, D.; Giustiniano, M. Photocatalytic Isocyanide-Based Multicomponent Domino Cascade toward the Stereoselective Formation of Iminofurans. *J. Org. Chem.* **2020**, *85* (4), 1981–1990. https://doi.org/10.1021/acs.joc.9b02709.

(20) Guo, L.; Song, F.; Zhu, S.; Li, H.; Chu, L. Syn-Selective Alkylarylation of Terminal Alkynes via the Combination of Photoredox and Nickel Catalysis. *Nature Communications* **2018**, *9* (1), 4543. https://doi.org/10.1038/s41467-018-06904-9.

(21) Nawrat, C. C.; Jamison, C. R.; Slutskyy, Y.; MacMillan, D. W. C.; Overman, L. E. Oxalates as Activating Groups for Alcohols in Visible Light Photoredox Catalysis: Formation of Quaternary Centers by Redox-Neutral Fragment Coupling. *J. Am. Chem. Soc.* **2015**, *137* (35), 11270–11273. https://doi.org/10.1021/jacs.5b07678.

(22) Zhu, C.; Yue, H.; Maity, B.; Atodiresei, I.; Cavallo, L.; Rueping, M. A Multicomponent Synthesis of Stereodefined Olefins via Nickel Catalysis and Single Electron/Triplet Energy Transfer. *Nature Catalysis* **2019**, *2* (8), 678–687. https://doi.org/10.1038/s41929-019-0311-x.

(23) Shang, T.-Y.; Lu, L.-H.; Cao, Z.; Liu, Y.; He, W.-M.; Yu, B. Recent Advances of 1,2,3,5-Tetrakis(Carbazol-9-Yl)-4,6-Dicyanobenzene (4CzIPN) in Photocatalytic Transformations. *Chem. Commun.* **2019**, *55* (38), 5408–5419. https://doi.org/10.1039/C9CC01047E.

(24) Shen, J.; Zhang, Y.; Yu, Y.; Wang, M. Metal-Free Visible-Light-Induced Photoredox-Catalyzed Intermolecular Pyridylation/Phosphinoylation of Alkenes. *Org. Chem. Front.* **2021**, *8* (5), 901–907. https://doi.org/10.1039/D0QO01218A.

(25) Skubi, K. L.; Blum, T. R.; Yoon, T. P. Dual Catalysis Strategies in Photochemical Synthesis. *Chem. Rev.* **2016**, *116* (17), 10035–10074. https://doi.org/10.1021/acs.chemrev.6b00018.

(26) Chen, J.-Y.; Li, K.-M.; Sun, Y.-X.; Xiao, Y.; Guo, F.-S.; Huang, Y.-B.; Lu, Q. An Atomically Dispersed Co Catalyst for Efficient Oxidative Fabrication of Benzoheterocycles under Ambient Oxygen Conditions. *Green Chem.* **2024**, *26* (8), 4834–4843. https://doi.org/10.1039/D4GC00445K.

(27) Huang, P.; Xu, Y.; Song, H.; Wang, J.; Wang, J.; Li, J.; Sun, B.; Jin, C. Selective C(Sp3)–H Bond Aerobic Oxidation Enabled by a π-Conjugated Small Molecule-Oxygen Charge Transfer State. *Green Chem.* **2024**, *26* (16), 9241–9249. https://doi.org/10.1039/D4GC02010C.

(28) Sun, B.; Huang, P.; Yan, Z.; Shi, X.; Tang, X.; Yang, J.; Jin, C. Self-Catalyzed Phototandem Perfluoroalkylation/Cyclization of Unactivated Alkenes: Synthesis of Perfluoroalkyl-Substituted Quinazolinones. *Org. Lett.* **2021**, *23* (3), 1026–1031. https://doi.org/10.1021/acs.orglett.0c04225.

(29) Ji, J.; Liu, P.; Sun, P. Peroxide Promoted Tunable Decarboxylative Alkylation of Cinnamic Acids to Form Alkenes or Ketones under Metal-Free Conditions. *Chem. Commun.* **2015**, *51* (35), 7546–7549. https://doi.org/10.1039/C5CC01762A.

(30) Dong, C.-L.; Ding, X.; Huang, L.-Q.; He, Y.-H.; Guan, Z. Merging Visible Light Photocatalysis and L-/d-Proline Catalysis: Direct Asymmetric Oxidative

Dearomatization of 2-Arylindoles To Access C2-Quaternary Indolin-3-Ones. *Org. Lett.* **2020**, *22* (3), 1076–1080. https://doi.org/10.1021/acs.orglett.9b04613.
